# Chiral Switch of Gadopiclenol: New Standards in MRI Probes

**DOI:** 10.1002/advs.202415321

**Published:** 2025-02-17

**Authors:** Roberta Napolitano, Nicol Guidolin, Mariangela Boccalon, Alberto Fringuello Mingo, Sonia Colombo Serra, Federica Buonsanti, Roberta Fretta, Nicola Demitri, Attila Bényei, Mauro Botta, Giovanni Battista Giovenzana, Fabio Tedoldi, Zsolt Baranyai

**Affiliations:** ^1^ Bracco Research Centre Bracco Imaging SpA Via Ribes 5 Colleretto Giacosa 10010 TO Italy; ^2^ CRB Trieste Bracco Imaging SpA AREA Science Park Basovizza 34149 TS Italy; ^3^ XRD2 Beamline Elettra‐Sincrotrone Trieste S.C.p.A. S.S. 14 Km 163.5 in Area Science Park Basovizza 34149 TS Italy; ^4^ Department of Physical Chemistry University of Debrecen Egyetem tér 1 Debrecen H‐4032 Hungary; ^5^ Dipartimento di Scienze e Innovazione Tecnologica, Piattaforma di Risonanze Magnetiche (PRISMA‐UPO) Università del Piemonte Orientale “A. Avogadro” Viale Teresa Michel 11 Alessandria 15121 Italy; ^6^ Dipartimento di Scienze del Farmaco (DSF) Università del Piemonte Orientale “A. Avogadro” Largo Donegani 2/3 Novara 28100 Italy; ^7^ Headquarters Bracco Imaging SpA Via Egidio Folli 50 Milano 20134 MI Italy

**Keywords:** coordination chemistry, diagnostics, imaging, physical chemistry, stereochemistry, synthesis

## Abstract

Magnetic Resonance Imaging (MRI) plays a vital role in the accurate diagnosis of numerous human diseases and disorders, with Gd(III)‐based contrast agents (GBCAs) being used in ≈30%–40% of procedures, resulting in ≈30 million doses administered annually worldwide. The careful design of a rigid macrocyclic chelator featuring a highly hydrophilic periphery leads to the development of gadopiclenol, the first bis‐hydrated Gd(III)‐based MRI contrast agent, recently approved for clinical use by both the FDA and EMA. The stereochemistry of the coordinating arms is found to play a crucial role in the remarkable thermodynamic stability and inertness of the Gd(III)‐complex with the *RRR/SSS*‐stereoisomer of this heptadentate chelating agent, ensuring its safety in vivo. The exceptional stability of the most effective gadopiclenol enantiomeric pair (*RRR/SSS*), coupled with a relaxivity 2 to 3 times higher than that of currently used GBCAs, has enabled the use of reduced doses while ensuring non‐inferior image contrast.

## Graphical abstract

The stereochemistry of the coordinating side arms of the polyaminocarboxylic ligand piclenol is found to be crucial in determining the MRI contrast efficiency of the corresponding Gd(III)‐complex (“gadopiclenol”). This finding allowed to identify the most effective enantiomer of gadopiclenol, the latter being used as the first clinically approved *q* = 2 Gd‐based contrast agent.

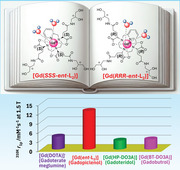

## 1 Introduction

**Figure 1:**
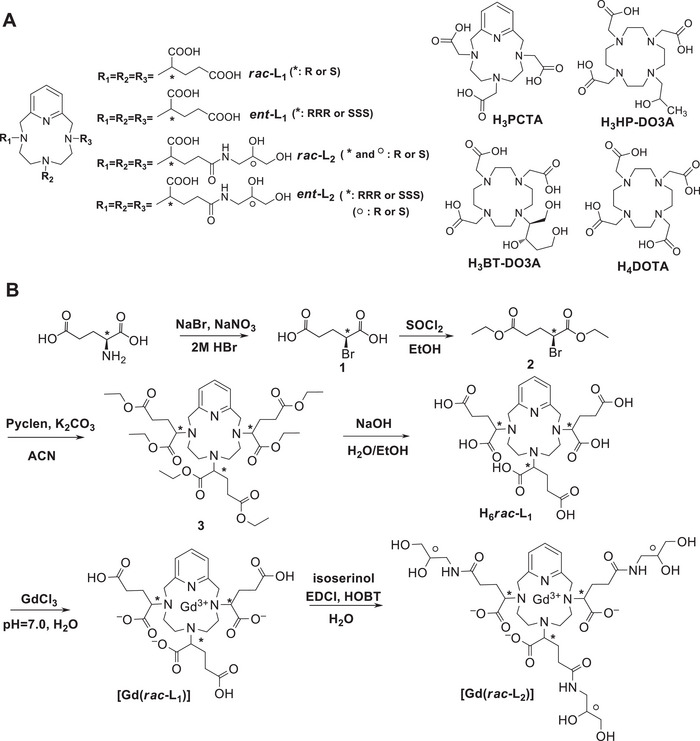
Chelating agents for GBCAs A) and synthesis of [Gd(*rac‐*L_1_)] and [Gd(*rac‐*L_2_)] B). ^*^ and ° represent the stereocenters.

Gd(III) complexes are the most commonly used MRI contrast agents, designed to accelerate proton relaxation rates (primarily water protons) and enhance contrast between healthy and diseased tissues.^[^ ^1^, ^2^, ^3^, ^4^ ^]^ Contrast Enhanced Magnetic Resonance Imaging (CE‐MRI) with Gd(III)‐based contrast agents is crucial for accurate diagnosis, representing a vast fraction (30–40%) of all MR images and corresponding to ≈30m doses/y worldwide.^[^ ^2^ ^]^ GBCAs in clinical practice can be classified as open‐chain (as [Gd(DTPA)]^2−^) or macrocyclic (as [Gd(DOTA)]^−^) (**Figure** **1**A), based on the structure of the chelating agent. Several in vitro and in vivo trials demonstrated the different kinetic inertness of these two subclasses, with macrocyclic GBCAs characterized by significantly higher resistance to dissociation due to their preorganized rigid structure (Figure 1A).^[^ ^3^, ^5^ ^]^ Although GBCAs have been used for over 35 years with minimal clinical side effects, increased signal intensity has been observed in non‐contrast *T*_1_‐weighted MR images of the brain in patients after repeated administration of open‐chain GBCAs.^[^ ^6^ ^]^ This is likely due to the dissociation of the less kinetically inert open‐chain GBCAs. Recommendations to limit the use of open‐chain GBCAs were introduced, leading to a preference for the more kinetically inert macrocyclic GBCAs in MRI. Nevertheless, deposition of trace amounts of Gd(III) has been observed in patients after administration of macrocyclic GBCAs,^[^ ^7^ ^]^ later demonstrated to involve intact Gd(III) complexes mainly.^[^ ^8^ ^]^ Several strategies have been proposed to tackle the in vivo deposition of the GBCAs. An effective approach is the development of CAs based on essential paramagnetic metal ions.^[^ ^9^ ^]^ Mn(II)‐^[^ ^10^, ^11^ ^]^ and Fe(III)‐complexes^[^ ^12^, ^13^, ^14^ ^]^ have been the focus of intense research, with several candidates demonstrating promising properties.^[^ ^15^ ^]^ Despite encouraging results, Mn(II)‐ and Fe(III)‐based MRI probes are still facing challenges such as limited efficacy, insufficient thermodynamic stability, and kinetic inertness.

An alternative strategy is to enhance the efficacy of GBCAs, allowing for a proportional reduction in the administered dose. This can be achieved by improving the parameters that govern GBCA efficacy, quantified as relaxivity (*r*_1_ = the increase in the longitudinal relaxation rate of water protons by a 1 mm GBCA solution). GBCAs enhance both the longitudinal (1/*T*_1_) and transverse (1/*T*_2_) relaxation rates of water protons similarly but are most effective in *T*_1_‐weighted images.^[^ ^1^, ^2^ ^]^ The *r*_1_ values of GBCAs primarily depend on the number of water molecules coordinated to the paramagnetic metal ion (*q*). Achieving higher relaxivity necessitates a chelator with a denticity lower than the coordination number of the metal ion, which often compromises the thermodynamic stability of the resulting metal complex. Furthermore, reducing the ligand's denticity frequently increases the lability of the metal complex (e.g. [Gd(DOTA)]^−^ vs. [Gd(DO3A)]).^[^ ^5^, ^16^ ^]^ Current clinical GBCAs use octadentate ligands, limiting them to just one coordinated water molecule (*q* = 1) and resulting in relatively modest relaxivity values.^[^ ^1^, ^3^ ^]^

An active search is underway for stable and inert Gd(III)‐complexes with hexa and heptadentate ligands with *q* > 1. So far, few chelating agents offer an optimal balance of stability, inertness and enhanced hydration of the resulting Gd(III)‐complexes: i) hydroxypyridinones (HOPOs)^[^ ^17^ ^]^ ii) mesocyclic AAZTAs;^[^ ^18^ ^]^ iii) macrocyclic DO3A derivatives^[^ ^19^, ^20^ ^]^ and iv) macrobicyclic PCTA (PCTA: 3,6,9,15‐tetraazabicyclo[9.3.1]pentadeca‐1(15),11,13‐triene‐3,6,9 triacetic acid) (Figure 1A).^[^ ^21^, ^22^ ^]^

The pyridine ring of PCTA plays the multiple roles of hosting an N‐donor atom and rigidifying the macro(bi)cyclic cage of the corresponding chelates. [Gd(PCTA)] shows high relaxivity due to the presence of two coordinated water molecules (*q* = 2).^[^ ^19^, ^22^ ^]^ However, the inertness of [Gd(PCTA)] is significantly lower than that of [Gd(DOTA)]^−^.^[^ ^5^, ^21^, ^23^, ^24^ ^]^ [Gd(PCTA)] is a suitable platform for the development of *q* = 2 GBCAs, however, a significant improvement of the kinetic inertness is needed to match the requirements for in vivo applications.^[^ ^25^ ^]^

In this work we report the synthesis of chiral PCTA‐derivatives (L_1_ − L_2_) and demonstrate the importance of their stereochemistry, highlighting the large differences observed in the thermodynamic, kinetic, relaxation, and structural properties of the corresponding stereoisomeric Gd(III)‐complexes. PCTA is enriched by placing highly hydrophilic moieties on the coordinating side arms, replacing the carboxymethyl moieties of the archetypal PCTA with α‐glutaric residues (L_1_). The proximal (α−) carboxylic acids of the latter are reserved to the coordination of the metal ion, while the distal (γ−) carboxylic acids are linked to 3‐amino‐1,2‐propanediol (“isoserinol”) moieties, (L_2_). Each side arm of L_2_ bears two stereocenters, related to the α‐glutaric and the isoserinol moieties, respectively, leading to the theoretical possibility of 64 stereoisomers.

This study identified [Gd(*ent*‐L_2_)] as the most effective enantiomer of gadopiclenol (Figure 1A), the latter being used as an innovative and efficient GBCA (Gadopiclenol is the API (active pharmaceutical ingredient) of a contrast media marketed under the brand name Vueway and Elucirem). Pharmacokinetic, biodistribution, and MR imaging data for [Gd(*ent‐*L*_2_*)] are reported, too.

## 2 Results and Discussion

### 2.1 Synthesis of Gd(III)‐Complex with *rac*‐L_1_ and *rac*‐L_2_

The synthesis of [Gd(*rac‐*L_1_] and [Gd(*rac‐*L_2_)] is described in Figure 1B and detailed in the Supporting Information (paragraph “1. Synthesis”).^[^ ^26^ ^]^ The pendant arms were connected to Pyclen (3,6,9,15‐tetraazabicyclo[9.3.1]pentadeca‐1(15),11,13‐triene) by alkylation of the secondary amines with diethyl (2*S*)‐2‐bromoglutarate (2), obtaining hexaester 3. Compound 2 was prepared by combined diazonation/halogenation (NaNO_2_/HBr) of L‐glutamic, followed by esterification with ethanol/thionyl chloride. Alkaline hydrolysis of the ethyl esters of 3 led to the ligand H_6_L_1_, obtained as a mixture of stereoisomers (*rac*‐H_6_L_1_).

RP‐HPLC analysis showed that H_6_ *rac*‐L_1_ is obtained as a mixture of stereoisomers (A: 24%, B: 15%, C: 21%, and D: 40%), as result of the racemization of the α‐glutaric stereocenters. The distribution of the stereoisomer mixture did not change by significantly either changing the hydrolysis conditions or starting from racemic 2. A different distribution of the stereoisomers (A: 1%, B: 19%, C: 78%, and D: 2%) was found when using a different leaving group (2(*S*)‐triflyloxy instead of 2‐bromo) in compound 2, pointing out that the stereochemical integrity of the α‐glutaric residue is lost during the alkylation step.

**Figure 2:**
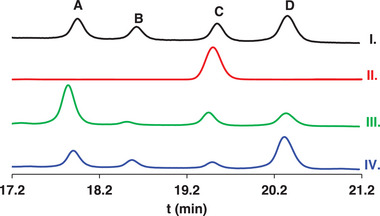
HPLC chromatograms of [Gd(*rac‐*L_1_)] I.), [Gd(*ent*‐L_1_)] obtained by flash chromatography (peak C) II.) and Gd(III)‐complexes with free ligands retrieved from “fiber” III.) and “cubic” IV.) single crystals of the tetraprotonated [Cu(H_4_ *rac‐*L_1_)] complex.

**Figure 3:**
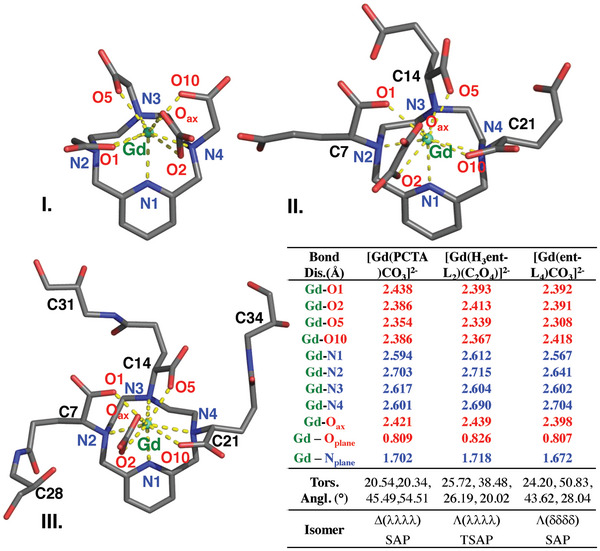
Stick representation, bond distances and angles for the Gd(III) coordination environment in [Gd(PCTA)CO_3_]^2−^ I.), [Gd(H_3_ *ent‐*L_1_)(C_2_O_4_)]^2−^ II.) and [Gd(*ent‐*L_2_)CO_3_]^2−^ III.) complexes found in the single crystals of {(C(NH_2_)_3_)_2_[Gd(PCTA)(CO_3_)]}·4H_2_O, {(C(NH_2_)_3_)_2_[Gd(C_26_H_34_N_4_O_12_)(C_2_O_4_)]}·1H_2_O and {(C(NH_2_)_3_)_2_[Gd(C_35_H_54_N_7_O_15_)(CO_3_)]}·22H_2_O.

RP‐HPLC of [Gd(*rac‐*L_1_)] complex showed four distinct peaks with area percentage ratio A: 24%, B: 15%, C: 21%, and D: 40%, ascribed to as many enantiomeric pairs of stereoisomers (**Figure** **2**‐I). Flash chromatography using a slow gradient of water‐acetonitrile on silica‐C_18_ column allowed to isolate from the racemic mixture the peak C with very high purity (Figure 2‐II), later identified by single crystal X‐ray diffractometric analysis as the *RRR/SSS* enantiomer pair [Gd(*ent‐*L_1_)], **Figures** **3** and S21 (Supporting Information).

The *RRR*‐*SSS* enantiomers pair of Gd(III)‐complex with H_6_ *rac‐*L_1_ related to C in Figure 2‐I was then compared by HPLC with Gd(III)‐complex arising from *SSS* and *RRR* isomers of *ent‐*L_1_ obtained by stereoselective synthesis (Schemes S2 and S3, Supporting Information). The chromatograms in Figure S12 (Supporting Information) confirm that the two enantiomers related to C in Figure 2‐I have the same retention times of the Gd(III)‐complex with *RRR* and *SSS* isomers of *ent‐*L_1_. Moreover, chiral HPLC (Figure S13, Supporting Information) confirms that C contains both *RRR* and *SSS* isomers of [Gd(*ent‐*L_1_)] in 1:1 ratio.

By reacting [Gd(*rac‐*L_1_)] with excess isoserinol, EDCI, and HOBT at pH 6 in water, [Gd(*rac‐*L_2_)] was obtained. After purification either on XAD1600 resin or silica‐C_18_ with a gradient of water‐acetonitrile, pure [Gd(*rac‐*L_2_)] was obtained as an isomeric mixture, giving four groups of peaks by reverse phase chromatography (Figure S14, Supporting Information). In the reaction of [Gd(*rac‐*L_1_)] with enantiopure *R* or *S* isoserinol, [Gd(*rac‐*L_2_)] was obtained as a mixture of isomers showing the same four chromatographic peaks. The coupling of enantiopure *R* or *S* isoserinol with pure *RRR/SSS* [Gd(*ent‐*L_1_)] led to [Gd(*ent‐*L_2_)], corresponding to the chromatographic D’ peak (Figure S14, Supporting Information).

### 2.2 Structural Properties of the Gd(III)‐ and Y(III)‐Complexes with PCTA, *ent*‐L_1_ and *ent*‐L_2_

The crystal structures of [Gd(H_3_ *ent‐*L_1_)], [Gd(*ent‐*L_2_)], and [Gd(PCTA)] were determined by single crystal X‐ray diffractometric analysis. The simplified structure of the [Gd(H_3_ *ent‐*L_1_)], [Gd(*ent‐*L_2_)], and [Gd(PCTA)] complexes with the selected bond distances are shown in Figure 3. In all complexes the Gd(III) ion is coordinated by seven donor atoms of the ligands, whereas the two residual coordination sites are occupied by η^2^‐carbonate or η^2^‐oxalate (Figures S19–S24, Supporting Information). [Gd(H_3_ *ent‐*L_1_)C_2_O_4_]^2−^ crystallized in the *P* 2_1_ */c* space group contains equal amounts of *RRR* and *SSS* stereoisomers. The remote carboxylates of [Gd(H_3_ *ent‐*L_1_)C_2_O_4_]^2−^ are all protonated and non‐coordinated. Conglomerate crystals of [Gd(*ent‐*L_2_)(CO_3_)]^2−^ in the *R* ‐3 space group contain equal amounts of the *RRR‐RRR*, *RRR‐SSS*, *SSS‐RRR* and *SSS‐SSS* stereoisomers. The average Gd─O distance of [Gd(*ent*‐L_2_)CO_3_]^2−^ is significantly shorter than that of [Gd(PCTA)CO_3_]^2−^ indicating a tighter Gd─O interactions due to the favorable helical arrangement of the coordinating side arms sharing the same configurational descriptor. (Gd─O: 2.391 and 2.377 Å for [Gd(PCTA)CO_3_]^2−^ and [Gd(*ent*‐L_2_)CO_3_]^2−^).

To identify the configuration of the stereocenters in the A, B and D peaks of [Gd(*rac*‐L_1_)] (Figure 2‐I), distinct Gd(III)‐complexes have been prepared with ligands retrieved from “fiber” and “cubic” single crystals of tetraprotonated [Cu(H_4_ *rac‐*L_1_)] (Figures S15–S18, Supporting Information) by H_2_S‐mediated demetallation (configuration is *SRS‐RSR* and *RRS‐SSR* in “fiber” and “cubic” crystals of [Cu(H_4_ *rac‐*L_1_)]). HPLC chromatograms of the Gd(III)‐complexes in Figure 2 show that A and D peaks are related to [Gd(*rac*‐L_1_)] with *SRS‐RSR* and *RRS‐SSR* configuration, respectively. As peaks A, C, and D in HPLC chromatogram (Figure 2) correspond to the *SRS‐RSR*, *SSS‐RRR*, and *RRS‐SSR* isomeric pairs, it can be inferred that peak B is the *RSS‐SRR* isomeric pair of [Gd(*rac*‐L_1_)].

Further insights come from the NMR spectra in aqueous solution. At low temperatures, significant broadening of the ¹H and ¹^3^C NMR resonances is observed for [La(PCTA)] and [Lu(PCTA)],^[^ ^27^ ^]^ attributed to the ring‐wagging motion of the NCH₂CH₂N moieties. Similar broadening is also seen for [Y(PCTA)] (Figure S25, Supporting Information). Since Y^3^⁺ has an ionic radius closest to that of Gd^3^⁺, the structural properties of the Y(III) complexes of *ent*‐L_1_ and *ent*‐L_2_ were investigated by NMR (Figures S30–S37, Supporting Information). The ¹H NMR signals of the ethylene groups in the rings of [Y(*ent*‐L_1_)]^3−^ and [Y(*ent*‐L_2_)] (Figures S30 and S34, Supporting Information) remain virtually unchanged over a wide temperature range (T = 278–333 K), underscoring the remarkable structural rigidity of the macrocyclic ring, which is stabilized by the helical arrangement of the coordinating side arms.

### 2.3 Thermodynamic Properties of the Ca(II)‐, Zn(II)‐, Cu(II)‐ and Gd(III)‐Complexes

Stability and conditional stability constants of Ca(II)‐, Zn(II)‐, Cu(II)‐ and Gd(III)‐complexes with *rac*‐L_1_, *ent*‐L_1_, *rac*‐L_2_ and *ent*‐L_2_ ligands obtained by pH‐potentiometry, spectrophotometry and ^1^H NMR relaxometry are compared with those of the related PCTA, DOTA, HP‐DO3A and BT‐DO3A complexes (**Table** **1**; Table S18, Supporting Information). The log*K*_ML_, log*K* ^c^_GdL_ and pGd values of Ca(II)‐, Zn(II)‐, Cu(II)‐ and Gd(III)‐complexes with *rac*‐L_1_ and *rac*‐L_2_ can be assumed as the weighted average of the stability and conditional stability constants of the metal complexes with the distinct stereoisomers.

**Table 1:** Stability (log*K*_ML_) and conditional stability (log*K* ^c^_GdL_, pGd) constants of the Ca(II)‐, Zn(II)‐, Cu(II)‐ and Gd(III)‐complexes with *rac‐*L_1_, *ent‐*L_1_, *rac‐*L_2_, *ent‐*L_2_, PCTA, DOTA, HP‐DO3A and BT‐DO3A. Standard deviations (3σ) are shown in parentheses. Dissociation rate (*k*_d_) and half‐life (*t*_1/2_ = ln2/*k*_d_) values of the Gd(III) complexes at pH 7.4 and 25 °C. The descriptors refer to the configuration (*) of the stereocenters in Figure 1.

	*ent‐*L_2_	*rac‐*L_2_	*ent‐*L_1_	*rac‐*L_1_	PCTA^b)^	DOTA^c)^	HP‐DO3A^f)^	BT‐DO3A^g)^
I	0.15 m NaCl				1.0 m KCl	0.1 m KCl	0.1 m Me_4_NCl	0.1 m NaCl
log*K*_CaL_	10.26 (1)	7.84 (2)	11.03 (1)	10.10 (1)	12.72	16.37	14.83	12.1
log*K*_ZnL_	20.13 (3)	18.66 (3)	20.77 (2)	19.54(2)	20.48	18.7	19.37	17.0
log*K*_CuL_	21.52 (4)	21.04 (5)	23.22 (6)	23.26(4)	18.79	22.72	22.84	19.1
log*K*_GdL_	20.36 (3)	18.96 (7)	20.17 (9)	18.65(5)/ 18.66(Eu^3+^)^a)^	20.39	24.7^d)^ / 25.6^e)^	23.8	18.7/ 21.8^h)^
pGd^k)^	18.27	17.70	17.80	16.33 / 16.06 (Eu^3+^)^a)^	17.11	22.09^d)^ / 20.24^e)^	18.16	15.63
log*K* ^c^_GdL_ ^l)^	17.31	16.76	16.85	15.34 / 15.10 (Eu^3+^)^a)^	16.15	21.14^d)^ / 19.29^e)^	17.21	14.67/ 15.5^h)^
*k*_d_ [s^−1^] at pH = 7.4	*RRR/SSS* (D′): 1.74 × 10^−13^	*RSR/SRS* (A′): 1.85 × 10^−12^ *RSS/SRR* (B′): 8.55 × 10^−12^ *RRS/SSR* (C′): 2.42 × 10^−13^	*RRR/SSS* (C): 5.61 × 10^−14^	*RSR/SRS* (A): 1.84 × 10^−12^ *RSS/SRR* (B): 1.28 × 10^−11^ *RRS/SSR* (D): 4.95 × 10^−13^	2.0 × 10^−11^ ([Eu(PCTA)])	7.3 × 10^−14^ ^i)^ 3.3 × 10^−14^ ^j)^	1.2 × 10^−11^ ^i)^ 1.0 × 10^−11^ ^g)^	1.34 × 10^−12^
*t*_1/2_ [h] at pH = 7.4	*RRR/SSS* (D′): 1.11 × 10^9^	*RSR/SRS* (A′): 1.04 × 10^8^ *RSS/SRR* (B′): 2.25 × 10^7^ *RRS/SSR* (C′): 7.95 × 10^8^	*RRR/SSS* (C): 3.4 × 10^9^	*RSR/SRS* (A): 1.08 × 10^8^ *RSS/SRR* (B): 1.50 × 10^7^ *RRS/SSR* (D): 3.89 × 10^8^	9.52 × 10^6^ ([Eu(PCTA)])	2.64 × 10^9^ ^i)^ 5.80 × 10^8^ ^j)^	1.67 × 10^7^ ^i)^ 1.86 × 10^7^ ^g)^	1.42 × 10^8^

^a)^ Ref. [28] (0.1 M Me_4_NCl, 25 °C);

^b)^ Ref. [21];

^c)^ Ref. [29];

^d)^ Ref. [30] (0.1 M NaCl, 25 °C);

^e)^ Ref. [31] (0.1 M Me_4_NCl, 25 °C);

^f)^ Ref. [32] (0.1 M Me_4_NCl, 25 °C);

^g)^ Ref. [33] (0.1 M NaCl, 25 °C);

^h)^ Ref. [33] (0.1 M KCl, 25 °C);

^i)^ Ref. [5] (0.15 M NaCl, 25 °C);

^j)^ Ref. [34] (25 °C);

^k)^ pGd = ‐log[Gd^3+^]_free_, [Gd^3+^]_tot_ = 1 µm, [L]_tot_ = 10 µm, pH 7.4;

^l)^ *K*_GdL_^c^ = *K*_GdL_/1+α_H_, α_H_ = *K*_1_^H^[H^+^]+ *K*_1_^H^ *K*_2_^H^[H^+^]^2^+… *K*_1_^H^ *K*_2_^H^…*K*_n_^H^[H^+^]^n^ and pH 7.4.

log*K*_ML_ values of the metal complexes with *ent‐*L_1_ and *ent‐*L_2_ are generally 0.5–2.0 log*K* unit higher than those of the corresponding *rac‐*L_1_ and *rac‐*L_2_, except for Cu(II) (Table 1). Moreover, stability constants of the Ca(II)‐, Zn(II)‐ and Gd(III)‐complexes with *ent‐*L_1_ and *ent‐*L_2_ are very similar. The higher stability of the Ca(II)‐, Zn(II)‐ and Gd(III)‐complexes with *ent‐*L_1_ and *ent‐*L_2_ compared to the racemic counterparts could be explained by the tighter coordination imparted by the helical arrangement of the coordinating side arms sharing the same (*RRR* or *SSS*) configuration. Although this factor is primarily associated with an enthalpic contribution, the possibility of an entropic role cannot be ruled out. Surprisingly the Gd(III)‐complexes of *ent‐*L_1_ and *ent‐*L_2_ are characterized by 1.5 log*K* unit higher stability constant than that of the racemic [Gd(*rac‐*L_1_)]^3−^ and [Gd(*rac‐*L_2_)]. Considering the species distribution of [Gd(*rac‐*L_1_)]^3−^ (*RSR/SRS* = 24%, *RSS/SRR* = 15%, *RRR/SSS* = 21%, *SSR/RRS* = 40%, Figure 2), and the stability constant of [Gd(*rac‐*L_1_)]^3−^ and [Gd(*ent‐*L_1_)]^3−^ (Table 1), the weighted average stability constant of the [Gd(*rac‐*L_1_)]^3−^ complex with *RSR/SRS*, *RSS/SRR* and *SSR/RSS* configuration is log*K*_GdL_ = 18.26, two orders of magnitude lower than that of [Gd(*ent‐*L_1_)]^3−^. Based on the log*K*_Gd(_ *_rac‐_*_L2)_ and log*K*_Gd(_ *_ent‐_*_L2)_ values in Table 1, the higher selectivity for Gd^3+^ of *ent‐*L_1_ than *rac‐*L_1_ is preserved after the reaction with isoserinol.

The comparison of the log*K* ^c^_GdL_ and pGd values reveals that the Gd(III)‐complexes with *ent‐*L_1_, *rac‐*L_2,_ and *ent‐*L_2_ have significantly higher conditional stability than [Gd(BT‐DO3A)] and [Gd(PCTA)], and comparable stability with [Gd(HP‐DO3A)]. Conditional stability values of [Gd(*ent‐*L_1_)]^3−^ and [Gd(*ent‐*L_2_)] are 0.6 – 1.5 log*K* unit higher than those of the racemic counterparts, confirming the positive effect of the chiral switch.

### 2.4 Kinetic Inertness of the Gd(III)‐Complexes

The kinetic inertness of metal complexes is a key parameter to assess their safety in vivo. The dissociation of macrocyclic Gd(III)‐complexes is extremely slow and generally takes place by acid‐catalyzed reactions (*k*_1_, Table S19, Supporting Information) through protonated intermediates (log*K* ^H^_GdHL_, Table S19, Supporting Information). Endogenous metal ions have a negligible contribution to the decomplexation rate of the macrocyclic Gd(III)‐complexes.^[^ ^5^, ^16^, ^23^, ^35^ ^]^ Dissociation reactions of Gd(III)‐complexes in 0.01–1.0 m HCl solution to establish pseudo‐first‐order kinetic conditions were monitored by HPLC. Rate (*k*_i_), protonation constants (log*K* ^H^_GdHL_), and dissociation half‐lives (*t*_1/2_ = ln2/*k*_d_ at pH = 7.4) characterizing the dissociation of the different stereoisomers formed by [Gd(*rac*‐L_1_)]^3−^ and [Gd(*rac*‐L_2_)] are compared with those of [Gd(PCTA)], [Gd(DOTA)]^−^, [Gd(HP‐DO3A)] and [Gd(BT‐DO3A)] (Table 1; Table S19, Supporting Information).

The proton‐assisted dissociation of the Gd(III)‐complexes with the different stereoisomers of *rac*‐L_1_ and *rac*‐L_2_ is significantly slower than that of [Gd(PCTA)]. Acid‐catalyzed dissociation of the Gd(III)‐complex with the *RRR*/*SSS* stereoisomer of the heptadentate *rac*‐L_1_ (*ent*‐L_1_) and *rac*‐L_2_ (*ent*‐L_2_) are 24, 207, 7, and 66 times slower than that of Gd(III)‐complexes with the octadentate BT‐DO3A and HP‐DO3A, respectively. Moreover, the proton‐assisted dissociation of [Gd(*ent*‐L_1_)]^3−^ and [Gd(*ent*‐L_2_)] are comparable with that of the “gold standard” [Gd(DOTA)]^−^. Dissociation presumably occurs with the proton transfer from the ‐COOH to the ring N‐atom resulting in the substitution of the Gd^3+^ ion by the H^+^ in the coordination cage. Dissociation reactions of Ln(III)‐complexes with similarly α‐substituted DOTA analogs are also slower than that of [Ln(DOTA)]^−^.^[^ ^36^ ^]^ Finally, half‐lives (*t*_1/2_ = ln2/*k*_d_) calculated at pH 7.4 (Table 1; Table S19, Supporting Information) prove that the inertness of [Gd(*ent*‐L_1_)] is higher, whereas the *t*_1/2_ value of [Gd(*ent*‐L_2_)] is comparable, with that of [Gd(DOTA)]^−^ in physiological conditions.

### 2.5 Relaxation Properties of the Gd(III) Complexes

**Figure 4:**
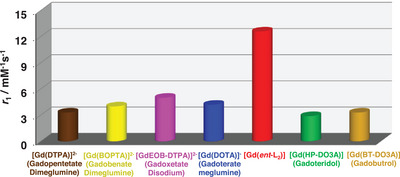
Relaxivity values of [Gd(*ent*‐L_2_)] and of clinical contrast agents at 310 K and 1.47 T in water. *r*_1_ values of [Gd(DTPA)]^2−^, [Gd(BOPTA)]^2−^, [Gd(EOB‐DTPA)]^2−^, [Gd(HP‐DO3A)] and [Gd(BT‐DO3A)] were taken from Ref. [26, 37]

**Figure 5:**
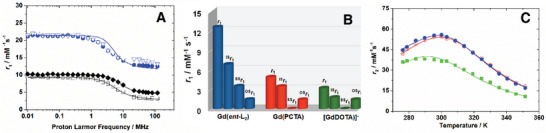
^1^H NMRD profiles A), relative contributions to relaxivity B) and ^17^O NMR transverse relaxivity (*r*_2_) as a function of temperature C) A: [Gd(DOTA)]^−^ (◻), [Gd(PCTA)] (◆) at 310 K in water, and [Gd(*ent‐*L_2_)] at 310 K in water (•) and in human plasma (▽); B: 310 K and 1.5 T; C: [Gd(*rac*‐L_1_)]^3−^ (◼), [Gd(*ent*‐L_1_)]^3−^ (•) and [Gd(*ent*‐L_2_)] (⊕) at 67.8 MHz (11.74 T) and pH = 7.2 in water.

The paramagnetic relaxation theory suggests that Gd(III) complexes can achieve relaxivity (*r*_1_) values much higher than current clinical MRI contrast agents (ca. 3–3.5 mm ^−1^ s^−1^ at 1.47 T and 310 K, **Figure** **4**).^[^ ^1^, ^3^, ^38^, ^39^ ^]^ Relaxivity depends on parameters like molecular tumbling, hydration number (*q*), water exchange rate, and electronic relaxation time.^[^ ^1^, ^3^ ^]^ Increasing *q* from 1 to 2 has been explored but is limited by: a) the formation of ternary complexes with biological oxyanions displacing water molecules; and b) reduced stability and inertness of less dentate chelating agents.^[^ ^19^, ^40^ ^]^ [Gd(*ent*‐L_2_)] overcomes these issues, emerging as a highly efficient clinical MRI probe while remaining a stable and inert *q* = 2 complex. Comparing *r*_1_ values (Figure 4 and **Table** **2**) of [Gd(*rac*‐L_1_)]^3^⁻, [Gd(*ent*‐L_1_)]^3^⁻, and [Gd(*ent*‐L_2_)] with [Gd(DOTA)]⁻ (*q* = 1) and [Gd(PCTA)] (*q* = 2), we noticed that relaxivity does not correlate with molecular mass among *q* = 2 complexes (Figure S56, Supporting Information). For instance, [Gd(*rac*‐L_1_)]^3^⁻ and [Gd(*ent*‐L_1_)]^3^⁻ show an 18% difference despite being isomers, while [Gd(*ent*‐L_2_)] has 175% higher *r*_1_ than [Gd(PCTA)] despite only an 81% mass increase,^[^ ^25^ ^]^ and a 309% increase compared to [Gd(DOTA)]⁻ (**Table** **2**).^[^ ^36^, ^41^ ^]^ Across all frequencies, [Gd(*ent*‐L_2_)] shows an unexpected additional contribution to *r*_1_, far exceeding predicted values (**Figure** **5**A).

**Table 2:** Selected relaxation parameters of the Gd(III) complexes at 298 and 310 K in water.

	^298K^ *r*_1_ [mm ^−1^s^−1^] [1.5 T]	*r*_1_^310K^ [mm ^−1^s^−1^] [1.5 T]	*τ*_M1_ [ns]	*τ*_M2_ [ns]	*τ*_R_ [ns]	*q*	^SS^ *q*
[Gd(*rac*‐L_1_)]^3−^	9.4	6.8	103 ± 5	14 ± 2	98 ± 2	2	/
[Gd(*ent*‐L_1_)]^3−^	10.9	8.6	144 ± 4	58 ± 5	96 ± 3	2	2.5 ± 0.1
[Gd(*ent*‐L_2_)]	16.5	12.6	155 ± 3	77 ± 5	127 ± 4	2	4.5 ± 0.2
[Gd(PCTA)]^a)^	6.4	4.9	151	36	63	2	/
[Gd(DOTA)]^−^ ^b)^, ^c)^	4.2	3.3	208	/	90	1	/

^a)^ Ref. [25];

^b)^ Ref. [36];

^c)^ Ref. [41].

The NMRD profiles of the Gd(III) complexes (Figures S51–S53, Supporting Information) provide a highly accurate estimate of the parameters that determine relaxivity (Table 2; Table S20, Supporting Information).

Relaxivity has two main contributions: inner‐sphere (IS) and outer‐sphere (OS).^[^ ^1^, ^3^ ^]^ IS arises from the interaction between the Gd^3^⁺ ion and its coordinated water molecules, while OS describes long‐range interactions with bulk water. Relaxation parameters align with the complexes' structures: rotational times match molecular size, electronic relaxation times are similar, and coordination cages are comparable. All complexes have two coordinated water molecules (*q* = 2) at 3.0 Å and exhibit rapid exchange, indicated by decreased *r*_1_ with increasing temperature. The high relaxivity of [Gd(*ent*‐L_1_)]^3^⁻ and [Gd(*ent*‐L_2_)] is largely due to a second‐sphere (SS) effect, involving water molecules with strong, long‐lived H─bonds near the complexes. The SS contribution originates from structured solvent molecules in the second coordination shell of the metal ion. These molecules engage in relatively strong and long‐lived hydrogen‐bonding interactions with the polar groups of the ligand. When the distance between the water protons and the metal ion is sufficiently short (≈<4 Å) and the interaction lifetime exceeds roughly 500 ps, the resulting dipolar interaction can lead to a significant enhancement in relaxation.^[^ ^42^, ^43^, ^44^, ^45^ ^]^ This effect, equivalent to 2.5 ([Gd(*ent*‐L_1_)]^3^⁻) and 4.5 ([Gd(*ent*‐L_2_)]) SS water molecules at 3.6 Å, is linked to pendant arms that organize H─bonded networks near the paramagnetic center. [Gd(*ent*‐L_2_)] combines a standard OS contribution, increased IS relaxivity from reduced tumbling (longer *τ*_R_), higher hydration, and a substantial SS effect, resulting in high relaxivity at clinical imaging fields (Figure 5B).

The NMRD profile of [Gd(*ent*‐L_2_)] in human plasma (Figure 5A; Table S21, Supporting Information) and in Seronorm (Figure S55, Supporting Information) at 310 K showed negligible changes in relaxivity, suggesting no significant interactions with proteins or oxyanions, confirmed by anion titration (Figure S54, Supporting Information). Exchange rates of the coordinated water molecules were measured;^[^ ^38^ ^]^ [Gd(*rac*‐L_1_)]^3^⁻ displayed faster water exchange than [Gd(*ent*‐L_1_)]^3^⁻ and [Gd(*ent*‐L_2_)], likely due to greater steric strain at binding sites (Figure 5C and Table 2).

### 2.6 Biodistribution Studies and In Vivo MRI

**Figure 6:**
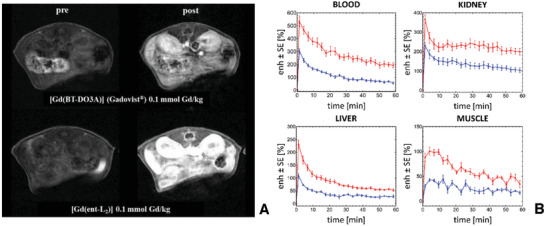
Representative images pre and post contrast administration for each Gd(III) complexes (all the images are reported with the same black and white scale) A) and signal enhancement (averaged over groups) ± standard error as a function of time B). Each panel represents a different anatomical region, while curves referring to different Gd(III) complexes are plotted in different colors (blue for [Gd(BT‐DO3A)] (n = 6), red for [Gd(*ent*‐L_2_)] (n = 6)), both the CAs were administered at the same dose, i.e. 0.1 mmol Gd/kg. Statistical analysis: Levene's test, one‐way ANOVA, Tukey's post‐hoc (the statistical significance level (𝛼) was set at 0.05). Software: Mathematica (Wolfram, USA).

Dynamic *T*_1_‐weighted MRI on twelve A/J healthy mice was used to compare the biodistribution of ([Gd(*ent*‐L_2_)] and [Gd(BT‐DO3A)] (Gadovist), *n* = 6 animals each), administered at 0.1 mmol Gd/kg. The MRI monitored the distribution within abdominal tissues for up to 1 h post‐administration MRI images and the pharmacokinetic curves are shown in **Figure** **6**.

Biodistribution results showed that both agents exhibited rapid wash‐in and wash‐out; more specifically a substantial decrease (≥50%) in signal enhancement was observed in the blood compartment within 30 min. Due to the low molecular weight and lack of plasma protein binding of [Gd(*ent‐*L_2_)], renal elimination remains the primary pathway, while the hepatic route plays a less significant role. The study verified a strong correlation between the observed MRI enhancement and the relaxivity values of the two agents, with the *r*_1_​ of [Gd(*ent*‐L_2_)] being more than two times higher than that of [Gd(BT‐DO3A)] (Gadovist) at 37 °C and 3 T. Indeed, [Gd(*ent‐*L_2_)] exhibits a maximum signal enhancement ≈2 times higher than that of [Gd(BT‐DO3A)] when the two agents are administered in the same dose.

In the blood compartment, the maximum signal enhancement of [Gd(*ent*‐L_2_)] is statistically different (≈2 times higher) compared to that of [Gd(BT‐DO3A)] at the same dose, with significance levels of 0.1%, respectively. The kinetics of [Gd(BT‐DO3A)] and [Gd(*ent*‐L_2_)] appear comparable.

In the kidney, the kinetics of both [Gd(BT‐DO3A)] and [Gd(*ent*‐L_2_)] exhibit a rapid wash‐in followed by a wash‐out, consistent with observations in the respective blood compartment. In the liver, [Gd(*ent*‐L_2_)] demonstrates an average maximum signal enhancement of ≈210%, while a lower maximum signal enhancement is observed for [Gd(BT‐DO3A)] administered at the same dose, ≈110% (with a significance level of 0.1%).

The group administered with [Gd(BT‐DO3A)] maintains this difference over time with respect to [Gd(*ent*‐L_2_)], with significance levels ranging between 1% and 5% at the different time points. The kinetics of both administered contrast agents in the liver are rapid, with wash‐in and wash‐out occurring simultaneously, and comparable to observations in the blood compartment. Signal enhancement continuously decreases throughout the observation window (i.e., 60 min). Similar results in terms of enhancement, wash‐in, and wash‐out are observed in the muscle compartment.

## 3 Conclusion

This study systematically identified the most effective stereoisomers of gadopiclenol, contributing to its development as the first bis‐hydrated Gd(III)‐based MRI contrast agent. Gadopiclenol's chelating agent is a chiral, rigidified macrocyclic PCTA derivative with a highly hydrophilic periphery. The configuration of the stereocenters near the coordinating carboxyl groups proved to be crucial in determining the Gd(III) complex's physicochemical properties. The *RRR*/*SSS* stereoisomeric pair (*ent*‐L_2_) exhibited remarkable thermodynamic stability, inertness, and relaxivity, with a stability constant two orders of magnitude higher than the *RSR*/*SRS*, *RSS*/*SRR*, and *SSR*/*RSS* pairs.

Furthermore, the dissociation rate of [Gd(*ent*‐L_2_)] is comparable to that of the gold standard [Gd(DOTA)]^−^, despite its lower denticity. To our knowledge, *ent*‐L_2_ is the only heptadentate ligand to form a GBCA with this exceptional level of inertness. The high relaxivity observed for [Gd(*ent‐*L_2_)] (+309% *vs* [Gd(DOTA)]^−^) is attributed to the two fast‐exchanging inner‐sphere water molecules and a significant contribution from second‐sphere water molecules. The relaxivity remains unaffected by biologically relevant anions, eliminating the risk of undesired ternary complexes under physiological conditions, as confirmed by single crystal X‐ray diffractometric and NMR analyses, which reveal a more compact coordination environment. The superior relaxation enhancement of [Gd(*ent*‐L_2_)] is confirmed by in vivo MR imaging, showing a maximum signal enhancement nearly twice that of [Gd(BT‐DO3A)] at the same dose.

This study highlights the crucial role of stereochemistry in chelating agents, providing a clear example of a successful chiral switch. It led to the identification of [Gd(*ent*‐L_2_)] as the most effective stereoisomer of gadopiclenol.

## 4 Experimental Section

### General

All commercially available reagents used in the synthesis were obtained from Sigma Aldrich and TCI and they were used without further purification. All the reactions were monitored by HPLC or HPLC‐MS (Agilent mod. 1100 or 1260, Quadrupole LC/MS Mod. 6120) equipped with quaternary pump, degasser, autosampler, and a PDA detector set at different wavelengths using the methods described in Table S1 (Supporting Information) (for *rac‐*L_1_, *ent‐*L_1_, [Gd(*rac‐*L_1_)] and [Gd(*ent‐*L_1_)]) and Table S2 (Supporting Information) (for [Gd(*rac‐*L_2_), *ent‐*L_2_ and [Gd(*ent‐*L_2_)]). Mass spectra were recorded using electrospray positive and negative modes, and alternate scans. TLC was performed on Merck 0.25 mm Kieselgel 60 F254 plates. Products were visualized under UV light and/or by staining with aqueous cerium‐molibdate solution.

Flash chromatography was performed on automated systems (CombiFlash Rf+ or CombiFlash NEXTGEN 300+, Teledyne), using pre‐packed cartridges (Redisept or Biotage).

NMR analyses were performed on a Bruker AV600 (^1^H, 600.13 MHz; ^13^C, 150.92 MHz) equipped with a PABBO 600S3 BBF‐H‐D‐05 Z SP probehead or on a Bruker Avance III 400 (9.4 T) equipped with BB inverse z gradient probe (5 mm). The chemical shifts are expressed in ppm relative to tetramethylsilane.

### Equilibrium Measurements

The chemicals used for the experiments were of the highest analytical grade. The concentration of the CaCl_2_, ZnCl_2_, CuCl_2_, YCl_3_ and GdCl_3_ solutions were determined by complexometric titration with standardized Na_2_H_2_EDTA and *xylenol orange* (ZnCl_2_, YCl_3_ and GdCl_3_), *murexide* (CuCl_2_) and *Patton & Reeder* (CaCl_2_) as indicators. The concentration of the *rac‐*L_1_, *ent‐*L_1_, *rac‐*L_2_, *ent‐*L_2,_ and PCTA ligands was determined by pH‐potentiometric titration in the presence and absence of a large (40‐fold) excess of CaCl_2_. The pH‐potentiometric titrations were made with standardized 0.2 m NaOH.

The protonation constants of ligands, the stability and protonation constants of Ca(II)‐ and Zn(II)‐complexes formed with *rac‐*L_1_, *ent‐*L_1_, *rac‐*L_2_, and *ent‐*L_2_ ligands were determined by pH‐potentiometric titration. The metal‐to‐ligand concentration ratio was 1:1 (the concentration of the ligand was generally 0.002 m). The protonation constants of the Cu(II)‐ and Gd(III)‐complexes were determined using pH‐potentiometry by titrating the pre‐prepared complexes from pH 2.0 to 12.0 with 0.2 m NaOH. The stability constants of the Gd(III)‐complexes with *rac‐*L_1_ and *ent‐*L_1_ ligands were determined by the “out‐of‐cell” technique because of their slow formation reaction. The pH range of the complexation equilibria and the time needed to reach the equilibria were determined by relaxometry. Eight–eight Gd(III) – *rac‐*L_1_ and Gd(III) – *ent‐*L_1_ samples were prepared, with pH values in the range of 1.7–3.0 at equilibrium ([Gd^3+^] = [L] = 0.002 m). The samples were kept at 25 °C for 6 weeks to reach equilibrium. For the calculation of the stability constants of the [Gd(*rac‐*L_1_)]^3−^ and [Gd(*ent‐*L_1_)]^3−^ complexes, besides the protonation constants of ligands and the Gd(III) complexes, the stability constants of the di‐protonated [^*^Gd(H_2_ *rac‐*L_1_)]^−^ and [^*^Gd(H_2_ *ent‐*L_2_)]^−^ out‐of‐cage complexes (considered as intermediates) was also used as fixed values, which were calculated from the pH‐potentiometric titration curve of the Gd(III) – *rac‐*L_1_ and Gd(III) – *ent‐*L_1_ system obtained in the pH range of 1.7–7.0.

For the pH measurements and titrations, the *Metrohm 888 Titrando* titration workstation *Metrohm‐6.0234.110* combined electrode was used. Equilibrium measurements were carried out at a constant ionic strength (0.15 m NaCl) in 6 mL samples at 25 °C. The solutions were stirred, and N_2_ was bubbled through them. The titrations were made in the pH range 1.7–12.0. KH‐Phthalate (pH 4.005) and borax (pH 9.177) buffers were used to calibrate the pH meter. For the calculation of [H^+^] from the measured pH values, the method proposed by *Irving et al.* was used as follows.^[^ ^46^ ^]^ A 0.01m HCl solution was titrated with standardized NaOH solution at 0.15 m NaCl ionic strength. The differences (*A*) between the measured (pH_read_) and calculated pH (‐log[H^+^]) values were used to obtain the equilibrium H^+^ concentration from the pH values measured in the titration experiments (*A* = 0.02). For the equilibrium calculations, the stoichiometric water ionic product (p*K*_w_) was also needed in order to calculate [OH^−^] values under basic conditions. The V_NaOH_ – pH_read_ data pairs of the HCl – NaOH titration obtained in the pH range 10.5–12.0 were used to calculate the p*K*_w_ value (p*K*_w_ = 13.83).

The stability constants of the Cu(II)‐complexes with *rac‐*L_1_, *ent‐*L_1_, *rac‐*L_2_ and *ent‐*L_2_ ligands were determined by spectrophotometry studying the Cu(II) – *rac‐*L_1_, Cu(II) – *ent‐*L_1_, Cu(II) – *rac‐*L_2_ and Cu(II) – *ent‐*L_2_ systems at the absorption band of Cu(II)‐complexes at [H^+^] = 0.01–1.0 m in the wavelength range of 400–800 nm. The concentrations of CuCl_2_, *rac‐*L_1_, *ent‐*L_1_, *rac‐*L_2_ and *ent‐*L_2_ ligands were 0.002 m. The H^+^ concentration in the samples was adjusted with the addition of calculated amounts of 3 m HCl_._ (I = [Na^+^]+[H^+^] = 0.15, [H^+^]≤0.15 m). The samples were kept at 25 °C for a week. The absorbance values of the samples were determined at 11 wavelengths (575, 595, 615, 635, 655, 675, 695, 715, 735, 755, and 775 nm). For the calculations of the stability and protonation constants of the Cu(II)‐complexes, the molar absorptivities of CuCl_2_ and Cu(II)‐complexes were determined by recording the spectra of 1.0 × 10^−3^, 1.5 × 10^−3^, 2.0 × 10^−3^, and 2.5 × 10^−3^ m solutions of CuCl_2_ and Cu(II)‐complexes in the pH range of 1.7–7.5. The pH was adjusted by stepwise addition of concentrated NaOH or HCl solutions. The spectrophotometric measurements were made with a *PerkinElmer Lambda 365* UV–vis spectrophotometer at 25 °C, using 1.0 cm cells.

The relaxivity values were calculated from the longitudinal relaxation time of H_2_O protons (*T*_1_) measured at 21 MHz on a *Stelar* relaxometer connected to a *Bruker WP80 NMR* electromagnet adapted to variable‐field measurements (15−80 MHz proton Larmor frequency). The temperature of the sample holder was controlled with a thermostatted air stream. The longitudinal relaxation time was measured with the “inversion recovery” method (180° – *τ* – 90°) by using 12 different *τ* values with typical 90° pulse width of 6.4 µs, 4 scans. The measurements were performed with 1 mm solution of the Gd(III)‐complexes with *rac‐*L_1_, *ent‐*L_1_, *rac‐*L_2,_ and *ent‐*L_2_ ligands, so the relaxivity values were given as *r*_1_ = 1/*T*_1p_ + 1/*T*_1w_ where *T*_1p_ and *T*_1w_ were the relaxation time of bulk water protons in the presence and absence of paramagnetic species, respectively. The variable pH relaxivity measurements of Gd(III)‐complexes with *rac‐*L_1_, *ent‐*L_1_, *rac‐*L_2_, and *ent‐*L_2_ ligands could be carried out by direct titration of the samples in the pH range 2.5–9.0 (21 MHz, 25 °C, 0.15 m NaCl). The pH was adjusted by stepwise addition of concentrated NaOH or HCl solution.

The stability constant of Gd(III)‐complexes with *rac‐*L_2_ and *ent‐*L_2_ ligands were determined by the measurement of the proton relaxation rates (*r*_1_) studying the competition reaction of *rac‐*L_2_ and *ent‐*L_2_ ligands with AAZTA for Gd(III) ion at pH = 4.0 in 0.15 m NaCl solution. In these experiments, the concentration of Gd^3+^, *rac‐*L_2_, and *ent‐*L_2_ ligands was 2.0 mm, while that of the AAZTA was varied between 1.0 and 6.0 mm (5 × 1.5 mL samples). The pH was adjusted to pH 4.0 by stepwise addition of concentrated NaOH or HCl solutions. The samples were kept at 70 °C for a month and then at 25°C for two months in order to attain the equilibrium (the time needed to reach the equilibria was determined by ^1^H‐NMR relaxometry). ^1^H‐NMR relaxometric measurement of Gd(III) – *rac‐*L_2_ – AAZTA and Gd(III) – *ent‐*L_2_ – AAZTA systems were performed with a *Bruker Avance III 400* (9.4 T) equipped with BB inverse z gradient probe (5 mm). The longitudinal relaxation time (*T*_1_) was measured with the “inversion recovery” method (180° – *τ* – 90°) by using 12 different *τ* values with typical 90° pulse width of 12.0 µs, 4 scans at 298 K. The stability constants of [Gd(*rac‐*L_2_)] and [Gd(*ent‐*L_2_)] complexes were calculated by considering the protonation constants of AAZTA ligand and the stability constant of [Gd(AAZTA)]^−^ complex (AAZTA: log*K*_1_^H^ = 10.06, log*K*_2_^H^ = 6.50, log*K*_3_^H^ = 3.77, log*K*_4_^H^ = 2.33, log*K*_5_^H^ = 1.51; [Gd(AAZTA)]^−^: log*K*_GdL_ = 18.93, 0.15 m NaCl, 25 °C).^[^ ^47^ ^]^ The equilibrium constants were calculated with the program *PSEQUAD*.^[^ ^48^ ^]^

### Kinetic Studies

The kinetic inertness of the Gd(III)‐complexes with the different isomers formed by *rac‐*L_1_ and *rac‐*L_2_ was characterized by the rates of the dissociation reactions taking place in 0.01–1.0 m HCl solution. The dissociation reactions were followed by measuring the integral values of the Gd(III)‐complexes with the different isomers of *rac‐*L_1_ and *rac‐*L_2_ ligands with HPLC. The conditions of the HPLC experiments for the kinetic studies of the Gd(III)‐complexes with *rac‐*L_1_ and *rac‐*L_2_ ligands are summarized in Tables S1 and S2 (Supporting Information), respectively. The samples were kept at 25 °C in a well‐closed vessel. For the HPLC analysis, 100 µL samples were withdrawn from the solutions. The measurements were performed with 0.2 mm solution of Gd(III)‐complexes. The pseudo‐first‐order rate constants (*k*_d_) were calculated by fitting the HPLC integral – *t* data pairs to Equation (1).

(1) $$A_{t}=\left(A_{r}-A_{v}\right)\;e^{\left(-k_{d}t\right)}+A_{v}$$

where *A*_r_, *A*_v_, and *A*_t_ are the integral values of the reactants, the product, and reaction time *t*. The temperature was maintained at 25 °C and the ionic strength of the solutions was kept constant at [H^+^]≤0.15 m, [HCl]+[NaCl] = 0.15 m. The calculation of the kinetic parameters was performed by the fitting of the absorbance – time and relaxation rate – time data pairs with the *Micromath Scientist* computer program (version 2.0, Salt Lake City, UT, USA).

### ^1H^ NMRD Profiles

The magnetic‐field dependence of the longitudinal relaxation rate of water protons (^1^H NMRD) were measured in aqueous solution by using a variable field relaxometer equipped with an HTS‐110 3T Metrology Cryogen‐free Superconducting Magnet (Mede, Italy), operating in the overall range of proton Larmor frequencies of 20–120 MHz (0.47–3.00 T). The measurements were performed using the standard inversion recovery sequence (20 experiments, 3 scans) with a 90° pulse width of 3.5 µs. The temperature was controlled with a Stelar VTC‐91 heater airflow. Additional points in the 0.01–10 MHz frequency range were collected on a Fast‐Field Cycling (FFC) Stelar SmarTracer Relaxometer. *T*_1_ values at 500 MHz were collected on a Bruker Avance III NMR spectrometer operating at 11.7 T. The concentration of Gd^3+^ in the solutions was determined by using bulk magnetic susceptibility (BMS) shift measurements performed at 11.7 T. For the measurements in the biological fluid, lyophilized plasma matrix (Siemens Healthcare) was added to 1 mL of [Gd(*ent‐*L_2_)] (1.0 mm) or Seronorm (Sero) was solubilized in 5 mL [Gd(*ent‐*L_2_)] (1.0 mm) solution. ^1^H NMRD of the final solution was measured at 310 K.

### ^17O^ NMR Measurements

The spectra were acquired on a Bruker Avance III spectrometer (11.7 T) using a 5 mm probe under temperature control. An aqueous solution of the complexes ([GdL] = 10–15 mm) was enriched to ca. 2.0% of the ^17^O isotope (Cambridge Isotope). The transverse relaxation rates (*R*_2_) were measured as a function of temperature in the 278−350 K range. The simultaneous fit of ^1^H NMRD and ^17^O NMR profiles data was performed using the Micromath Scientist software (version 2.0, Salt Lake City, UT, USA).

### High Resolution NMR Spectroscopy

Variable temperature ^1^H and ^13^C NMR spectra of Y(III)‐complexes with PCTA, *rac‐*L_1_, *ent‐*L_1_, *ent‐*L_2_ and PCTA ligands were recorded with the Bruker Avance III (9.4 T) spectrometer, equipped with Bruker Variable Temperature Unit (BVT), Bruker Cooling Unit (BCU) and a BB inverse *z* gradient probe (5 mm) in the temperature range of 273–343 K. For these experiments, a 0.1 m solution of Y(III) complexes in 0.15 NaCl aqueous solution was prepared (a capillary with D_2_O was used for lock). The pH was adjusted to 7 by stepwise addition of concentrated NaOH and HCl solutions (both prepared in H_2_O). The ^13^C (zgig), ^1^H‐^1^H correlation spectroscopy (COSY), exchange spectroscopy (EXSY), and ^1^H‐^13^C correlation spectra (HSQC) were collected by using gradient pulses in the *z* direction with the standard Bruker pulse program. The chemical shifts are reported in ppm, with respect to TMS for ^1^H and ^13^C as an external standard (0 ppm for both cases).

### X‐Ray Diffraction Studies

Single crystal X‐ray diffractometric data collection of the [Gd(PCTA)(CO_3_)]^2−^ single crystals with the formula {(C(NH_2_)_3_)_2_[Gd(PCTA)(CO_3_)]}·4H_2_O was carried out at 298 K using Mo‐Kα radiation (λ = 0.71073 Å) with a *Burker‐Nonius MACH3* diffractometer equipped with point detector. Data collections of the [Cu(H_4_ *rac‐*L_1_)], [Gd(H_3_ *ent‐*L_1_)(C_2_O_4_)]^2−^ and [Gd(*ent‐*L_2_)(CO_3_)]^2−^ single crystals with the formulas [Cu(C_26_H_34_N_4_O_12_)] · 4H_2_O (“fiber”), [Cu(C_26_H_34_N_4_O_12_)] · H_2_O (“cubic”), {(C(NH_2_)_3_)_2_[Gd(C_26_H_34_N_4_O_12_)(C_2_O_4_)]}·1H_2_O and {(C(NH_2_)_3_)_2_[Gd(C_35_H_54_N_7_O_15_)(CO_3_)]}·18H_2_O were performed at the XRD1 and XRD2 beamlines of the Elettra Synchrotron, Trieste (Italy).^[^ ^49^ ^]^ The crystals were dipped in NHV oil (Jena Bioscience, Jena, Germany) and mounted on the goniometer head with kapton loops (MiTeGen, Ithaca, USA). Complete datasets were collected at 100 K (nitrogen stream supplied through an Oxford Cryostream 700) through the rotating crystal method. Data were acquired using monochromatic wavelengths of 0.700Å on Pilatus hybrid‐pixel area detectors (DECTRIS Ltd., Baden‐Daettwil, Switzerland). The diffraction data were indexed, integrated and scaled using XDS.^[^ ^50^ ^]^ Two different crystals have been merged using CCP4‐Aimless code, to obtain a complete set of data for [Cu(H_4_ *rac‐*L_1_)] triclinic packing and [Gd(H_3_ *ent‐*L_1_)(C_2_O_4_)]^2−^. The X‐ray crystallographic data had been deposited at the Cambridge Crystallographic Data Centre with deposition numbers 2368968 ‐ 2368973 for *RRS‐* *SSR* racemate, *RSR* and *SRS* enantiomers of [Cu(H_4_ *rac*‐L_1_)], [Gd(*ent*‐L_2_)(CO_3_)]^2‐^, [Gd(H_3_ *ent*‐L_1_)(C_2_O_4_)]^2‐^ and [Gd(PCTA)(CO_3_)]^2‐^, respectively. These data can be obtained free of charge from The Cambridge Crystallographic Data Centre via https://www.ccdc.cam.ac.uk/structures/ and Fachinformationszentrum Karlsruhe Access Structures service. All other data supporting the findings of these studies are available in Supplementary Information.

### Biodistribution and In Vivo MRI Studies

All the procedures and the experiments were conducted according to the national and international laws on experimental animal research (L.D. 26/2014; Directive 2010/63/EU) and under a specific Italian Ministerial Authorization (project research number 46/2015‐PR). Twelve A/J healthy mice (females, 6–7 weeks old, supplied by Envigo RMS S.r.l.) were housed in a controlled environment (22°C, 55% humidity, 15–20 air changes/hour, 12‐h light/dark cycle) with ad libitum access to VRF1 (P) certified pellets and filtered water. To minimize discomfort, the animals were socially housed with enrichment items. Veterinary care was available throughout the study to alleviate any potential pain or distress following procedures. Only mice that showed no signs of pain, distress, or significant weight loss during pre‐experimental observation were deemed eligible for the experimental phase. The animals were randomly assigned to two experimental groups (n = 6 each, with sample size confirmed via GPower) and underwent to Magnetic Resonance Imaging (MRI) experiments performed using a BioSpec 47/30 preclinical scanner (Bruker Biospin, Ettlingen, Germany) operating at 3 T (i.e., at a proton Larmor frequency of 125 MHz) and equipped with two‐channel 35 mm RF volume coil. During MRI experiments, all the animals were anaesthetized with isoflurane gas (≈1%) in O_2_. Anaesthesia was maintained by adjustment of gas level as a function of breath rate. Before injection of each test article, *T*_1_‐weighted spin echo sequences were acquired on the animal in order to check the position of the animal. A series of *T*_1_‐weighted 3D gradient echoscans (repetition time = 50 ms, flip angle = 50°, echo time = 2.8 ms, number of averages = 2, matrix size (3D) = 192 × 192 × 8, field of view = 2.5 × 2.5 × 1.2 cm, acquisition time = 154 s) were then acquired before and after the intravenous administration of a 50 mM saline solution (0.9%) of [Gd(*ent‐*L_2_)] (*n* = 6 mice) and [Gd(BT‐DO3A)] (Gadovist, Bayer Schering, Germany) (n = 6 mice) at an injection rate of ≈2 mL/min through a catheter placed in the tail vein of the animal. Both [Gd(*ent‐*L_2_)] and [Gd(BT‐DO3A)] were administered at a dose of 0.1 mmol Gd/kg corresponding to an administration volume of 2 mL kg^−1^. The kinetics of the Gd(III)‐complexes was followed up to 60 min post‐injection. After imaging was performed, the mouse was removed from the scanner and returned to the Animal Facility room. At the end of the study all animals were sacrificed by overdose of anaesthesia and cervical dislocation. Unblinded image analysis was performed by positioning the region of interest (ROIs) over liver, kidney, muscle, and blood vessel. ROIs positioning and signal quantification was performed by using a home‐developed plugin, running on Image J.^[^ ^51^ ^]^

In the *statistical analysis* pre‐processing of data involved the use of box plots to detect anomalous data points within groups. Data points outside 1.5 times the interquartile range (IQR) from the quartiles were judged as outliers, which were further evaluated and, if necessary, excluded (no significant outliers were detected).

*Signal enhancement* (Enh) was calculated using the formula: Enh = 100×(Signal_postCA_ ‐Signal_preCA_)/Signal_preCA_, where Signal_preCA_ and Signal_postCA_ denote the MR signal before and after administration of contrast agents, respectively. Data were presented as mean ± standard deviation (SD). The sample size (n = 6 per group) was determined a priori using GPower software to ensure adequate statistical power. The Levene's test was employed to assess the homogeneity of variances among groups. For comparisons where homogeneity of variance was confirmed, a one‐way ANalysis Of VAriance (ANOVA) was applied to test the null hypothesis that treatment groups originate from the same distribution. Post‐hoc pairwise multiple comparisons were performed using Tukey's method when significant differences were detected. Statistical analyses and data visualization were conducted using Mathematica (Wolfram, USA), while summary statistics (mean, SD, and standard error) were calculated using Microsoft Excel (USA). The statistical significance level (𝛼) was set at 0.05.

## Conflict of Interest

R.N., N.G., M. B., A. F. M., S. C. S., F. B., R. F., F. T. and Zs. B. are employees of Bracco Group, which markets Vueway.

## Author Contributions

R.N. and G.B.G. conducted the synthetic experimental work. N.G. and M.B. (Bracco) performed the equilibrium, kinetic, and structural studies in solution. A.F.M. and S.C.S. conducted the MRI measurements and the biodistribution studies. N.D. and A.B. conducted the X‐ray diffraction experiments. M.B. (UPO) carried the relaxometric measurements. F.B., R.F., and F.T. performed the interpretation of data and critically reviewed the manuscript. Z.B. conceived the study, and interpreted the data. All authors wrote, finalized, and approved the final version of the manuscript.

## Supporting information



Supporting Information

## Data Availability

The data that support the findings of this study are available in the supplementary material of this article.
