# Supplementary material for: Chiral Switch of Gadopiclenol: New Standards in MRI Probes
Source: Adv Sci (Weinh). 2025 Feb 17;12(14):2415321. doi: 10.1002/advs.202415321 (PMC11984905; doi:10.1002/advs.202415321)
Supplement: Supplementary file 1 — Supporting Information [file ADVS-12-2415321-s001.docx]

Supporting Information

Chiral Switch of Gadopiclenol: New Standards in MRI Probes

Roberta Napolitano,^a^ Nicol Guidolin,^b^ Mariangela Boccalon,^b^ Alberto Fringuello Mingo,^a^ Sonia Colombo Serra,^a^ Federica Buonsanti,^a^ Roberta Fretta,^a^ Nicola Demitri,^c^ Attila Bényei,^d^ Mauro Botta,^e^ Giovanni B. Giovenzana^[f^ Fabio Tedoldi,^g^,* Zsolt Baranyai^b^,*

^a^ Bracco Research Centre, Bracco Imaging SpA, Via Ribes 5, 10010 Colleretto Giacosa (TO),Italy

^b^ CRB Trieste, Bracco Imaging SpA, AREA Science Park, 34149 Basovizza (TS), Italy, email: [zsolt.baranyai@bracco.com](mailto:zsolt.baranyai@bracco.com)

^c^ XRD2 Beamline, Elettra−Sincrotrone Trieste S.C.p.A., 34149 Basovizza (TS), Italy

^d^ Department of Physical Chemistry, University of Debrecen, Egyetem tér 1, H–4010 Debrecen, Hungary

^e^ Dipartimento di Scienze e Innovazione Tecnologica, Piattaforma di Risonanze Magnetiche (PRISMA-UPO), Università del Piemonte Orientale "A. Avogadro", Viale T. Michel 11, 15121 Alessandria (AL), Italy

^f^ Dipartimento di Scienze del Farmaco (DSF), Università del Piemonte Orientale "A. Avogadro", Largo Donegani 2/3, 28100 Novara (NO), Italy

^g^ Headquarters, Bracco Imaging SpA, Via Egidio Folli 50, 20134 Milano, Italy, email: [fabio.tedoldi@bracco.com](mailto:fabio.tedoldi@bracco.com)

**Table of contents**

[**1. Synthesis** 3](#_Toc188523125)

[**1.1 Synthesis of [Gd(*rac*-L_1_)] and [Gd(*rac*-L_2_)]** 3](#_Toc188523126)

[*1.1.1 Synthesis of Compound 1 ((2S)-2-bromopentanedioic acid)* 3](#_Toc188523127)

[*1.1.2 Synthesis of Compound 2 (diethyl (2S)-2bromopentanedioate)* 3](#_Toc188523128)

[*1.1.3 Synthesis of Compound 3 (hexaethyl 2,2',2''-[3,6,9,15-tetraazabicyclo[9.3.1]pentadeca-1(15),11,13-triene-3,6,9-triyl]tripentanedioate)* 5](#_Toc188523129)

[*1.1.4 Synthesis of ligand H_6_rac-L_1_ (2,2’,2’’-[3,6,9,15-tetraazabicyclo[9.3.1]pentadeca-1(15),11,13-triene-3,6,9-triyl]tripentanedioic acid)* 5](#_Toc188523130)

[*1.1.5 Synthesis of [Gd(rac-L_1_)] (2,2’,2’’-[3,6,9,15-tetraazabicyclo[9.3.1]pentadeca-1(15),11,13-triene-3,6,9-triyl]tris(4-carboxybutanoate)gadolinium)* 7](#_Toc188523131)

[*1.1.6 Synthesis of [Gd(rac-L_2_)] ((α3, α6, α9)-tris(3-((2,3-dihydroxypropyl)amino)-3-oxopropyl)-3,6,9,15-tetraazabicyclo(9.3.1)pentadeca-1(15),11,13-triene-3,6,9-triacetato(3-)-(κN3,κN6,κN9,κN15,κO3,κO6,κO9)gadolinium)* 7](#_Toc188523132)

[**1.2 Isolation of [Gd(*ent*-L_1_)]by flash chromatography** 8](#_Toc188523133)

[**1.3 Synthesis of [Gd(*ent*-L_2_)]** 9](#_Toc188523134)

[**1.4 Stereoselective synthesis of [Gd(*RRR-ent*-L_1_)]** 10](#_Toc188523135)

[*1.4.1 Synthesis of Compound 4 (dimethyl (2S)-2-hydroxypentanedioate)* 10](#_Toc188523136)

[*1.4.2 Synthesis of Compound 6 (hexamethyl (2R),(2'R),(2''R)-2,2’,2’’-[3,6,9,15-tetraazabicyclo[9.3.1]pentadeca-1(15),11,13-triene-3,6,9-triyl]tripentanedioate)* 10](#_Toc188523137)

[*1.4.3 Synthesis of ligand H_6_RRR-ent-L_1_* 11](#_Toc188523138)

[*1.4.4 Synthesis of [Gd(RRR-ent-L_1_)]* 11](#_Toc188523139)

[**1.5 Stereoselective synthesis of [Gd(*SSS-ent*-L_1_)]** 11](#_Toc188523140)

[*1.5.1 Synthesis of Compound 7 (dimethyl (2R)-2-hydroxypentanedioate)* 12](#_Toc188523141)

[*1.5.2 Synthesis of Compound 9 (hexamethyl (2S),(2'S),(2''S)-2,2’,2’’-[3,6,9,15-tetraazabicyclo[9.3.1]pentadeca-1(15),11,13-triene-3,6,9-triyl]tripentanedioate)* 12](#_Toc188523142)

[*1.5.3 Synthesis of ligand H_6_SSS-ent-L_1_* 13](#_Toc188523143)

[*1.5.4 Synthesis of [Gd(SSS-ent-L_1_)]* 13](#_Toc188523144)

[**1.6. Preparation of ligands** 13](#_Toc188523145)

[*1.6.1 Synthesis of ent-L_1_ ligand* 13](#_Toc188523146)

[*1.6.2 Synthesis of ent-L_2_ ligand* 15](#_Toc188523147)

[**2. HPLC separation of the isomers formed by the Gd(III)-complexes** 17](#_Toc188523148)

[**3. X-ray diffraction studies** 20](#_Toc188523149)

[**3.1 Crystallization of the Cu(II)- and Gd(III)-complexes** 20](#_Toc188523150)

[**3.2 X-ray data analysis and refinement** 21](#_Toc188523151)

[**3.3 X-ray structure of [Cu(H_4_*rac*-L_1_)], [Gd(PCTA)(CO_3_)]^2-^, [Gd(H_3_*ent*-L_1_)(C_2_O_4_)]^2-^ and [Gd(*ent*-L_2_)(CO_3_)]^2-^** 21](#_Toc188523152)

[**4. Solution structure of the Y(III)-complexes with PCTA, *ent*-L_1_ and *ent*-L_2_ ligands** 36](#_Toc188523153)

[**5. Thermodynamic properties of the Ca(II)-, Zn(II)-, Cu(II)- and Gd(III)-complexes** 43](#_Toc188523154)

[**5.1 Acid-base properties of *rac*-L_1_, *ent*-L_1_, *rac*-L_2_ and *ent*-L_2_ ligands** 43](#_Toc188523155)

[**5.2 Complexation features of *rac*-L_1_, *ent*-L_1_, *rac*-L_2_ and *ent*-L_2_ ligands** 45](#_Toc188523156)

[**6. Kinetic inertness of the Gd(III)-complexes** 56](#_Toc188523157)

[**7. Relaxation properties of the Gd(III)-complexes** 60](#_Toc188523158)

[**8. References** 64](#_Toc188523159)

# **1. Synthesis**

## **1.1 Synthesis of [Gd(*rac*-L_1_)] and [Gd(*rac*-L_2_)]**

[Gd(*rac*-L_1_)] and [Gd(*rac*-L_2_)] were prepared by using the procedure reported in Ref. ^[52]^ and ^[53]^ applying small modifications, according to **Scheme S1**:

**Scheme S1.** Synthesis of [Gd(*rac*-L_1_)] and [Gd(*rac*-L_2_)]. * and ° represent the stereocenters

### *1.1.1 Synthesis of Compound 1 ((2S)-2-bromopentanedioic acid)*

L-glutamic acid (33.0 g, 0.224 mol) and sodium bromide (79.7 g, 0.782 mol) were suspended in 2 M HBr (225 mL). The suspension was cooled to -5 °C and sodium nitrite (28.0 g, 0.403 mol) was slowly added in small portions over 2.5 hours, maintaining the inner temperature lower than 0 °C. The yellow mixture was stirred for additional 20 minutes at -5 °C and then concentrated sulfuric acid (29 mL) was dropped. The dark brown mixture was warmed to RT and then extracted with diethyl ether (4 🞨150 mL). The combined organic phases were washed with brine, dried over Na_2_SO_4_ and concentrated under reduced pressure to a brown oil (21.2 g), that was used in the following step with no further purification.

### *1.1.2 Synthesis of Compound 2 (diethyl (2S)-2bromopentanedioate)*

Crude Compound 1 (21.2 g) was dissolved in ethanol (240 mL) and the resulting solution was cooled in ice. Thionyl chloride (14.5 mL, 0.199 mol) was slowly dropped, then the slightly yellow solution was stirred at RT for 2 days. Solvents were then removed under reduced pressure, the oily residue was dissolved in dichloromethane (200 mL) and washed with 5% NaHCO_3_ (4 x 100 mL), water (1 🞨 100 mL), and brine (1 🞨 100 mL). The resulting organic phase was concentrated *in vacuo* and the residue was purified by chromatography on silica, eluting with petrol ether-ethyl acetate 3:1, obtaining 19.5 g of pure product. (Yield 33%).

^1^H NMR (600 MHz, CDCl_3_, 298 K): δ 4.37 (dd, *J* = 8.5, 5.8 Hz, 1H), 4.26 (qd, *J* = 7.1, 3.1 Hz, 2H), 4.17 (q, *J* = 7.1 Hz, 2H), 2.59 – 2.47 (m, 2H), 2.45 – 2.36 (m, 1H), 2.35 – 2.24 (m, 1H), 1.32 (t, *J* = 7.1 Hz, 3H), 1.29 (t, *J* = 7.1 Hz, 3H), **Figure S1**. ^13^C NMR (151 MHz, CDCl_3_, 298 K): δ 172.09, 169.38, 62.14, 60.75, 45.06, 31.57, 30.35, 14.19, 13.94, **Figure S2**.


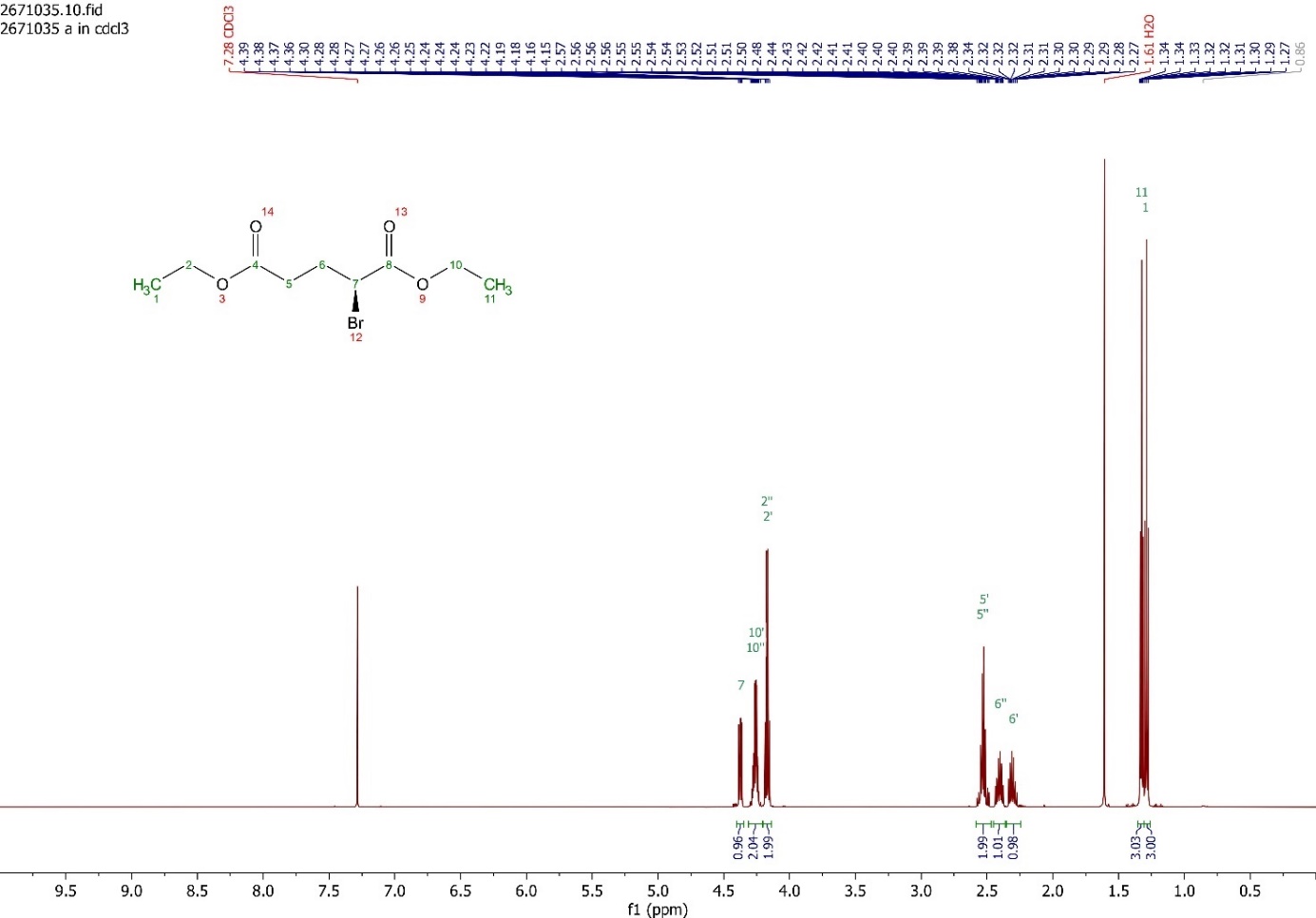


**Figure S1.** ^1^H NMR spectra of Compound 2 at 600 MHz and 298 K in CDCl_3_


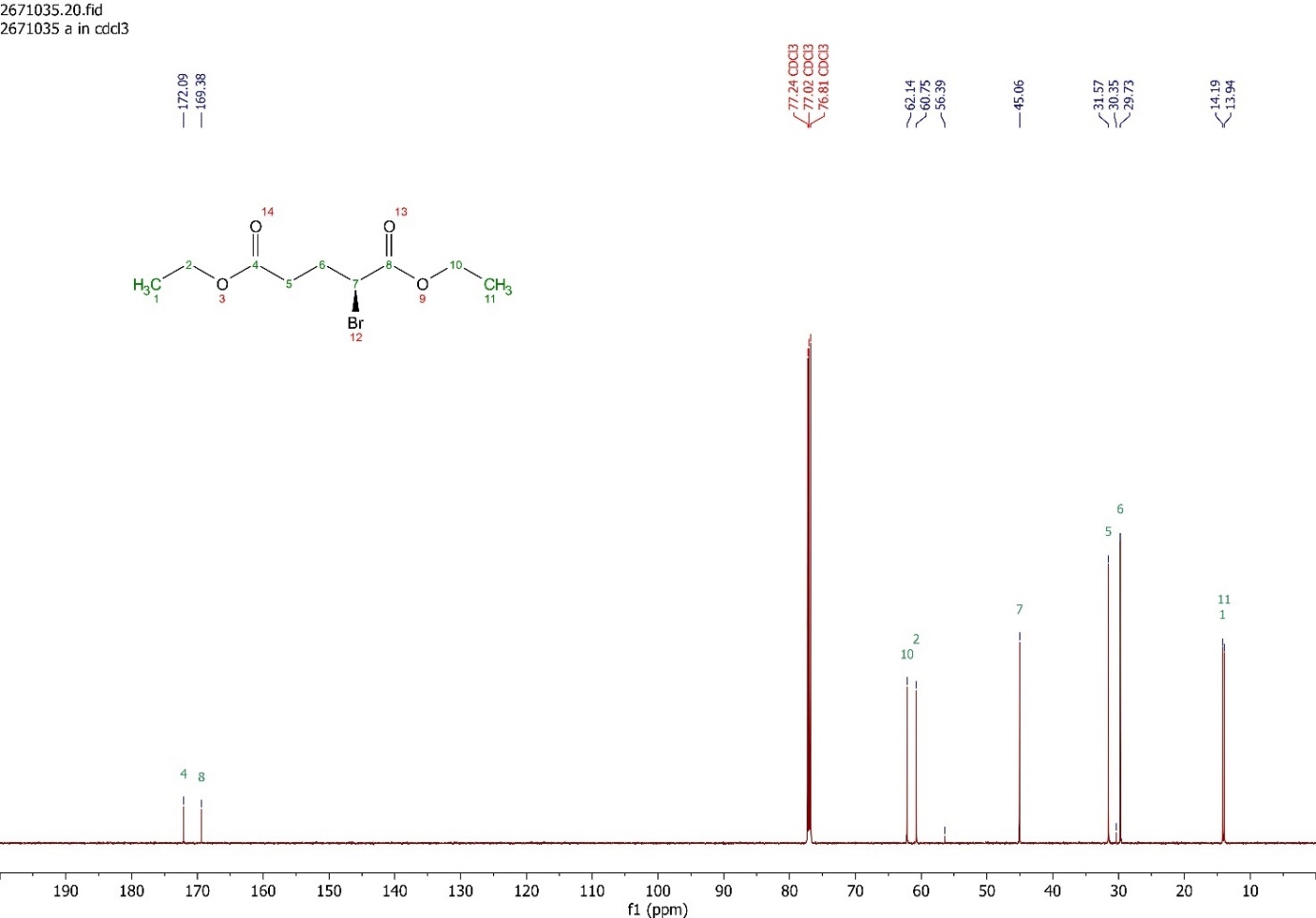


**Figure S2.** ^13^C NMR spectra of Compound 2 at 151 MHz and 298 K in CDCl_3_

### *1.1.3 Synthesis of Compound 3 (hexaethyl 2,2',2''-[3,6,9,15-tetraazabicyclo[9.3.1]pentadeca-1(15),11,13-triene-3,6,9-triyl]tripentanedioate)*

A solution of Compound 2 (17.2 g, 0.0645 mol) in acetonitrile (40 mL) was added to a suspension of Pyclen (3.80 g, 0.018 mol prepared as described in Ref. ^[54]^) and K_2_CO_3_ (11.2 g, 0.0808 mol) in acetonitrile (150 mL). The yellow suspension was heated at 65 °C for 24 hours, then salts were filtered off and the organic solution was concentrated *in vacuo*. The orange oil was dissolved in dichloromethane (200 mL) and the product was extracted in 1 M HCl (4🞨100 mL). The aqueous phases were combined, cooled in ice and pH was increased up to 8 with 30% NaOH. The desired compound was then extracted with dichloromethane (4🞨150 mL) and the combined organic phases were concentrated *in vacuo* obtaining a brown oil (10.1 g, yield 73%), that was used in the following step with no further purification. [M+H]^+^: 765.3

### *1.1.4 Synthesis of ligand H_6_rac-L_1_ (2,2’,2’’-[3,6,9,15-tetraazabicyclo[9.3.1]pentadeca-1(15),11,13-triene-3,6,9-triyl]tripentanedioic acid)*

Compound 3 (9.99 g, 0.013 mol) was dissolved in ethanol (40 mL) and 5 M NaOH (40 mL). The brown solution was heated at 80 °C for 23 hours. Ethanol was then concentrated *in vacuo*, the solution was cooled in ice and brought to pH 2 with conc. HCl solution. The ligand was purified on XAD 1600 eluting with water-acetonitrile gradient, obtaining after freeze-drying 5.7 g as white solid (yield 73%). The product was characterized in HPLC (**Table S1**), obtaining three peaks with retention time: *r*_t_=15.9, 17.0 and 17.2 min. [M+H]^+^: 597.2

^1^H NMR (600 MHz, D_2_O, 338 K) δ 8.28 (t, *J* = 7.8 Hz, 1H), 7.78 (d, 2H), 5.12 – 4.76 (m, 4H), 4.27 – 4.11 (m, 2H), 4.08 – 3.98 (m, 1H), 3.97 – 3.72 (m, 4H), 3.70 – 3.26 (m, 4H), 3.04 – 2.80 (m, 6H), 2.61 – 2.22 (m, 6H), **Figure S3**. ^13^C NMR (151 MHz, D_2_O, 338 K) δ 177.09, 176.90, 174.14, 172.58, 152.63, 140.80, 123.22, 67.41, 61.72, 55.45, 51.82, 48.60, 31.27, 23.02, 20.17, **Figure S4**.


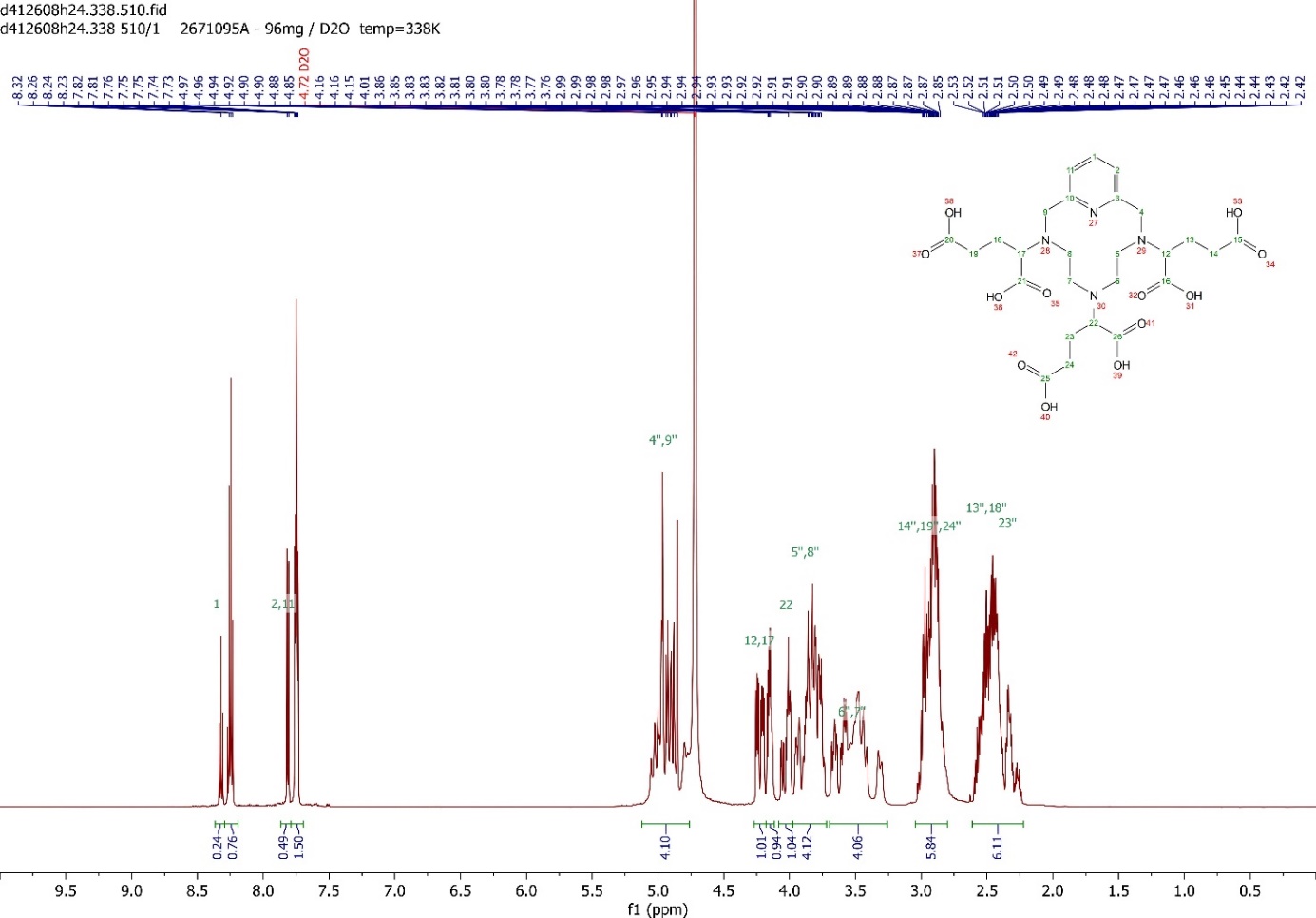


**Figure S3.** ^1^H NMR spectra of ligand *rac*-L_1_ at 600 MHz and 338 K in D_2_O


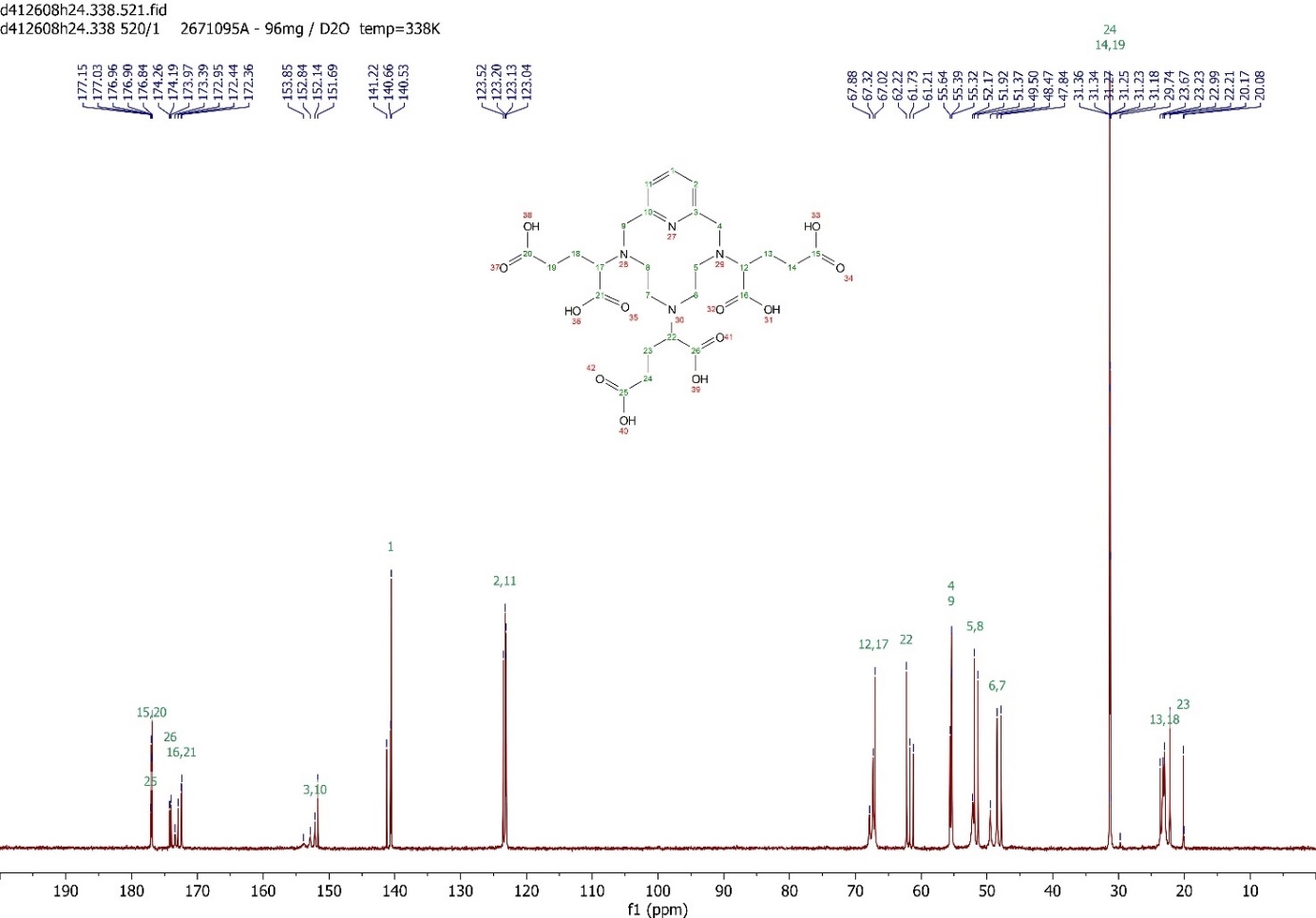


**Figure S4.** ^13^C NMR spectra of ligand *rac*-L_1_ at 151 MHz and 338 K in D_2_O

### *1.1.5 Synthesis of [Gd(rac-L_1_)] (2,2’,2’’-[3,6,9,15-tetraazabicyclo[9.3.1]pentadeca-1(15),11,13-triene-3,6,9-triyl]tris(4-carboxybutanoate)gadolinium)*

Ligand *rac*-L_1_ (5.25 g, 0.0088 mol) was dissolved in milliQ water (100 mL) and the solution was brought to pH 7 with 2 M NaOH (20 mL). Aqueous solution of GdCl_3_ (0.0088 mol) was slowly added at RT, maintaining pH at 7 with 2 M NaOH. Once the complexation was completed (checked by HPLC-MS), the solution was concentrated *in vacuo* and purified on XAD 1600 eluting with water-acetonitrile gradient, in order to remove salts and impurities. After freeze-drying the pure compound was obtained as white solid (6.79 g, yield 94%). The product was characterized in HPLC (Table S1), obtaining four peaks with retention time: *r*_t_=16 ÷ 22 min (**Figure S5**), [M+H]^+^: 752.0

### *1.1.6 Synthesis of [Gd(rac-L_2_)] ((α3, α6, α9)-tris(3-((2,3-dihydroxypropyl)amino)-3-oxopropyl)-3,6,9,15-tetraazabicyclo(9.3.1)pentadeca-1(15),11,13-triene-3,6,9-triacetato(3-)-(κN3,κN6,κN9,κN15,κO3,κO6,κO9)gadolinium)*

[Gd(*rac*-L_1_)] (0.90 g, 0.0011 mol) was added to a solution of racemic isoserinol (0.40 g, 0.0044 mol) in water (15 mL) adjusted to pH 6 with conc. HCl solution. Then, EDCI·HCl (1.0 g, 0.0055 mol) and HOBT (0.12 g, 0.00088 mol) were added and the solution was stirred at pH 6 and RT for 24 hours. The product was then purified by flash chromatography on silica-C_18_ column, eluting with a water-acetonitrile gradient. Fractions containing the pure compound were concentrated and freeze-dried, obtaining a white solid (0.83 g, yield 78%). The product was characterized in HPLC (**Table S2**), obtaining four peaks with retention time: *r*_t_= 24 ÷ 27 min (**Figure S14-I**). [M+H]^+^: 971.1

## **1.2 Isolation of [Gd(*ent*-L_1_)]by flash chromatography**

[Gd(*rac*-L_1_)] was obtained as a mixture of 8 diastereomers (four pairs of enantiomers) separated by HPLC (Figure S5).


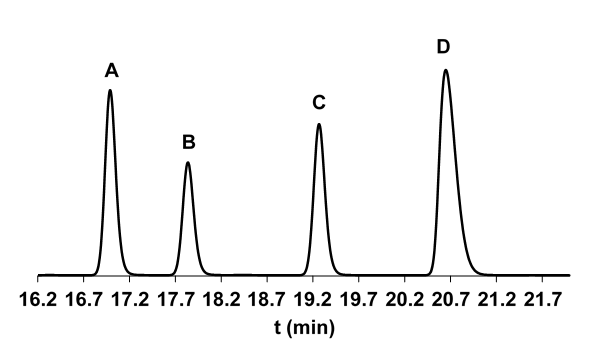


**Figure S5.** HPLC chromatogram of [Gd(r*ac*-L_1_)].

(HPLC area at 254 nm: A=24%, B=15%, C=21%, D=40%)

It was possible to isolate *RRR/SSS* enantiomers of [Gd(*rac*-L_1_)], named [Gd(*ent*-L_1_)] by reverse phase chromatography (**Figure S6**).

**
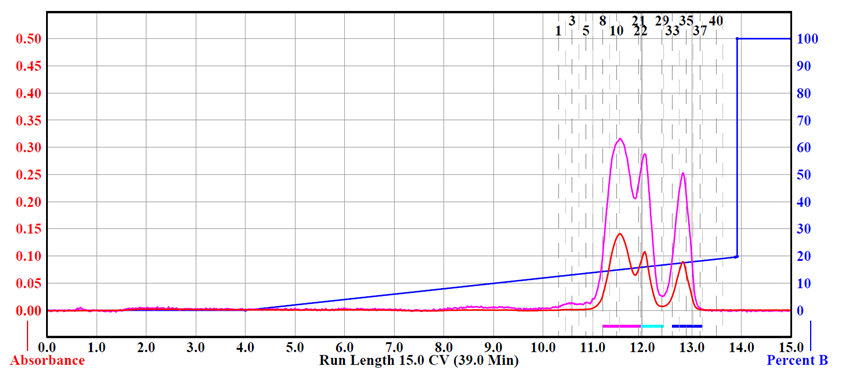
**

**Figure S6.** Flash chromatographic purification of [Gd(*rac*-L_1_)]

[Gd(*rac*-L_1_)] (1.0 g, 0.0013 mol) was dissolved in water (4 mL) and the solution was acidified to pH 2 with conc. HCl solution. The obtained solution was loaded into a pre-packed column of silica-C_18_ (Biotage® SNAP ULTRA C18 120 g, HP-sphere C18 25 μm) and purified with an automated flash chromatography system eluting with deionized water (4 CV) and then a very slow gradient of acetonitrile (for example from 0 to 20% of acetonitrile in 10 CV).

Fractions related to [Gd(*ent*-L_1_)] (28-38, eluted as the last peak with ca. 17-18% of acetonitrile) were combined, concentrated under reduced pressure and freeze-dried, obtaining a white solid (170 mg, HPLC area of peak C at 254 nm: 98.8%, **Figure S7**). [M+H]^+^: 752.0

**Figure S7.** HPLC chromatogram of [Gd(*ent*-L_1_)] obtained by flash chromatography

(HPLC area at 254 nm: A=0%, B=0.6%, C=98.8%, D=0.6%)

.

All the other fractions (6-27) were combined together and freeze-dried obtaining 583 mg of a white solid enriched of peaks A (28.3%), B (27.4%) and D (41.3%).

## **1.3 Synthesis of [Gd(*ent*-L_2_)]**

[Gd(*ent*-L_1_)] (54 mg, titer 90%, 0.065 mmol) was dissolved in milliQ water (5 mL), and racemic isoserinol (27 mg, 0.29 mmol) was added adjusting the pH at 6 with 1 M HCl. Then, EDCI·HCl (62 mg, 0.32 mmol) and HOBT (4.3 mg, 0.032 mmol) were added and the solution was stirred at RT at pH 6 for 24 hours. The solution was concentrated and loaded into a pre-packed silica-C_18_ column (Biotage® SNAP ULTRA C18 12 g, HP-sphere C18 25 μm), eluting with water-acetonitrile gradient using an automated flash chromatography system. Fractions containing the pure product, one major peak at the HPLC (Table S2), were combined, concentrated and freeze-dried giving a white solid (60 mg, yield 95%, Figure S14-IV). [M+H]^+^: 971.1

## **1.4 Stereoselective synthesis of [Gd(*RRR-ent*-L_1_)]**

[Gd(*RRR*-*ent*-L_1_)] complex was prepared according to **Scheme S2**:

**Scheme S2.** Synthesis of [Gd(*RRR-ent*-L_1_)].

### *1.4.1 Synthesis of Compound 4 (dimethyl (2S)-2-hydroxypentanedioate)*

37% aq. HCl (50 μL) was added to a solution of (S)-(+)-5-oxotetrahydrofuran-2-carboxylic acid (2.48 g, 0.019 mol) in anhydrous methanol (20 mL). The solution was refluxed under N_2_ atmosphere for 24 h. After cooling in ice, NaHCO_3_ was added, the suspension was filtered, concentrated and purified on silica gel with hexanes/ethyl acetate 1:1. Fractions containing the pure product were combined and concentrated, giving a colorless oil (2.97 g, yield 89%).

### *1.4.2 Synthesis of Compound 6 (hexamethyl (2R),(2'R),(2''R)-2,2’,2’’-[3,6,9,15-tetraazabicyclo[9.3.1]pentadeca-1(15),11,13-triene-3,6,9-triyl]tripentanedioate)*

Compound 4 (445 mg, 2.52 mmol) was dissolved in anhydrous dichloromethane (6 mL) and triethylamine (0.87 mL, 6.31 mmol) was added. The solution was cooled at –40 °C and then trifluoromethanesulfonic anhydride (0.49 mL, 2.91 mmol) was slowly added. The dark solution was stirred at –40 °C for 1 h, then a solution of Pyclen (104 mg, 0.506 mmol) in anhydrous dichloromethane (3 mL) and triethylamine (1 mL, 7.56 mmol) were added and the solution was slowly brought to RT and stirred at RT overnight. The organic solution was then washed with 2 M HCl (4 × 10 mL), the aqueous phase was extracted again with dichloromethane (3 × 10 mL). The organic phases were combined and concentrated under reduced pressure, obtaining 400 mg of a brown oil that was used in the following step with no further purification.

### *1.4.3 Synthesis of ligand H_6_RRR-ent-L_1_*

Crude Compound 6 (400 mg) was dissolved in methanol (2.5 mL) and 5 M NaOH (2.5 mL). The brown solution was heated at 80 °C for 22 h to ensure complete hydrolysis. Methanol was concentrated, the solution was brought to pH 1 with concentrated HCl and purified through an automated flash chromatography system with a silica-C_18_ pre-packed column (Biotage® SNAP ULTRA C_18_ 12 g, HP-sphere C18 25 μm), eluting with water/acetonitrile gradient. Fractions containing the pure product were combined, concentrated under reduced pressure and freeze-dried (64 mg, yield 18 %). The HPLC (Table S1) showed a major peak.

### *1.4.4 Synthesis of [Gd(RRR-ent-L_1_)]*

Ligand *RRR* H_6_*ent*-L_1_ (32 mg, 0.054 mmol) was dissolved in deionized water (4 mL) and the pH was adjusted to 7 with 1 M NaOH. GdCl_3_·6H_2_O (20 mg, 0.054 mmol) was added and the pH was adjusted to 7 with 0.1 M NaOH. The clear solution was stirred at RT overnight and the end of the complexation was checked by xylenol orange and HPLC (Table S1), showing the desired *RRR* isomer as major peak, about 80% in area %. The mixture was brought to pH 2 with concentrated HCl and purified through an automated flash chromatography system with a silica-C_18_ pre-packed column (Biotage® SNAP ULTRA C18 12 g, HP-sphere C_18_ 25 μm), eluting with deionized water/acetonitrile gradient. Fractions containing the pure product were combined, concentrated under reduced pressure and freeze-dried (36 mg, yield 90%). See **Figure S12-III**. and **Figure S13-II**.

## **1.5 Stereoselective synthesis of [Gd(*SSS-ent*-L_1_)]**

*SSS* enriched [Gd(*ent*-L_1_)] complex was prepared according to **Scheme S3**:

**Scheme S3.** Synthesis of [Gd(*SSS-ent*-L_1_)].

### *1.5.1 Synthesis of Compound 7 (dimethyl (2R)-2-hydroxypentanedioate)*

37% aq. HCl (100 μL) was added to a solution of (R)-(-)-5-oxotetrahydrofuran-2-carboxylic acid (5.0 g, 0.038 mol) in anhydrous methanol (45 mL). The solution was refluxed under N_2_ atmosphere for 24 h. After cooling in ice, NaHCO_3_ was added, the suspension was filtered, concentrated under reduced pressure and purified on silica gel with hexanes/ethyl acetate 1:1. Fractions containing the pure product were combined and concentrated, giving a colorless oil (6.7 g, yield 99%).

### *1.5.2 Synthesis of Compound 9 (hexamethyl (2S),(2'S),(2''S)-2,2’,2’’-[3,6,9,15-tetraazabicyclo[9.3.1]pentadeca-1(15),11,13-triene-3,6,9-triyl]tripentanedioate)*

Compound 7 (470 mg, 2.67 mmol) was dissolved in anhydrous dichloromethane (6 mL) and trimethylamine (0.93 mL, 6.67 mmol) was added. The solution was cooled down at –40 °C and then trifluoromethanesulfonic anhydride (0.50 mL, 3.07 mmol) was slowly dropped. The dark solution was stirred at –40 °C for 1 h, then Pyclen (140 mg, 0.679 mmol) and trimethylamine (0.93 mL, 6.67 mmol) were added and the solution was slowly brought to RT overnight. The organic solution was then washed with water (3 × 5 mL) and 2 M HCl (4 × 5 mL). The aqueous phase was extracted again with dichloromethane (3 × 10 mL). the organic phases were combined and concentrated under reduced pressure, obtaining 350 mg of a brown oil that was used in the following step with no further purification.

### *1.5.3 Synthesis of ligand H_6_SSS-ent-L_1_*

Crude Compound 9 (350 mg) was dissolved in methanol (4.5 mL) and 5 M NaOH (4.5 mL). The brown solution was heated at 80 °C for 16 h to ensure complete hydrolysis. Methanol was concentrated, the solution was brought to pH 2 with concentrated HCl and purified through an automated flash chromatography system with a silica-C_18_ pre-packed column (Biotage® SNAP ULTRA C_18_ 12 g, HP-sphere C18 25 μm), eluting with water/acetonitrile gradient. Fractions containing the pure product were combined, concentrated under reduced pressure and freeze-dried (52 mg, yield 17 %). The HPLC (Table S1) showed a major peak.

### *1.5.4 Synthesis of [Gd(SSS-ent-L_1_)]*

Ligand *SSS* H_6_*ent*-L_1_ (34 mg, 0.057 mmol) was dissolved in deionized water (5 mL) and the pH was adjusted to 7 with 1 M NaOH. GdCl_3_·6H_2_O (20 mg, 0.054 mmol) was added and the pH was adjusted to 7 with 0.1 M NaOH. The clear solution was stirred at RT overnight and the end of the complexation was checked by xylenol orange and HPLC (Table S1), showing the desired *SSS* isomer as major peak, about 85% in area %. The mixture was brought to pH 2.5 with concentrated HCl and purified through an automated flash chromatography system with a silica-C_18_ pre-packed column (Biotage® SNAP ULTRA C18 12 g, HP-sphere C_18_ 25 μm), eluting with deionized water/acetonitrile gradient. Fractions containing the pure product were combined, concentrated under reduced pressure and freeze-dried (39 mg, yield 87%). See Figure S12-IV. and Figure S13-III.

## **1.6. Preparation of ligands**

### *1.6.1 Synthesis of ent-L_1_ ligand*

*Ent*-L_1_ ligand was prepared according to **Scheme S4**. A suspension of [Gd(*ent*-L_1_)] (2.49 g, 0.0033 mol) in water (100 mL) was heated at 90 °C obtaining clear solution. Then oxalic acid dihydrate (1.88 g, 0.0149 mol) was added and the solution was heated at 90 °C for 2 hours observing the formation of a white precipitate. The suspension was cooled at RT, the solid was filtered off and the aqueous solution was concentrated *in vacuo* up to 10 mL and purified by flash chromatography on a pre-packed silica-C_18_ cartridge (Teledyne® Redisept Gold C_18_ Aq 415 g) with a water-acetonitrile gradient (4 CV with water, from 0 to 20% of acetonitrile in 20 CV). Fractions containing the desired isomer (eluted with 12% of acetonitrile) were combined, concentrated under reduced pressure and freeze-dried obtaining pure *ent*-L_1_ ligand as a white solid (0.956 g, yield 48%). HPLC (Table S1): *r*_t_=17 min. [M+H]^+^: 597.2

^1^H NMR (600 MHz, D_2_O, 338 K): δ 8.35 (td, *J* = 7.8, 1.1 Hz, 1H), 7.85 (d, *J* = 7.8 Hz, 2H), 5.11 – 4.93 (m, 4H), 4.25 (dd, J = 8.8, 5.1 Hz, 2H), 4.14 (dd, *J* = 10.4, 3.3 Hz, 1H), 4.01 – 3.87 (m, 4H), 3.70 – 3.47 (m, 4H), 3.10 – 2.92 (m, 6H), 2.66 – 2.52 (m, 4H), 2.50 (dd, *J* = 16.6, 7.2 Hz, 2H), **Figure S8**. ^13^C NMR (151 MHz, D_2_O, 338 K): δ 174.28, 173.00, 152.89, 140.74, 123.14, 68.01, 61.36, 55.52, 52.37, 48.53, 31.42, 23.08, 20.32, **Figure S9**.

**Scheme S4.** Synthesis of *ent*-L_1_ ligand. * represents the stereocenters.


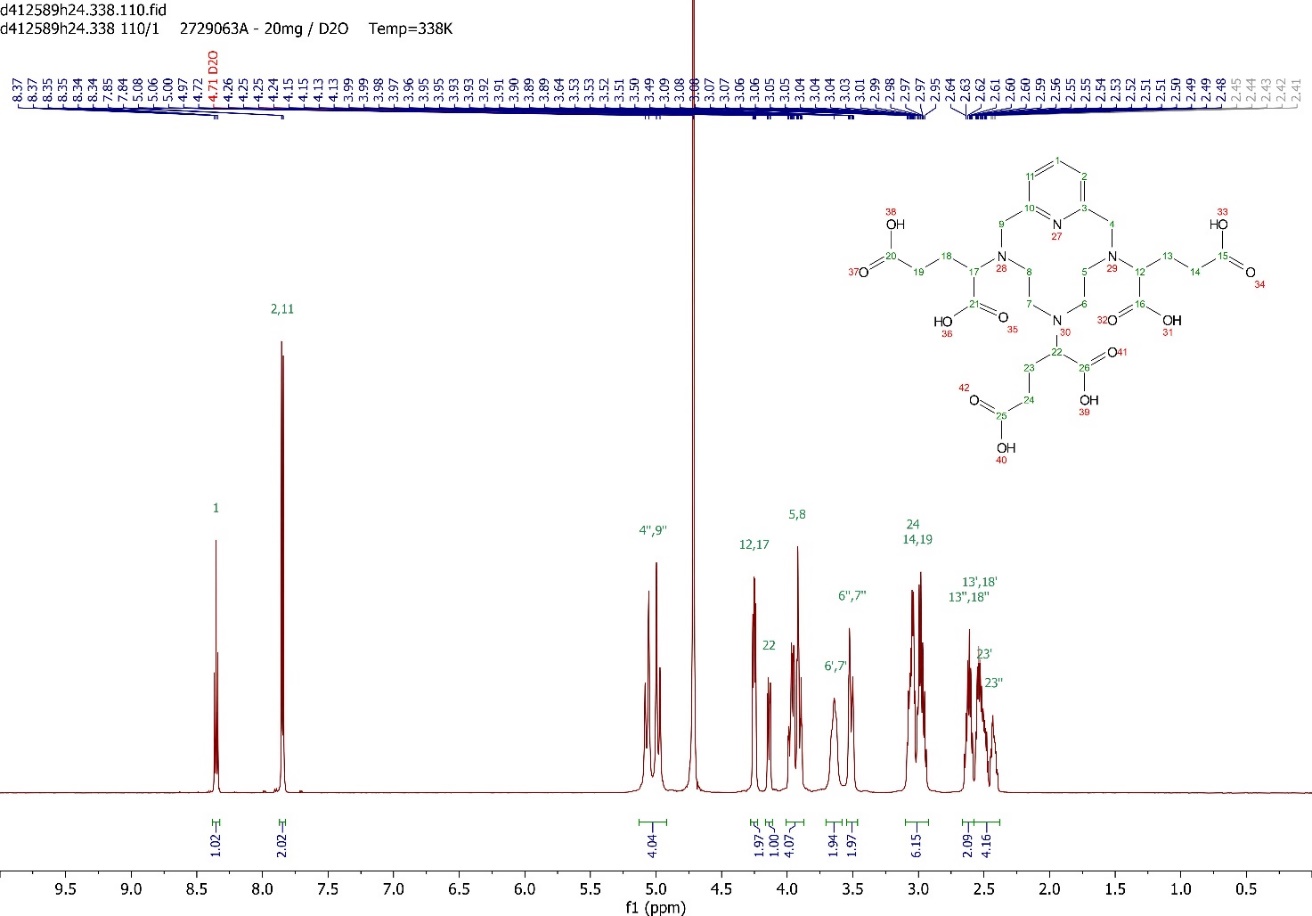


**Figure S8.** ^1^H NMR spectra of *ent*-L_1_ ligand at 600 MHz and 338 K in D_2_O


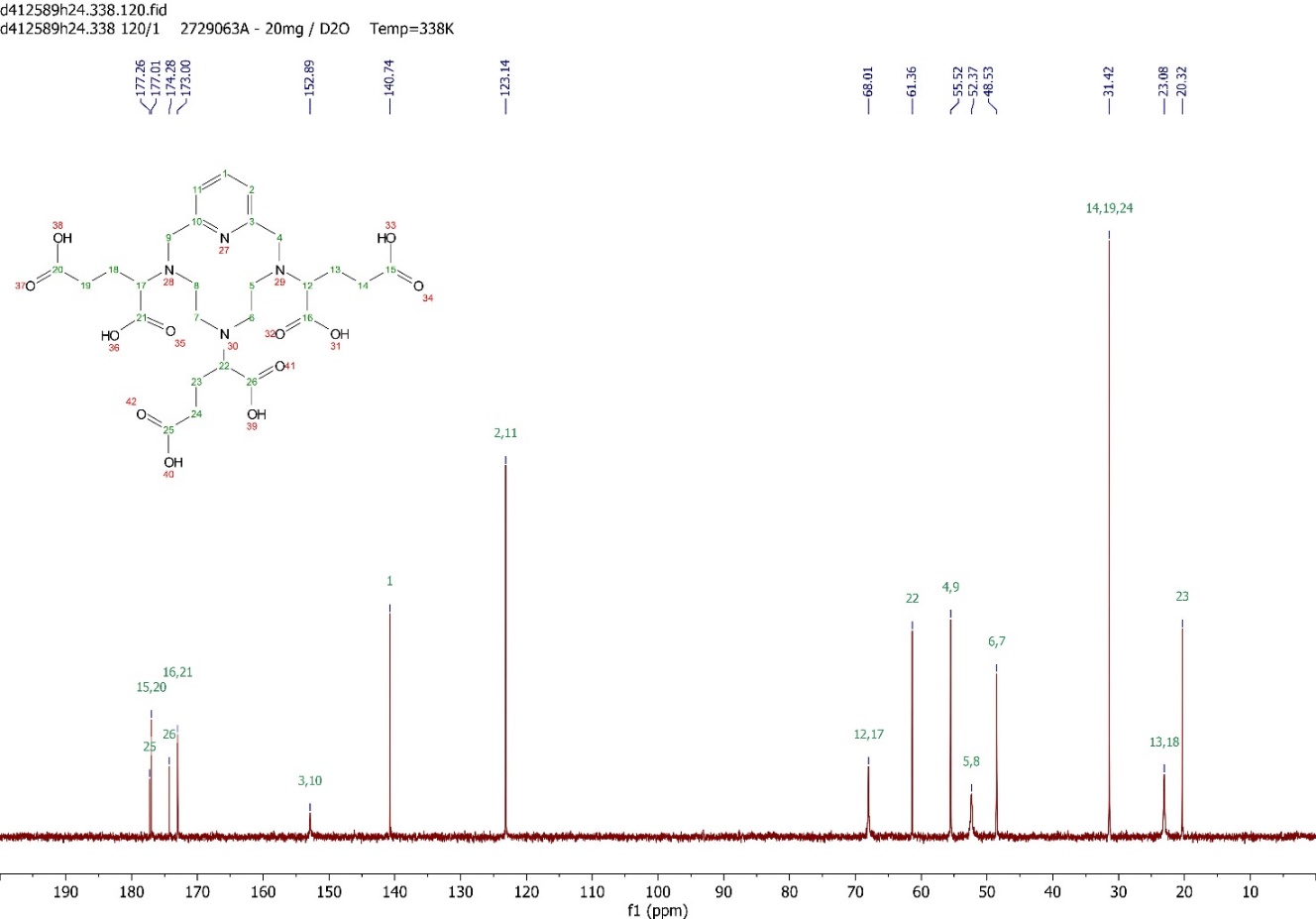


**Figure S9.** ^13^C NMR spectra of *ent*-L_1_ ligand at 151 MHz and 338 K in D_2_O

### *1.6.2 Synthesis of ent-L_2_ ligand*

*Ent*-L_2_ ligand was prepared according to **Scheme S5**. [Gd(*ent*-L_2_)] (6.1 g, 0.0629 mol) was dissolved in water (100 mL), then oxalic acid dihydrate (2.38 g, 0.0189 mol) was added and the solution was heated at 90 °C for 2 hours, observing the formation of a white precipitate. The suspension was cooled at RT, the solid was filtered off and the aqueous solution was concentrated under reduced pressure up to 20 mL and purified by flash chromatography on a pre-packed silica-C_18_ cartridge (Teledyne® Redisept Gold C_18_ Aq 415 g) with a water-acetonitrile gradient (4 CV with water, from 0 to 10% of acetonitrile in 15 CV). Fractions containing the desired isomer (eluted with 8% of acetonitrile) were combined, concentrated under reduced pressure and freeze-dried obtaining pure *ent*-L_2_ ligand as a white solid (2.49 g, yield 48%). HPLC (Table S2): *r*_t_= 23.6 min. [M+H]+: 816.4.

^1^H NMR (600 MHz, D_2_O, 338 K): δ 8.34 (t, *J* = 7.8 Hz, 1H), 7.83 (d, *J* = 7.8 Hz, 2H), 5.10 – 4.86 (m, 4H), 4.16 (dddd, *J* = 23.2, 8.1, 6.1, 3.3 Hz, 5H), 4.06 (dd, *J* = 10.2, 3.4 Hz, 1H), 4.01 – 3.84 (m, 11H), 3.77 – 3.52 (m, 11H), 3.00 – 2.78 (m, 6H), 2.65 – 2.40 (m, 7H), **Figure S10**. ^13^C NMR (151 MHz, D_2_O, 338 K): δ 175.53, 175.31, 174.17, 173.25, 153.53, 140.56, 122.97, 70.79, 68.46, 63.92 (d, J = 8.3 Hz), 61.49, 55.33, 52.34, 48.83, 42.39 (d, J = 4.0 Hz), 33.22, 32.97, 23.75, 20.93, **Figure S11**.

**Scheme S5.** Synthesis of *ent*-L_2_ ligand. * and ° represent the stereocenters

 
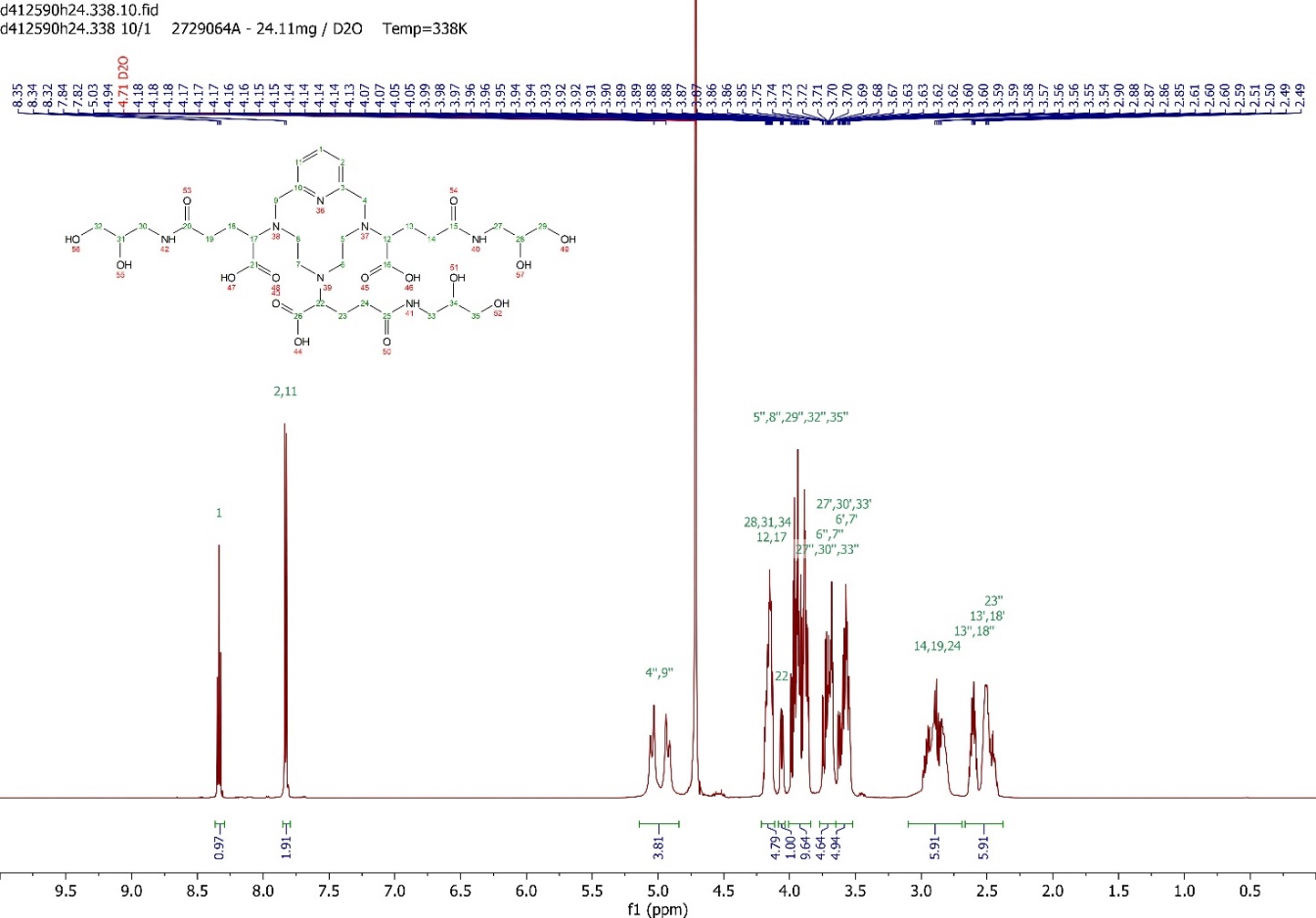


**Figure S10.** ^1^H NMR spectra of *ent*-L_2_ ligand at 600 MHz and 338 K in D_2_O


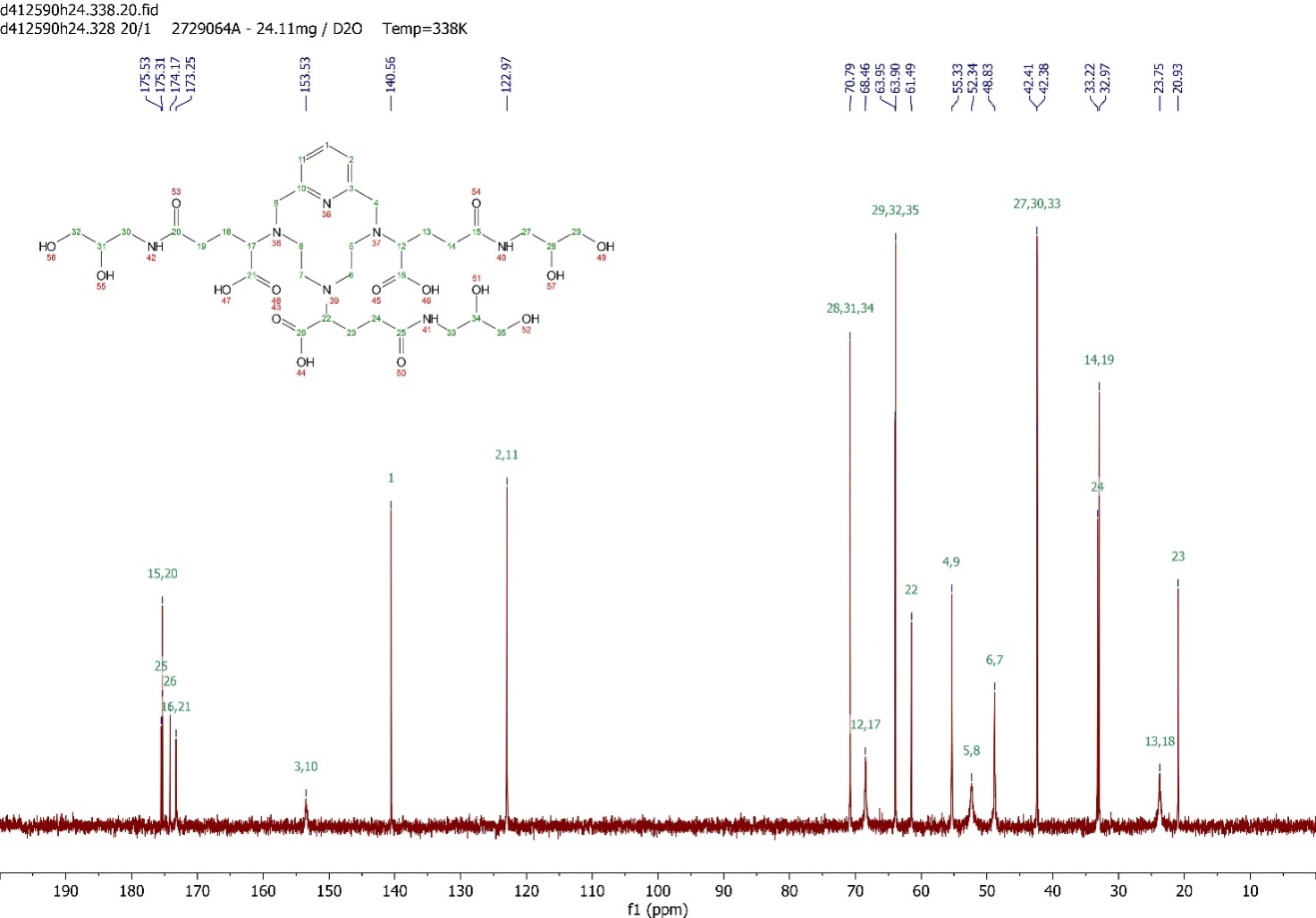
**Figure S11.** ^13^C NMR spectra of *ent*-L_2_ ligand at 151 MHz and 338 K in D_2_O

# **2. HPLC separation of the isomers formed by the Gd(III)-complexes**

Configuration isomers formed by Gd(III)-complexes with *rac*-L_1_ and *rac*-L_2_ ligands were investigated by HPLC. The experimental setup of the HPLC measurements is summarized in Tables S1 and S2.

**Table S1.** Experimental setup of the HPLC measurements for Gd(III)-complex of *rac*-L_1_

| **HPLC system** | HPLC equipped with quaternary pump, degasser, autosampler, PDA detector (*Agilent 1260 Infinity II*) | | |
| --- | --- | --- | --- |
| **Stationary phase** | Phenomenex Gemini 5 μm C18-110Å | | |
| **Mobile phase** | H_2_O/HCOOH 0.1 % : Methanol | | |
| **Elution: Gradient** | Time (min) | H_2_O/HCOOH 0.1% | Methanol |
|  | 0 | 95 | 5 |
|  | 5 | 95 | 5 |
|  | 30 | 50 | 50 |
|  | 35 | 50 | 50 |
|  | 40 | 95 | 5 |
| **Flow** | 0.6 mL/min | | |
| **Temperature** | 25 °C | | |
| **Detection** | PDA scan wavelenght 190-800nm | | |
| **Injection volume** | 50 µL | | |
| **Sample concentration** | 0.2 mM Gd(III) complex | | |
| **Stop time** | 40 min | | |
| **Retention time** | GdL ≅ 18-21 min; | | |

**Table S2.** Experimental setup of the HPLC measurements for Gd(III)-complex with *rac*-L_2_

| **HPLC system** | HPLC equipped with quaternary pump, degasser, autosampler, PDA and MS detector (*LCQ Deca XP-Plus – Thermo - Finnigan*) | | |
| --- | --- | --- | --- |
| **Stationary phase** | Phenomenex Gemini 5 μm C18-110Å | | |
| **Mobile phase** | H_2_O/TFA 0.1 % : Acetonitrile/TFA 0.1 % | | |
| **Elution: Gradient** | Time (min) | H_2_O/TFA 0.1% | Acetonitrile/TFA 0.1% |
|  | 0 | 100 | 0 |
|  | 5 | 100 | 0 |
|  | 22 | 90 | 10 |
|  | 26 | 90 | 10 |
| **Flow** | 0.5 mL/min | | |
| **Temperature** | 25 °C | | |
| **Detection** | PDA scan wavelenght 190-800nm  MS positive mode – Mass range 100 - 2000 | | |
| **Injection volume** | 50 µL | | |
| **Sample concentration** | 0.2 mM Gd(III) complex | | |
| **Stop time** | 26 min | | |
| **Retention time** | GdL ≅ 20 - 22 min; | | |

Chiral HPLC method was set up to separate the *RRR* and *SSS* enantiomers of Gd(III)-complex with *ent*-L_1_. The experimental setup of the chiral HPLC measurements is summarized in **Table S3**.

**Table S3.** Experimental setup of the chiral HPLC measurements for [Gd(*ent*-L_1_)] complex

| **HPLC system** | HPLC equipped with quaternary pump, degasser, autosampler, PDA detector (*Agilent 1200 or Waters Alliance 2695*) |
| --- | --- |
| **Stationary phase** | SUPELCO Astec CHIROBIOTIC 5 μm 4.6🞨250 mm |
| **Mobile phase** | H_2_O/HCOOH 0.025 % : Acetonitrile |
| **Elution: Isocratic** | 2 % Acetonitrile for 30 min |
| **Flow** | 1.0 mL/min |
| **Temperature** | 40 °C |
| **Detection** | PDA scan wavelenght 210 – 270 nm |
| **Stop time** | 30 min |
| **Retention time** | GdL ≅ 6-10 min; |

**Figure S12.** HPLC chromatogram of Gd(III)-complexes with *rac*-L_1_ ligand (**I.**), *ent*-L_1_ (**II.**), *RRR - ent*-L_1_ (**III.**) and *SSS - ent*-L_1_ (**IV.**) ligands.

**Figure S13.** Chiral HPLC chromatogram of Gd(III)-complexes with *ent*-L_1_ (**I.**), *RRR-ent*-L_1_ (**II.**) and *SSS-ent*-L_1_ (**III.**) ligands obtained by HPLC method in Table S3

**Figure S14.** HPLC chromatograms of Gd(III)-complexes with *rac*-L_2_ ligand (**I.**) and *ent*-L_2_ ligands conjugated to *R* (**II.**), *S* (**III.**) and racemic isoserinol (**IV.**) obtained by HPLC method in Table S2

# **3. X-ray diffraction studies**

## **3.1 Crystallization of the Cu(II)- and Gd(III)-complexes**

The tetraprotonated [Cu(H_4_*rac*-L_1_)] complex was prepared by reaction of equimolar H_6_*rac*-L_1_ with CuCO_3_ ⋅ Cu(OH)_2_ at pH=2.5 in H_2_O. The aqueous solution of the neutral tetraprotonated [Cu(H_4_*rac*-L_1_)] complex was boiled for 15 min in order to remove the CO_2_ formed in the reaction mixture. The aqueous solution of the tetraprotonated [Cu(H_4_*rac*-L_1_)] complex was kept at 4 °C in the crystallization plate. Slow evaporation of water results in the formation of two different single crystals characterized by “fiber” and “cubic” shape (**Figure S15**).

**A B**

**Figure S15.** “fiber” (**A**) and “cubic” (**B**) single crystals formed by the tetraprotonated [Cu(H_4_*rac*-L_1_)] complex.

Single crystals with the formulas [Cu(C_26_H_34_N_4_O_12_)] ⋅ 4H_2_O (“fiber”) and [Cu(C_26_H_34_N_4_O_12_)] ⋅ H_2_O (“cubic”) were isolated by manual separation. “Fiber” and “cubic” single crystals were found to be suitable for X-ray diffraction studies to determine the molecular structure and the configurations of the stereocenters in the “fiber” and “cubic” single crystals of the tetraprotonated [Cu(H_4_*rac*-L_1_)] complex.

In literature the X-ray structure of the hydrophilic Ln(III)-complexes with heptadentate ligand is very rare, which is probably related to the difficulties in the preparation of suitable single crystals. It had been evidenced that the hydrophilic Ln(III) complexes with heptadentate ligand can be crystallized by the substitution of the inner-sphere water molecules with bidentate ligands like CO_3_^2-^.^[55,56]^ In fact, single crystals of {(C(NH_2_)_3_)_2_[Gd(PCTA)(CO_3_)]}·4H_2_O have been obtained by slow diffusion of EtOH and Et_2_O mixture to aqueous solution of [Gd(PCTA)] (prepared by mixing equimolar Gd(OH)_3_ and H_3_PCTA) at pH=10.5 in the presence of 10 fold guanidine-carbonate excess. The pH of [Gd(PCTA)] solution was adjusted to 10.5 by addition of 10 fold guanidine-carbonate excess.

Single crystals of the triprotonated [Gd(H_3_*ent*-L_1_)] with the formula {(C(NH_2_)_3_)_2_[Gd(C_26_H_34_N_4_O_12_)(C_2_O_4_)]}·5H_2_O suitable for X-ray diffraction studies were obtained by slow evaporation of water from concentrated solution of triprotonated [Gd(H_3_*ent*-L_1_)] in the presence of 10 fold guanidine-oxalate ((C(NH_2_)_3_)_2_(C_2_O_4_)) excess. The pH of the [Gd(H_3_*ent*-L_1_)] − (C(NH_2_)_3_)_2_(C_2_O_4_) aqueous solution was adjusted to 3.3 by stepwise addition of solid oxalic acid.

Single crystals of [Gd(*ent*-L_2_)] complex with the formula {(C(NH_2_)_3_)_2_[Gd(C_35_H_54_N_7_O_15_)(CO_3_)]}·22H_2_O suitable for X-ray diffraction studies have been obtained with the slow diffusion of EtOH and Et_2_O mixture to aqueous solution of [Gd(*ent*-L_2_)] at pH=10.5 in the presence of 10 fold guanidine-carbonate excess. The pH of [Gd(*ent*-L_2_)] solution was adjusted to 10.5 by addition of 10 fold guanidine-carbonate excess.

## **3.2 X-ray data analysis and refinement**

Structures were solved by the dual space algorithm implemented in the SHELXT^[57]^ and SIR-92^[58]^ code. Fourier analysis and refinement were performed by the full-matrix least-squares methods based on F^2^ implemented in SHELXL (Version 2018/3).^[59]^ The Coot program was used for modeling.^[60]^ Thermal motion have been refined anisotropically excluding heavily disordered fragments and solvent molecules with occupancies smaller than 50%. Geometry and thermal motion parameters restrains (DFIX, DANG, SIMU and DELU) have been used on disordered fragments with poor electron densities. Hydrogen atoms were included at calculated positions with isotropic U_factors_ = 1.2×U_eq_ or U_factors_ = 1.5×U_eq_ for hydroxyl groups (U_eq_ being the equivalent isotropic thermal factor of the bonded non hydrogen atom). Hydrogen atoms have been places on water molecules when supported by electron density Fourier difference maps while electron density contribution that couldn't be modelled (i.e. in [Gd(*ent-*L_2_)] cavities) have been removed with Platon SQUEEZE^[61]^ routine (3234 electrons in 8496 Å^3^). Estimated contribution of these regions have been included in the reported crystal properties (**Tables S4** and **S9**). Stereocenters have been experimentally assigned using Flack parameter^[62]^ for chiral space groups. Figures were prepared using Ortep-3,^[63]^ CCDC Mercury^[64]^ and PyMOL^[65]^ software.

## **3.3 X-ray structure of [Cu(H_4_*rac*-L_1_)], [Gd(PCTA)(CO_3_)]^2-^, [Gd(H_3_*ent*-L_1_)(C_2_O_4_)]^2-^ and [Gd(*ent*-L_2_)(CO_3_)]^2-^**

All crystals analysed in these studies show one crystallographically independent molecule in the asymmetric unit (**Figure S16**).

**A
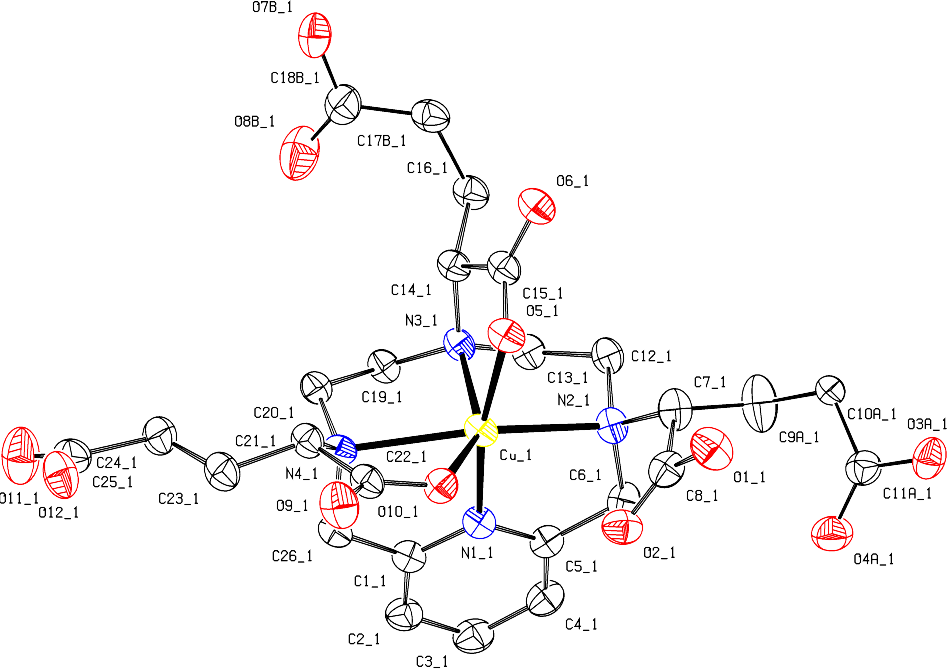
 B
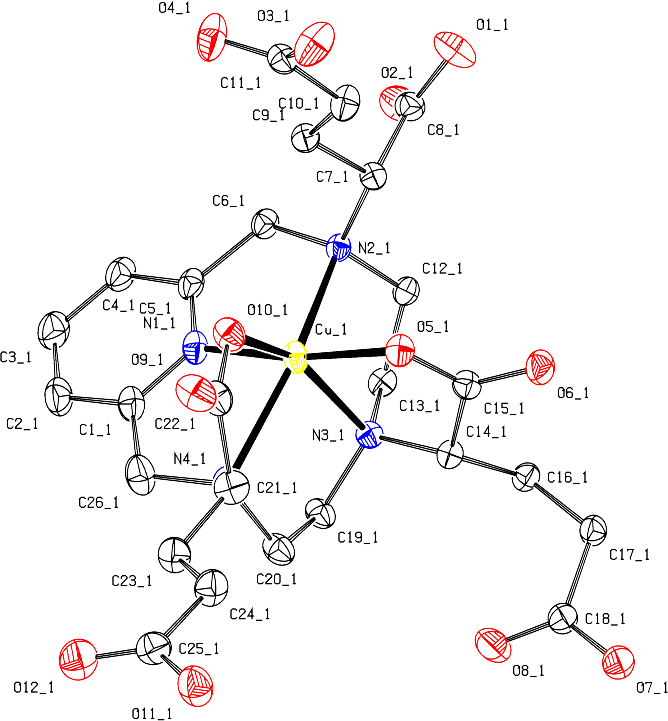
**

**C**
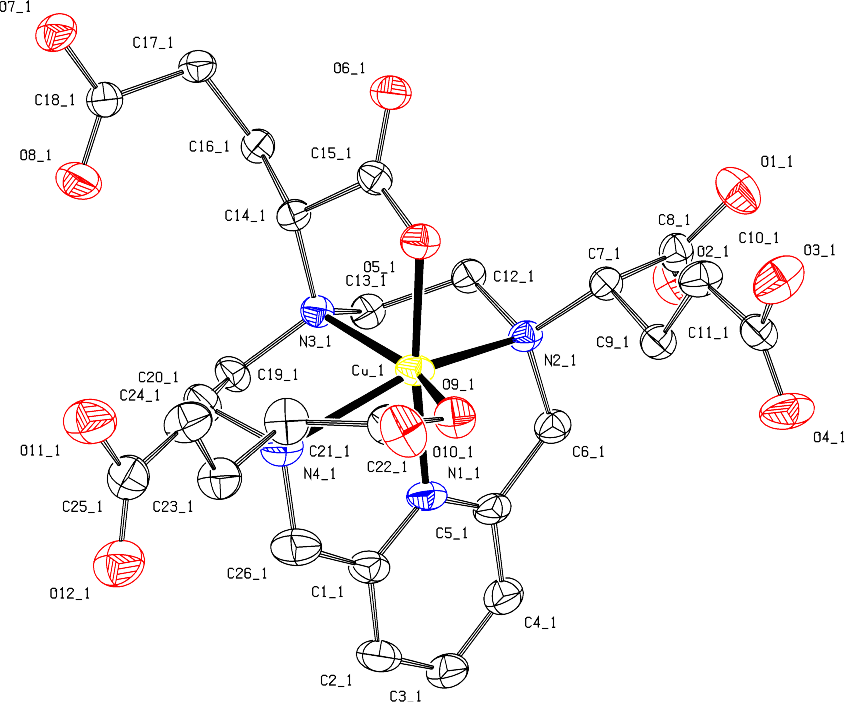
**D**
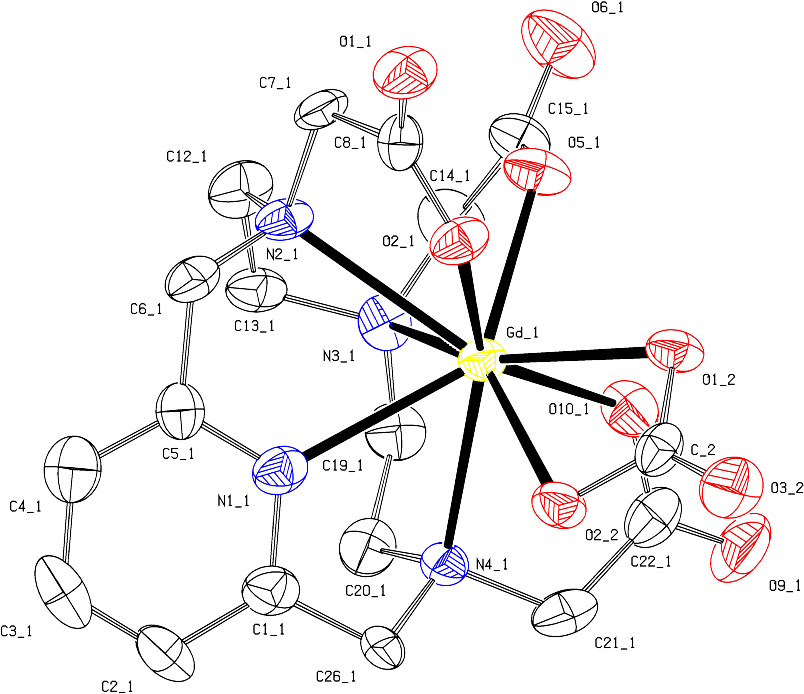


**E**
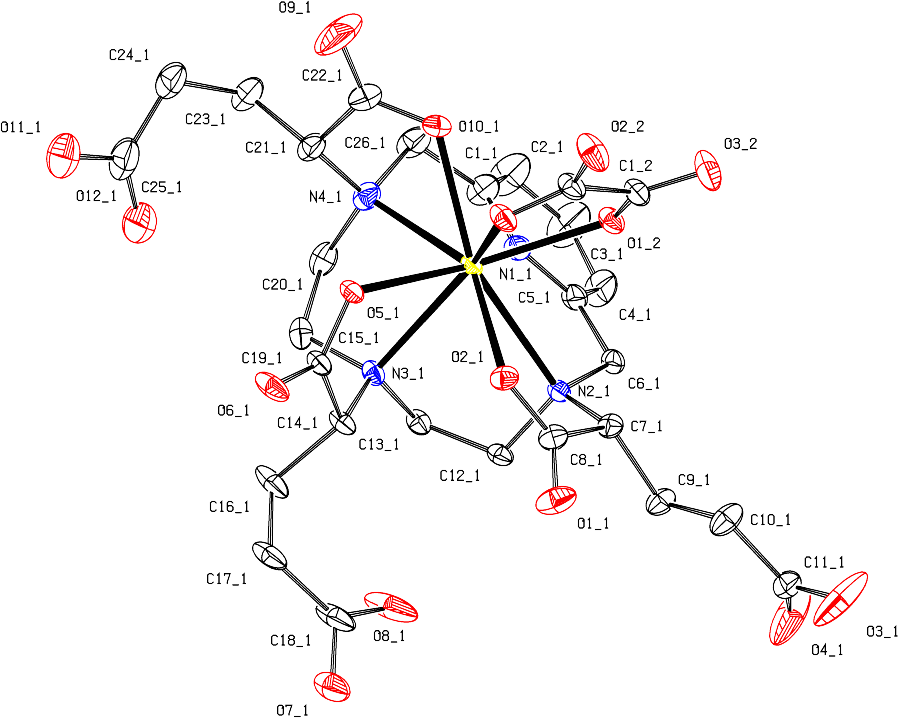
 **F**
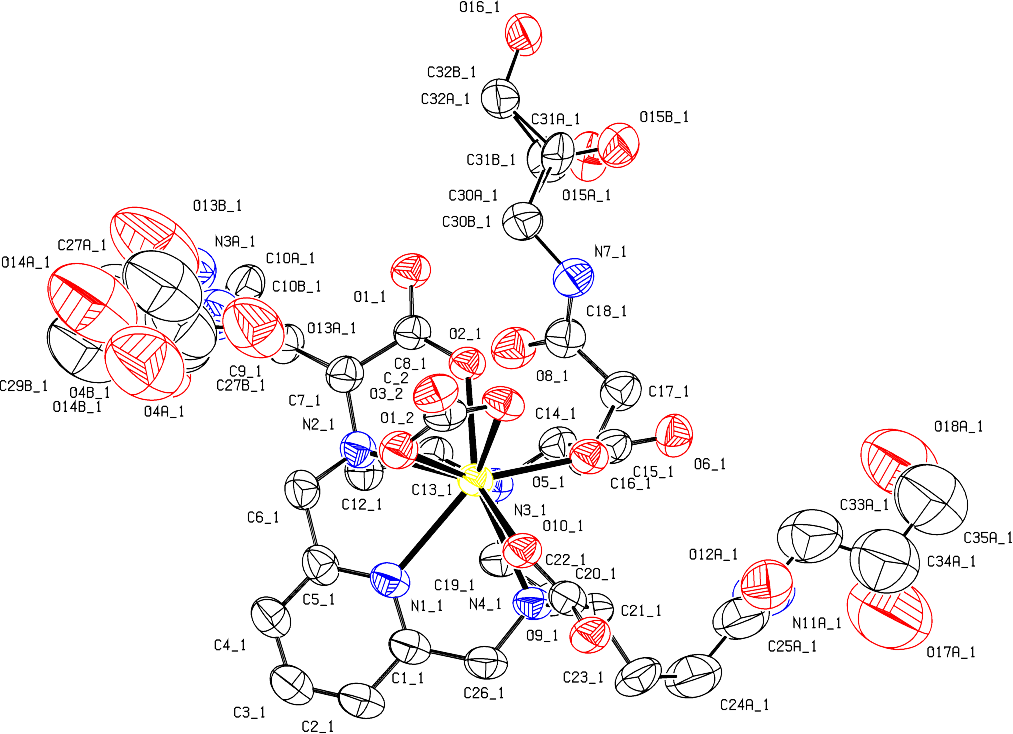


**Figure S16.** Ellipsoids representation of molecular conformations found in crystallographic asymmetric unit (ASU) of *RRS*-*SSR* racemate (**A**), *RSR* (**B**) and *SRS* (**C**) enantiomers of [Cu(H_4_*rac*-L_1_)], [Gd(PCTA)(CO_3_)]^2-^ (**D**), [Gd(H_3_*ent*-L_1_)(C_2_O_4_)]^2-^ (**E**) and [Gd(*ent*-L_2_)(CO_3_)]^2-^ (**F**) (50% probability – hydrogens, disorder and solvent omitted for clarity).

The “fiber” and “cubic” crystals of the tetraprotonated [Cu(H_4_*rac*-L_1_)] complex belong to the chiral orthorhombic (*P* 2_1_2_1_2_1_) and tricilinic (*P* -1) space groups (**Figures S17** and **S18**). Experimental details of the X-ray structure determination for “fiber” and “cubic” tetraprotonated [Cu(H_4_*rac*-L_1_)] complex are reported in Table S4.  Geometrical parameters including the hydrogen bonds found in the crystal packing of [Cu(C_26_H_34_N_4_O_12_)] ⋅ 4H_2_O (“fiber”) and [Cu(C_26_H_34_N_4_O_12_)] ⋅ H_2_O (“cubic”) single crystals are summarized in **Tables S5 – S8**.

The X-ray structure of “fiber” and “cubic” [Cu(H_4_*rac*-L_1_)] crystals reveals that the coordination around Cu(II) is given by four nitrogen and two carboxylate oxygen donor atoms in a distorted octahedral geometry. The coordination sites of Cu(II) are occupied by two of the ring N-atoms (N1, N3) and two carboxylate O-atoms (O5, O10) in a square planar fashion in equatorial position. The other two N-atoms (N2, N4) of the ring in axial position complete the coordination sphere. Solid state structure also indicates that all remote carboxylate and one of the closest carboxylate oxygen atoms are protonated and non-coordinated. The “fiber” crystal of [Cu(H_4_*rac*-L_1_)] in Figure S17 reveals the presence of conglomerate mixture containing equal amounts of *SRS* and *RSR* crystals as a configuration of α-glutarate (C7, C14, C21) groups. However, the “cubic” crystals of [Cu(H_4_*rac*-L_1_)] in Figure S18 contain both *RRS* and *SSR* isomers as a configuration of α-glutarate (C7, C14, C21) groups. The solid-state structure of the [Cu(H_4_*rac*-L_1_)] is very similar to that of the analogues [Cu(H_2_DOTA)] complex (two opposite carboxylate oxygen atoms are protonated in [Cu(H_2_DOTA)]), the coordination of Cu(II) is completed by two carboxylate oxygen and the two ring nitrogen atoms in the equatorial plane and by two ring nitrogen atoms in the axial positions.^[66]^

**A**  **B**

**Figure S17.** View of the [Cu(H_4_*rac*-L_1_)] with the configuration of the stereocenters C7: R, C14: S and C21: R (**A**) and C7: S, C14: R and C21: S (**B**) found in the asymmetric unit of the “fiber” single crystals (hydrogen atoms and disorder omitted for clarity).

**A** **B**

**Figure S18.** View of the [Cu(H_4_*rac*-L_1_)] with the configuration of the stereocenters C7: R, C14: R and C21: S (**A**) and their enantiomeric form C7: S, C14: S and C21: R (**B**) found in the asymmetric unit of the “cubic” single crystals. (hydrogen atoms and disorder omitted for clarity).

**Table S4.** Experimental details of X-ray structure determination of the tetraprotonated [Cu(H_4_*rac*-L_1_)] complexes. Reported stereocenter configurations refer to chiral atoms closer to metal centre (i.e. C7, C14 and C21)

|  |  | **[Cu(H_4_*rac*-L_1_)]** |  |
| --- | --- | --- | --- |
| **Chemical formula** | [Cu(C_26_H_34_N_4_O_12_)] ⋅ H_2_O (“cubic”; *RRS-SSR* racemate) | [Cu(C_26_H_34_N_4_O_12_)] ⋅ 4H_2_O  (“fiber”; *RSR* enantiomer) | [Cu(C_26_H_34_N_4_O_12_)] ⋅ 4H_2_O  (“fiber”; *SRS* enantiomer) |
| **CCDC Number** | 2368968 | 2368969 | 2368970 |
| ***M*_r_** | 676.13 | 730.17 | 730.17 |
| **Crystal system, space group** | Triclinic, *P* -1 | Orthorhombic, *P* 2_1_2_1_2_1_ | Orthorhombic, *P* 2_1_2_1_2_1_ |
| **Temperature [K]** | 100(2) | 100(2) | 100(2) |
| ***a*, *b*, *c* [Å]** | 9.926(2),  10.757(2),  13.459(3) | 13.705(3),  14.741(3),  15.221(3) | 13.687(3),  14.663(3),  15.293(3) |
| **α, β, γ [°]** | 102.27(3),  98.88(3),  100.03(3) | 90,  90,  90 | 90,  90,  90 |
| ***V* [Å^3^]** | 1355.2(5) | 3075.0(11) | 3069.2(11) |
| ***Z*** | 2 | 4 | 4 |
| **ρ [g cm^-3^]** | 1.657 | 1.577 | 1.580 |
| **μ [mm^-1^]** | 0.849 | 0.761 | 0.763 |
| **Crystal size [mm]** | 0.10🞨0.02🞨0.02 | 0.09🞨0.05🞨0.02 | 0.09🞨0.05🞨0.02 |
| **F(000)** | 706 | 1532 | 1532 |
| **Data collection** | | | |
| **θ_min_, θ_max_ [°]** | 1.556, 33.219 | 1.894, 33.039 | 1.895, 33.212 |
| **Resolution [Å]** | 0.64 | 0.64 | 0.64 |
| **Total refl. collctd.** | 46874 | 35644 | 35654 |
| **Independent refl.** | 9198 | 10118 | 10830 |
| **Obs. Refl. Fo>4σFo** | 6057 | 9751 | 8309 |
| **I/σ (all data)** | 10.08 | 31.53 | 15.20 |
| **I/σ (max resltn)** | 2.64 | 17.89 | 4.09 |
| **Rmerge (all data)** | 9.98% | 5.67% | 7.56% |
| **Rmerge (max resltn)** | 37.31% | 10.17% | 28.27% |
| **Completeness (all data)** | 84.6% | 84.4% | 89.8% |
| **Multiplicity (all data)** | 4.31 | 5.33 | 5.27 |
| **Multiplicity (max resltn)** | 2.42 | 3.15 | 3.09 |
| **Refinement** | | | |
| **Data/restraint/parameters** | 22655 / 788 / 837 | 12347 / 23 / 600 | 4717 / 13 / 421 |
| **RI>2σI,wR2,I>2σI** | 5.80%, 16.25% | 2.81%, 7.01% | 9.03%, 18.63% |
| **R (all data), wR2 (all data)** | 9.46%, 18.60% | 2.85%, 7.03% | 11.48%, 19.57% |
| **GooF** | 1.068 | 1.044 | 1.189 |

**Table S5.** Geometric parameters for metal coordination sphere in [Cu(C_26_H_34_N_4_O_12_)] ⋅ H_2_O (“cubic”). Reported stereocenter configurations refer to chiral atoms closer to metal centre (i.e. C7, C14 and C21). Atom naming is shown in Figure S16.

| **[Cu(H_4_*rac*-L_1_)] (“cubic” *RRS-SSR* – racemate)** | | | | |
| --- | --- | --- | --- | --- |
| **Distances** | **[Å]** | **Angles** | **[°]** |
| Cu_1-O5_1 | 1.932(2) | O10_1-Cu_1-O5_1 | 89.83(8) |
| Cu_1-O10_1 | 2.048(2) | N2_1-Cu_1-O5_1 | 87.38(9) |
| Cu_1-N1_1 | 1.992(2) | N3_1-Cu_1-O5_1 | 83.31(8) |
| Cu_1-N2_1 | 2.330(2) | N4_1-Cu_1-O5_1 | 113.40(8) |
| Cu_1-N3_1 | 2.153(2) | N2_1-Cu_1-O10_1 | 127.70(8) |
| Cu_1-N4_1 | 2.388(2) | N4_1-Cu_1-O10_1 | 74.45(8) |
|  |  | O10_1-Cu_1-N1_1 | 100.33(9) |
|  |  | N2_1-Cu_1-N1_1 | 79.44(9) |
|  |  | N3_1-Cu_1-N1_1 | 92.34(8) |
|  |  | N4_1-Cu_1-N1_1 | 78.14(9) |
|  |  | N2_1-Cu_1-N3_1 | 82.99(8) |
|  |  | N4_1-Cu_1-N3_1 | 80.02(8) |

**Table S6.** Geometric parameters for metal coordination sphere in [Cu(C_26_H_34_N_4_O_12_)] ⋅ 4H_2_O (“fiber”). Reported stereocenter configurations refer to chiral atoms closer to metal centre (i.e. C7, C14 and C21). Atom naming is shown in Figure S16.

| **[Cu(H_4_*rac*-L_1_)] (“fiber” *RSR-SRS* enantiomers)** | | | | | | | | |
| --- | --- | --- | --- | --- | --- | --- | --- | --- |
| **RSR** | | | |  | **SRS** | | | | |
| **Distances** | **[Å]** | **Angles** | **[°]** |  | **Distances** | **[Å]** | **Angles** | **[°]** |
| Cu_1-O5_1 | 1.942(2) | O10_1-Cu_1-O5_1 | 88.63(8) |  | Cu_1-O5_1 | 1.946(3) | O10_1-Cu_1-O5_1 | 88.93(13) |
| Cu_1-O10_1 | 2.032(2) | N2_1-Cu_1-O5_1 | 91.62(7) |  | Cu_1-O10_1 | 2.023(3) | N2_1-Cu_1-O5_1 | 91.56(13) |
| Cu_1-N1_1 | 2.002(2) | N3_1-Cu_1-O5_1 | 83.61(8) |  | Cu_1-N1_1 | 2.010(3) | N3_1-Cu_1-O5_1 | 83.44(13) |
| Cu_1-N2_1 | 2.306(2) | N4_1-Cu_1-O5_1 | 110.58(8) |  | Cu_1-N2_1 | 2.304(4) | N4_1-Cu_1-O5_1 | 110.98(14) |
| Cu_1-N3_1 | 2.111(2) | N2_1-Cu_1-O10_1 | 121.08(8) |  | Cu_1-N3_1 | 2.112(3) | N2_1-Cu_1-O10_1 | 121.00(14) |
| Cu_1-N4_1 | 2.302(2) | N4_1-Cu_1-O10_1 | 76.80(8) |  | Cu_1-N4_1 | 2.303(4) | N4_1-Cu_1-O10_1 | 77.09(14) |
|  |  | O10_1-Cu_1-N1_1 | 95.38(9) |  |  |  | O10_1-Cu_1-N1_1 | 95.38(14) |
|  |  | N2_1-Cu_1-N1_1 | 79.06(8) |  |  |  | N2_1-Cu_1-N1_1 | 78.87(14) |
|  |  | N3_1-Cu_1-N1_1 | 96.33(8) |  |  |  | N3_1-Cu_1-N1_1 | 96.22(14) |
|  |  | N4_1-Cu_1-N1_1 | 78.63(9) |  |  |  | N4_1-Cu_1-N1_1 | 78.40(15) |
|  |  | N2_1-Cu_1-N3_1 | 84.80(7) |  |  |  | N2_1-Cu_1-N3_1 | 84.58(12) |
|  |  | N4_1-Cu_1-N3_1 | 82.06(8) |  |  |  | N4_1-Cu_1-N3_1 | 82.11(13) |

**Table S7.** Hydrogen-bond geometry for [Cu(C_26_H_34_N_4_O_12_)] ⋅ H_2_O (“cubic” *RRS-SSR* – racemate). Reported stereocenter refer to chiral atoms closer to metal center (i.e. C7, C14 and C21).

| **D-H···A d(D-H) [Å] d(H···A) [Å] d(D···A) [Å] <(DHA)[°]** |
| --- |
| O3A_1^a-H3A_1^a...O9_1#1 0.84 1.60 2.426(4) 168.3  O3B_1^b-H3B_1^b...O9_1#1 0.84 1.89 2.713(11) 168.1  O7B_1^b-H7B_1^b...O10_1#2 0.84 2.10 2.879(7) 153.1  O7A_1^a-H7A_1^a...O4A_1^a#3 0.84 1.97 2.670(6) 139.6  O1_1-H1_1...O2_1#1 0.84 1.84 2.680(3) 173.7  O12_1-H12_1...O_2#4 0.84 1.77 2.610(3) 177.5  O_2-H1_2...O8B_1^b 0.873(10) 1.97(2) 2.823(16) 164(4)  O_2-H1_2...O8A_1^a 0.873(10) 1.96(2) 2.786(12) 157(4)  O_2-H2_2...O6_1#2 0.867(10) 1.904(12) 2.767(3) 173(4) |
| Symmetry transformations used to generate equivalent atoms:  #1: -x+1,-y+1,-z+2; #2: -x+1,-y+1,-z+1; #3: x,y,z-1; #4: -x+2,-y+2,-z+1 |

**Table S8.** Hydrogen-bond geometry for [Cu(C_26_H_34_N_4_O_12_)] ⋅ 4H_2_O (“fiber” *RSR* and *SRS* enantiomers). Reported stereocenter refer to chiral atoms closer to metal center (i.e. C7, C14 and C21).

| ***RSR*** |
| --- |
| **D-H···A d(D-H) [Å] d(H···A) [Å] d(D···A)[Å] <(DHA)[°]** |
| O7_1-H7_1...O6_1#1 0.84 1.90 2.653(3) 149.0  O11_1-H11_1...O9_1#2 0.84 1.70 2.511(3) 161.0  O1_1-H1_1...O_2 0.84 1.73 2.570(4) 172.9  O3_1-H3_1...O9_1#3 0.84 1.87 2.710(3) 176.6  O_2-H1_2...O8_1#4 0.870(14) 1.82(4) 2.656(4) 160(9)  O_2-H2_2...O12_1#5 0.873(14) 2.12(3) 2.956(4) 161(9)  O_3-H1_3...O5_1 0.870(13) 2.034(14) 2.899(3) 173(4)  O_3-H2_3...O12_1#3 0.867(13) 2.21(3) 2.956(4) 145(4)  O_4-H1_4...O2_1#6 0.877(14) 2.56(5) 3.059(4) 117(4)  O_4-H1_4...O3_1 0.877(14) 2.32(3) 3.152(4) 159(6)  O_4-H2_4...O_3 0.879(13) 2.06(3) 2.849(4) 150(5)  O_5-H1_5...O4_#7 0.889(14) 2.09(3) 2.893(4) 149(5) |
| Symmetry transformations used to generate equivalent atoms:  #1: x-1/2,-y+1/2,-z+1; #2: x-1/2,-y+3/2,-z+1; #3: x+1/2,-y+3/2,-z+1; #4: x+1,y,z;  #5: -x+1,y-1/2,-z+1/2; #6: -x+3/2,-y+1,z+1/2; #7: -x+3/2,-y+1,z-1/2 |
| ***SRS*** |
| **D-H···A d(D-H) [Å] d(H···A) [Å] d(D···A)[Å] <(DHA)[°]** |
| O7_1-H7_1...O6_1#1 0.84 1.89 2.651(5) 150.3  O11_1-H11_1...O10_1#2 0.84 2.61 3.320(6) 142.5  O11_1-H11_1...O9_1#2 0.84 1.75 2.505(6) 147.7  O1_1-H1_1...O_2 0.84 1.73 2.564(6) 174.7  O3_1-H3_1...O9_1#3 0.84 1.88 2.702(5) 167.2  O_2-H1_2...O8_1#4 0.871(14) 1.80(5) 2.631(6) 159(13)  O_2-H2_2...O12_1#5 0.872(14) 2.125(17) 2.996(8) 178(12)  O_3-H1_3...O5_1 0.875(14) 2.024(16) 2.891(5) 171(6)  O_3-H2_3...O12_1#3 0.868(14) 2.23(5) 2.962(6) 142(7)  O_4-H1_4...O3_1 0.875(14) 2.30(3) 3.150(7) 164(8)  O_4-H2_4...O_3 0.878(14) 2.12(6) 2.843(7) 139(7)  O_5-H1_5...O_4#6 0.881(14) 2.30(8) 2.880(8) 123(8) |
| Symmetry transformations used to generate equivalent atoms:  #1: x+1/2,-y+3/2,-z+1; #2: x+1/2,-y+1/2,-z+1; #3: x-1/2,-y+1/2,-z+1; #8: x-1,y,z;  #5: -x+1,y+1/2,-z+3/2; #6: -x+1/2,-y+1,z+1/2; #7: -x+1,y-1/2,-z+3/2 |

The [Gd(PCTA)CO_3_]^2-^, [Gd(H_3_*ent*-L_1_)C_2_O_4_]^2-^ and [Gd(*ent*-L_2_)CO_3_]^2-^ ternary complexes are crystallized in a centrosymmetric monoclinic (*P* 2_1_*/n*), monoclinic (*P* 2_1_*/c*) and trigonal (*R*-3) space groups. The coordination environment of Gd(III) ion in [Gd(PCTA)CO_3_]^2-^, [Gd(H_3_*ent*-L_1_)C_2_O_4_]^2-^ and [Gd(*ent*-L_2_)CO_3_]^2-^ ternary complexes are shown in **Figures S19, S21 and S23**. In all three complexes the Gd(III) ion is coordinated by seven donor atoms of the PCTA, *ent*-L_1_ and *ent*-L_2_ ligands, whereas the eighth and ninth coordination sites are occupied by an η^2^-carbonate or η^2^-oxalate ligands, coordinating in a bidentate fashion (Figures S19, S21 and S23). The coordination polyhedron around the Gd(III) ion in [Gd(PCTA)CO_3_]^2-^, [Gd(H_3_*ent* -L_1_)C_2_O_4_]^2-^ and [Gd(*ent*-L_2_)CO_3_]^2-^ can be best described as a distorted monocapped square antiprism (SAP: [Gd(PCTA)CO_3_]^2-^ and [Gd(*ent*-L_2_)CO_3_]^2-^) and monocapped twisted square antiprism (TSAP: [Gd(H_3_*ent*-L_1_)C_2_O_4_]^2-^) defined by two nearly parallel pseudo planes with the upper one formed by O1, O2, O5, O10 (mean atomic distances: 0.014, 0.061 and 0.059 Å for [Gd(PCTA)CO_3_]^2-^, [Gd(H_3_*ent*-L_1_)C_2_O_4_]^2-^ and [Gd(*ent*-L_2_)CO_3_]^2-^) as well as the lower one with N1, N2, N3, N4 (mean atomic distances: 0.271, 0.245 and 0.248 Å for [Gd(PCTA)CO_3_]^2-^, [Gd(H_3_*ent*-L_1_)C_2_O_4_]^2-^ and [Gd(*ent*-L_2_)CO_3_]^2-^). The angle of the square planes is 9.2, 6.5 and 9.1° in [Gd(PCTA)CO_3_]^2-^, [Gd(H_3_*ent*-L_1_)C_2_O_4_]^2-^ and [Gd(*ent*-L_2_)CO_3_]^2-^, respectively. Somewhat smaller angle of the square planes in [Gd(H_3_*ent*-L_1_)C_2_O_4_]^2-^ can be explained with the larger chelate ring formed by the coordination of the oxalate ion in a bidentate fashion. The other oxygen of the carbonate or oxalate ion occupies the capping position above the upper plane. Average torsion angles between the two square planes defined by the oxygen and nitrogen atoms are 35°, 27° and 37° for [Gd(PCTA)CO_3_]^2-^, [Gd(H_3_*ent*-L_1_)C_2_O_4_]^2-^ and [Gd(*ent*-L_2_)CO_3_]^2-^, respectively. Average bond distances in the ternary [Gd(PCTA)CO_3_]^2-^, [Gd(H_3_*ent*-L_1_)C_2_O_4_]^2-^ and [Gd(*ent*-L_2_)CO_3_]^2-^ complexes were found to be 2.391, 2.378 and 2.377 Å (Gd–O) and 2.629, 2.655 and 2.628 Å (Gd–N), respectively. Solid state structure of the ternary [Gd(H_3_*ent*-L_1_)C_2_O_4_]^2-^ complex also indicates that all remote carboxylate groups are protonated and non-coordinated. Experimental details of the X-ray structure determination for [Gd(PCTA)CO_3_]^2-^, [Gd(*ent*-L_1_)C_2_O_4_]^2-^ and [Gd(*ent*-L_2_)CO_3_]^2-^ complexes are reported in Table S9. The crystal packing of {(C(NH_2_)_3_)_2_[Gd(PCTA)(CO_3_)]}·4H_2_O, {(C(NH_2_)_3_)_2_[Gd(C_26_H_34_N_4_O_12_)(C_2_O_4_)]}·1H_2_O and {(C(NH_2_)_3_)_2_[Gd(C_35_H_54_N_7_O_15_)(CO_3_)]}·18H_2_O single crystal are shown in (**Figures S20, S22 and S24**). Geometrical parameters including the hydrogen bonds found in the crystal packing of {(C(NH_2_)_3_)_2_[Gd(PCTA)(CO_3_)]}·4H_2_O, {(C(NH_2_)_3_)_2_[Gd(C_26_H_34_N_4_O_12_)(C_2_O_4_)]}·1H_2_O and {(C(NH_2_)_3_)_2_[Gd(C_35_H_54_N_7_O_15_)(CO_3_)]}·18H_2_O single crystals are summarized in **Tables S10 – S15**.

**Table S9.** Experimental details of X-ray structure determination of the Gd(III)-complexes with PCTA, *ent*-L_1_ and *ent*-L_2_ ligands. Reported stereocenter refer to chiral atoms closer to metal center (i.e. C7, C14 and C21).

|  | **[Gd(*ent*-L_2_)(CO_3_)]^2-^**  **(*RRR-SSS* racemate)** | **[Gd(H_3_*ent*-L_1_)(C_2_O_4_)]^2-^**  **(*RRR-SSS* racemate)** | **[Gd(PCTA)(CO_3_)]^2-^** |
| --- | --- | --- | --- |
| **Chemical formula** | [Cu(C_35_H_54_N_7_O_15_)](CO_3_)  (CH_6_N_3_)_2_ ⋅ 22H_2_O | [Cu(C_26_H_33_N_4_O_12_)](C_2_O_4_)  (CH_6_N_3_)_2_ ⋅ 5H_2_O | [Cu(C_17_H_21_N_4_O_6_)] (CO_3_)  (CH_6_N_3_)_2_ ⋅ 4H_2_O |
| **CCDC Number** | 2368971 | 2368972 | 2368973 |
| ***M*_r_** | 1546.63 | 1049.09 | 786.88 |
| **Crystal system, space group** | Trigonal, *R* -3 | Monoclinic, *P* 2_1_*/c* | Monoclinic, *P* 2_1_*/n* |
| **Temperature [K]** | 100(2) | 100(2) | 296(2) |
| ***a*, *b*, *c* [Å]** | 53.395(8),  53.395(8),  12.959(3) | 10.682(2),  36.733(7),  10.521(2) | 13.002(2),  14.362(2),  16.135(2) |
| **α, β, γ [°]** | 90,  90,  120 | 90,  90.80(3),  90 | 90,  100.705(4),  90 |
| ***V* [Å^3^]** | 31997(11) | 4127.9(14) | 3069.2(11) |
| ***Z*** | 18 | 4 | 4 |
| **ρ [g cm^-3^]** | 1.445 | 1.688 | 1.765 |
| **μ [mm^-1^]** | 0.988 | 1.628 | 2.320 |
| **Crystal size [mm]** | 0.08🞨0.01🞨0.01 | 0.10🞨0.05🞨0.01 | 0.22🞨0.20🞨0.12 |
| **F(000)** | 14634 | 2148 | 1596 |
| **Data collection** | | | |
| **θ_min_, θ_max_ [°]** | 0.751, 33.219 | 1.983, 29.999 | 1.852, 24.257 |
| **Resolution [Å]** | 0.64 | 0.70 | 0.86 |
| **Total refl. collctd.** | 151263 | 103211 | 54020 |
| **Independent refl.** | 22655 | 12347 | 4717 |
| **Obs. Refl. Fo>4σFo** | 14424 | 12113 | 3752 |
| **I/σ (all data)** | 13.28 | 70.26 | 16.86 |
| **I/σ (max resltn)** | 2.50 | 51.36 | 9.29 |
| **Rmerge (all data)** | 9.16% | 2.22% | 15.32% |
| **Rmerge (max resltn)** | 49.36% | 2.99% | 38.90% |
| **Completeness (all data)** | 95.1% | 98.1% | 99.3% |
| **Multiplicity (all data)** | 6.35 | 8.13 | 11.03 |
| **Multiplicity (max resltn)** | 5.37 | 7.27 | 11.17 |
| **Refinement** | | | |
| **Data/restraint/parameters** | 22655 / 788 / 837 | 12347 / 23 / 600 | 4717 / 13 / 421 |
| **RI>2σI,wR2,I>2σI** | 5.80%, 16.25% | 2.81%, 7.01% | 9.03%, 18.63% |
| **R (all data), wR2 (all data)** | 9.46%, 18.60% | 2.85%, 7.03% | 11.48%, 19.57% |
| **GooF** | 1.068 | 1.044 | 1.189 |

**Figure S19.** View of the Gd(III)-coordination environment in [Gd(PCTA)(CO_3_)]^2-^ of the single crystal with the formula {(C(NH_2_)_3_)_2_[Gd(PCTA)(CO_3_)]}·4H_2_O.


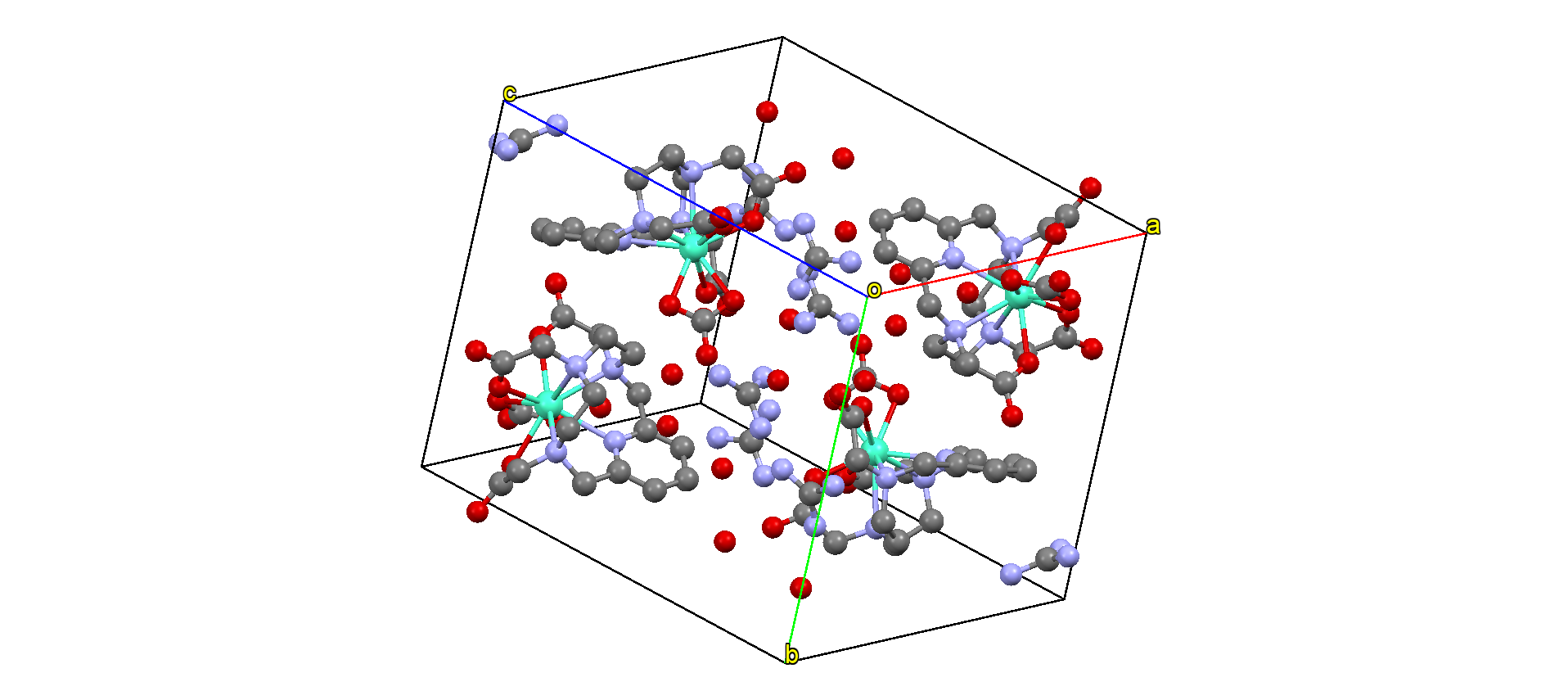


**Figure S20.** Packing diagram of {(C(NH_2_)_3_)_2_[Gd(PCTA)(CO_3_)]}·4H_2_O. Hydrogen atoms are omitted for simplicity.

**Table S10.** Geometric parameters for metal coordination sphere in {(C(NH_2_)_3_)_2_[Gd(PCTA)(CO_3_)]}·4H_2_O. Atom naming is shown in Figure S16.

| **Distances** | **[Å]** | **Angles** | **[°]** | **Angles** | **[°]** | **Angles** | **[°]** |
| --- | --- | --- | --- | --- | --- | --- | --- |
| Gd_1-O5_1 | 2.355(9) | N1_1-Gd_1-O2_1 | 91.63(36) | O1_2-Gd_1-O10_1 | 74.04(34) | O2_1-Gd_1-O1_2 | 71.64(33) |
| Gd _1-O2_2 | 2.386(9) | N2_1-Gd_1-O2_1 | 62.68(32) | O2_1-Gd_1-O10_1 | 140.66(34) | N1_1-Gd_1-O1_2 | 135.27(34) |
| Gd _1-O10_1 | 2.386(10) | N3_1-Gd_1-O2_1 | 128.14(36) | N1_2-Gd_1-O10_1 | 126.74(36) | N2_1-Gd_1-O1_2 | 131.54(33) |
| Gd _1-O1_2 | 2.410(10) | N4_1-Gd_1-O2_1 | 147.31(35) | N4_2-Gd_1-O10_1 | 65.89(35) | N3_1-Gd_1-O1_2 | 140.95(34) |
| Gd _1-O2_1 | 2.438(9) | O2_2-Gd_1-O5_1 | 139.08(33) | N3_2-Gd_1-O10_1 | 73.48(37) | N4_1-Gd_1-O1_2 | 113.23(34) |
| Gd _1-N1_1 | 2.594(12) | O10_1-Gd_1-O5_1 | 76.04(37) | N2_2-Gd_1-O10_1 | 137.54(36) | O1_2-Gd_1-O2_2 | 54.12(31) |
| Gd _1-N4_1 | 2.600(11) | O1_2-Gd_1-O5_1 | 85.03(33) | N2_1-Gd_1-N1_1 | 63.64(37) | O2_1-Gd_1-O2_2 | 82.29(34) |
| Gd _1-N3_1 | 2.617(11) | O2_1-Gd_1-O5_1 | 82.36(34) | N3_1-Gd_1-N1_1 | 81.98(36) | O10_1-Gd_1-O2_2 | 92.92(37) |
| Gd _1-N2_1 | 2.703(12) | N1_1-Gd_1-O5_1 | 134.82(38) | N4_1-Gd_1-N1_1 | 61.55(37) | N1_1-Gd_1-O2_2 | 83.29(35) |
|  |  | N4_1-Gd_1-O5_1 | 129.47(34) | N2_1-Gd_1-N4_1 | 113l81(34) | N4_1-Gd_1-O2_2 | 76.62(34) |
|  |  | N3_1-Gd_1-O5_1 | 67.89(35) | N3_1-Gd_1-N4_1 | 69.75(37) | N3_1-Gd_1-O2_2 | 146.34(36) |
|  |  | N2_1-Gd_1-O5_1 | 74.11(37) |  |  | N2_1-Gd_1-O2_2 | 129.23(35) |

**Table S11.** Hydrogen-bond geometry for {(C(NH_2_)_3_)_2_[Gd(PCTA)(CO_3_)]}·4H_2_O. Atom naming is shown in Figure S16.

| **D-H···A d(D-H) [Å] d(H···A) [Å] d(D···A) [Å] <(DHA) [°]** |
| --- |
| N3_3-H3A_3...O2_1#1 0.86 2.09 2.929(16) 163.7  N3_3-H3B_3...O3_2#1 0.86 2.48 3.048(18) 124.1  N2_3-H2A_3...O1_2 0.86 2.06 2.898(15) 163.0  N2_3-H2B_3...O1_1#1 0.86 2.04 2.902(17) 174.6  N1_3-H1A_3...O5_1 0.86 2.01 2.855(16) 165.4  N1_3-H1B_3...O_6#2 0.86 2.01 2.86(2) 170.4  N3_4-H3A4...O_8#3 0.86 2.43 3.04(3) 128.3  N3_4-H3B_4...O_6 0.86 2.05 2.87(3) 158.3  N2_4-H2A_4...O1_2 0.86 2.12 2.938(17) 159.4  N2_4-H2B_4...O_8 0.86 2.62 3.25(3) 130.6  N1_4-H1A_4...O10_1 0.86 2.11 2.91(2) 154.4  N1_4-H1B_4...O6_1#4 0.86 2.12 2.97(2) 169.8  O_5-H15...O3_2#3 0.870(11) 1.94(10) 2.769(18) 159(25)  O_5-H2_5...O2_2#5 0.870(11) 2.05(7) 2.855(18) 153(15)  O_5-H2_5...O3_2#5 0.870(11) 2.59(17) 3.325(19) 143(24)  O_6-H1_6...O_7 0.870(11) 1.77(6) 2.63(3) 170(29)  O_6-H2_6...O_5 0.871(10) 2.10(19) 2.68(2) 123(18)  O_7-H2_7...O_8 0.870(11) 2.21(6) 3.07(3) 170(21)  O_8-H1_8...O3_2 0.871(11) 1.96(14) 2.678(19) 138(18)  O_8-H2_8...N3_4#6 0.871(10) 2.51(2) 3.04(3) 119(3) |
| Symmetry transformations used to generate equivalent atoms:  #1: -x+1,-y+1,-z+1; #2: x-1/2,-y+3/2,z-1/2; #3: -x+3/2,y+1/2,-z+3/2; #4 -x+1,-y+2,-z+1; #5: x-1/2,-y+3/2,z+1/2; #6: -x+3/2,y-1/2,-z+3/2 |

**Figure S21.** View of the Gd(III) coordination environment in [Gd(H_3_*ent*-L_1_)(C_2_O_4_)]^2-^ of the single crystal with the formula {(C(NH_2_)_3_)_2_[Gd(C_26_H_34_N_4_O_12_)(C_2_O_4_)]}·5H_2_O


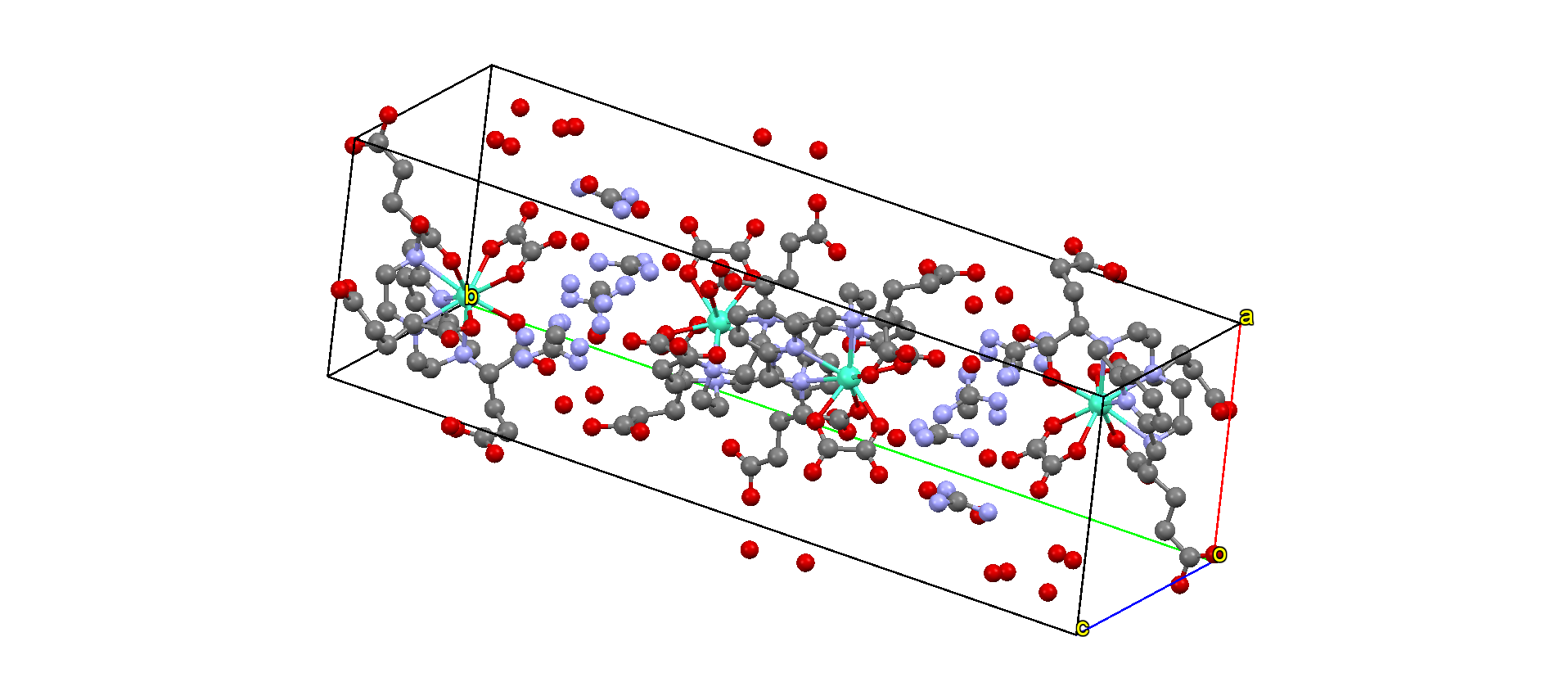


**Figure S22.** Packing diagram of {(C(NH_2_)_3_)_2_[Gd(C_26_H_34_N_4_O_12_)(C_2_O_4_)]}·5H_2_O. Hydrogen atoms are omitted for simplicity.

**Table S12.** Geometric parameters for metal coordination sphere in {(C(NH_2_)_3_)_2_[Gd(C_26_H_34_N_4_O_12_)(C_2_O_4_)]}·5H_2_O. Atom naming is shown in Figure S16.

| **Distances** | **[Å]** | **Angles** | **[°]** | **Angles** | **[°]** | **Angles** | **[°]** |
| --- | --- | --- | --- | --- | --- | --- | --- |
| Gd _1-O2_1 | 2.392(2) | O1_2-Gd_1-O2_1 | 87.49(6) | O1_2-Gd_1-O10_1 | 85.43(5) | O4_1-Gd_1-O1_2 | 66.49(5) |
| Gd_1-O5_1 | 2.339(1) | O4_2-Gd_1-O2_1 | 68.96(5) | O2_1-Gd_1-O10_1 | 135.62(6) | N1_1-Gd_1-O1_2 | 78.43(5) |
| Gd _1-O10_1 | 2.367(2) | N1_2-Gd_1-O2_1 | 126.31(5) | O4_2-Gd_1-O10_1 | 68.07(6) | N2_1-Gd_1-O1_2 | 77.05(5) |
| Gd _1-N1_1 | 2.612(2) | N2_2-Gd_1-O2_1 | 62.68(5) | N1_1-Gd_1-O10_1 | 94.91(6) | N3_1-Gd_1-O1_2 | 146.46(5) |
| Gd _1-N2_1 | 2.716(2) | N3_2-Gd_1-O2_1 | 82.19(5) | N2_1-Gd_1-O10_1 | 154.48(5) | N4_1-Gd_1-O1_2 | 123.08(6) |
| Gd _1-N3_1 | 2.604(2) | N4_2-Gd_1-O2_1 | 148.56(6) | N3_1-Gd_1-O10_1 | 123.56(6) | N1_1-Gd_1-O4_2 | 141.59(5) |
| Gd _1-N4_1 | 2.690(2) | O1_2-Gd_1-O5_1 | 142.17(5) | N4_1-Gd_1-O10_1 | 61.69(6) | N2_1-Gd_1-O4_2 | 119.51(5) |
| Gd _1-O1_2 | 2.413(2) | O4_2-Gd_1-O5_1 | 75.95(5) | N2_1-Gd_1-N1_1 | 63.69(5) | N3_1-Gd_1-O4_2 | 136.07(5) |
| Gd _1-O4_2 | 2.439(2) | O2_1-Gd_1-O5_1 | 74.63(5) | N4_1-Gd_1-N1_1 | 61.42(5) | N4_1-Gd_1-O4_2 | 126.80(5) |
|  |  | O10_1-Gd_1-O5_1 | 84.78(6) | N1_1-Gd_1-N3_1 | 82.25(5) |  |  |
|  |  | N1_1-Gd_1-O5_1 | 138.81(5) | N2_1-Gd_1-N3_1 | 69.85(5) |  |  |
|  |  | N2_1-Gd_1-O5_1 | 120.32(5) | N4_1-Gd_1-N3_1 | 68.17(6) |  |  |
|  |  | N3_1-Gd_1-O5_1 | 64.58(5) |  |  |  |  |
|  |  | N4_1-Gd_1-O5_1 | 83.06(6) |  |  |  |  |
|  |  |  |  |  |  |  |  |

**Table S13.** Hydrogen-bond geometry for {(C(NH_2_)_3_)_2_[Gd(C_26_H_34_N_4_O_12_)(C_2_O_4_)]}·5H_2_O. Many hydrogen bonds involving solvent water molecules are excluded from this table, since the corresponding hydrogens could not be located into electron density maps.

| **D-H···A d(D-H) [Å] d(H···A) [Å] d(D···A) [Å] <(DHA) [°]** |
| --- |
| N1_4-H1A_4...O3_2 0.88 2.34 3.172(3) 157.2  N1_4-H1B_4...O1_1#1 0.88 2.07 2.934(3) 166.3  N2_4-H2A_4...N2B_3^b#1 0.88 2.52 3.054(10) 119.4  N2_4-H2B_4...O4_2#1 0.88 2.57 3.107(3) 120.6  N2_4-H2B_4...O2_1#1 0.88 2.11 2.924(2) 153.4  N3_4-H3A_4...O_9 0.88 2.12 2.900(3) 146.5  N3_4-H3B_4...O2_2#2 0.88 1.99 2.861(3) 170.0  N2A_3^a-H2A_3^a...O9_1#3 0.88 1.97 2.802(4) 156.3  N2A_3^a-H2B_3^a...O51 0.88 2.05 2.922(3) 172.3  N2A_3^a-H2B_3^a...O61 0.88 2.51 3.158(3) 130.5  N3A_3^a-H3A_3^a...O10_1#3 0.88 1.99 2.820(3) 156.0  N3A_3^a-H3B_3^a...O6_1#2 0.88 2.02 2.813(3) 148.5  N1A_3^a-H1A_3^a...O4_2 0.88 2.13 2.945(4) 153.5  N1A_3^a-H1B_3^a...N3_4#3 0.88 2.61 3.160(6) 121.4  N1A_3^a-H1B_3^a...O6_1#2 0.88 2.46 3.142(5) 134.7  N2B_3^b-H2C_3^b...N2_4#4 0.88 2.63 3.054(10) 110.4  N2B_3^b-H2C_3^b...O5_1 0.88 2.10 2.949(8) 160.5  N2B_3^b-H2C_3^b...O6_1 0.88 2.52 3.092(8) 123.2  N2B_3^b-H2D_3^b...N1B_3^b#3 0.88 2.17 2.984(9) 153.2  N3B_3^b-H3C_3^b...O6_1#2 0.88 2.06 2.854(10) 149.4  N3B_3^b-H3D_3^b...O10_1#3 0.88 2.18 2.964(10) 147.5  N1B_3^b-H1C_3^b...O4_2 0.88 2.19 2.970(15) 147.8  N1B_3^b-H1C_3^b...O10_1 0.88 2.66 3.402(15) 142.9  N1B_3^b-H1D_3^b...N2B_3^b#2 0.88 2.33 2.984(9) 131.4  N1B_3^b-H1D_3^b...O6_1#2 0.88 2.24 2.988(16) 142.5  O7_1-H7_1...O1_2#4 0.84 1.81 2.641(2) 171.7  O7_1-H7_1...O3_2#4 0.84 2.57 3.110(3) 122.8  O11_1-H11_1...O_9#5 0.84 1.87 2.647(3) 152.1  C24_1-H24A_1...OB_6^b#2 0.99 2.46 3.382(13) 153.9  O3_1-H3A_1...O_8#6 0.84 1.80 2.619(3) 166.0  O_9-H1_9...O2_2 0.868(10) 1.913(15) 2.727(3) 156(3)  O_9-H1_9...O3_2 0.868(10) 2.49(3) 3.031(3) 121(3)  O_9-H2_9...OA_7^a 0.864(10) 1.988(13) 2.824(4) 163(3)  O_9-H2_9...OB_7^b 0.864(10) 1.83(3) 2.617(12) 151(4) |
| Symmetry transformations used to generate equivalent atoms:  #1: x,y,z-1; #2: x,-y+3/2,z-1/2; #3: x,-y+3/2,z+1/2; #4: x,y,z+1; #5: x-1,-y+3/2,z+1/2; #6: -x+2,-y+1,-z |

**Figure S23.** View of the Gd(III) coordination environment in [Gd(*ent*-L_2_)(CO_3_)]^2-^ of the single crystal with the formula {(C(NH_2_)_3_)_2_[Gd(C_35_H_54_N_7_O_15_)(CO_3_)]}·22H_2_O


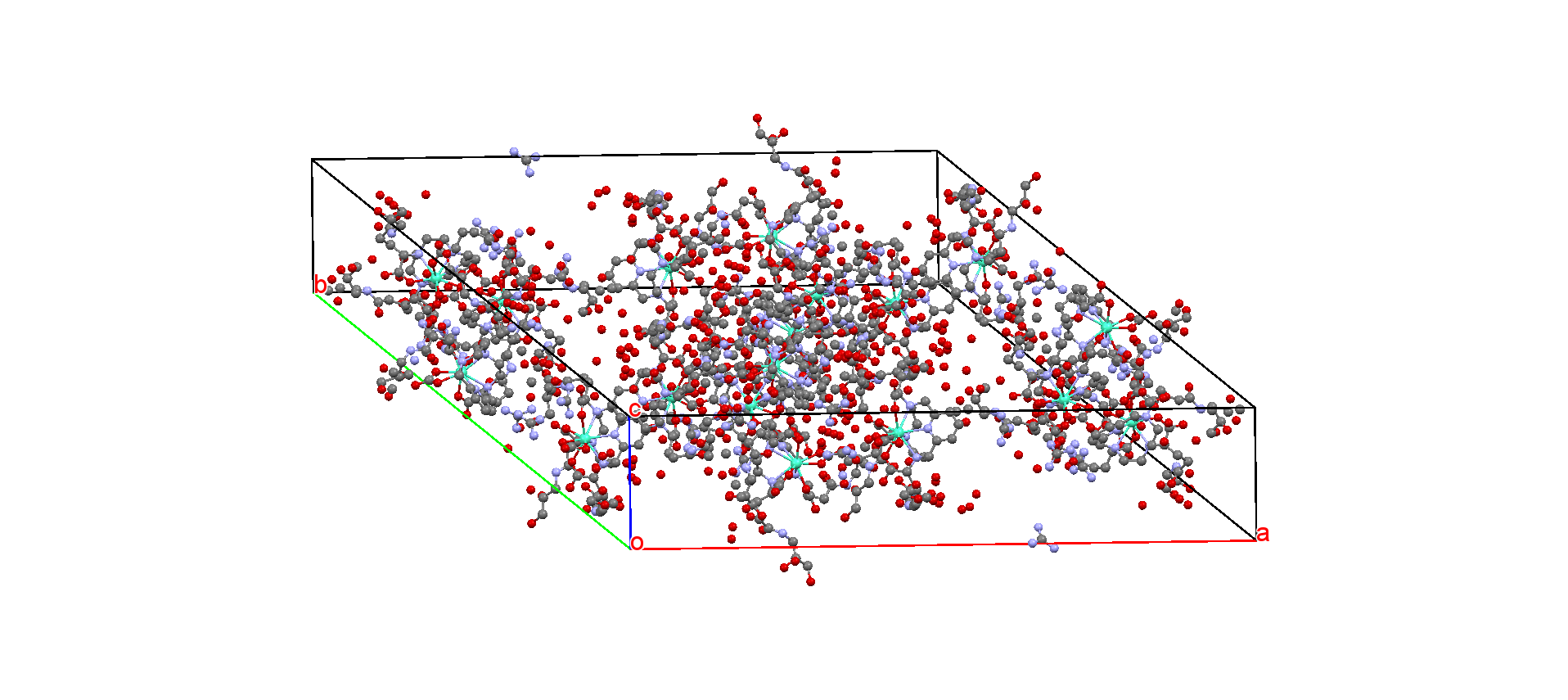


**Figure S24.** Packing diagram of {(C(NH_2_)_3_)_2_[Gd(C_35_H_54_N_7_O_15_)(CO_3_)]}·22H_2_O. Hydrogen atoms are omitted for simplicity.

**Table S14.** Geometric parameters for metal coordination sphere in {(C(NH_2_)_3_)_2_[Gd(C_35_H_54_N_7_O_15_)(CO_3_)]}·22H_2_O. Atom naming is shown in Figure S16.

| **Distances** | **[Å]** | **Angles** | **[°]** | **Angles** | **[°]** | **Angles** | **[°]** |
| --- | --- | --- | --- | --- | --- | --- | --- |
| Gd _1-O2_1 | 2.392(3) | O2_2-Gd_1-O2_1 | 72.91(9) | N1_1-Gd_1-O10_1 | 86.61(10) | O2_1-Gd_1-O1_2 | 89.69(10) |
| Gd_1-O5_1 | 2.308(3) | O10_2-Gd_1-O2_1 | 143.72(9) | N3_1-Gd_1-O10_1 | 131.13(10) | O2_2-Gd_1-O1_2 | 54.71(10) |
| Gd _1-O10_1 | 2.417(3) | N1_1-Gd_1-O2_1 | 127.05(10) | N2_1-Gd_1-O10_1 | 142.39(10) | O10_1-Gd_1-O1_2 | 79.22(10) |
| Gd _1-N1_1 | 2.567(3) | N2_1-Gd_1-O2_1 | 64.80(10) | N4_1-Gd_1-O10_1 | 62.41(10) | N1_1-Gd_1-O1_2 | 84.99(11) |
| Gd _1-N2_1 | 2.641(3) | N3_1-Gd_1-O2_1 | 72.54(9) | N2_1-Gd_1-N1_1 | 62.67(11) | N2_1-Gd_1-O1_2 | 77.08(10) |
| Gd _1-N3_1 | 2.603(3) | N4_1-Gd_1-O2_1 | 139.67(10) | N3_1-Gd_1-N1_1 | 83.95(11) | N3_1-Gd_1-O1_2 | 146.72(10) |
| Gd _1-N4_1 | 2.704(3) | O1_2-Gd_1-O5_1 | 136.70(10) | N4_1-Gd_1-N1_1 | 63.86(11) | N4_1-Gd_1-O1_2 | 130.49(10) |
| Gd _1-O1_2 | 2.390(3) | O2_1-Gd_1-O5_1 | 77.91(9) | N2_1-Gd_1-N3_1 | 69.95(11) | O10_1-Gd_1-O2_2 | 72.52(9) |
| Gd _1-O2_2 | 2.398(3) | O2_2-Gd_1-O5_1 | 82.03(11) | N4_1-Gd_1-N3_1 | 70.46(10) | N1_1-Gd_1-O2_2 | 136.86(11) |
|  |  | O10_1-Gd_1-O5_1 | 86.76(9) |  |  | N2_1-Gd_1-O2_2 | 114.61(10) |
|  |  | N1_1-Gd_1-O5_1 | 132.15(11) |  |  | N3_1-Gd_1-O2_2 | 137.95(10) |
|  |  | N2_1-Gd_1-O5_1 | 130.09(10) |  |  | N4_1-Gd_1-O2_2 | 129.34(10) |
|  |  | N3_1-Gd_1-O5_1 | 67.92(10) |  |  |  |  |
|  |  | N4_1-Gd_1-O5_1 | 73.85(10) |  |  |  |  |

**Table S15.** Hydrogen-bond geometry for {(C(NH_2_)_3_)_2_[Gd(C_35_H_54_N_7_O_15_)(CO_3_)]}·22H_2_O. Many hydrogen bonds involving solvent water molecules are excluded from this table, since the corresponding hydrogens could not be located into electron density maps.

| **D-H···A d(D-H) [Å] d(H···A) [Å] d(D···A) [Å] <(DHA) [°]** |
| --- |
| O16_1-H16C_1^a...O1_1#1 0.84 1.83 2.637(5) 159.7  O15B_1^b-H15B1^b...N3B_1^b#1 0.84 2.48 3.285(16) 161.0  N3A_1^a-H3A_1^a...O15A_1^a#2 0.88 2.39 2.924(11) 119.4  N3A_1^a-H3A_1^a...O12A_1^a#3 0.88 2.13 2.97(2) 160.6  N3B_1^b-H3B_1^b...O15B_1^b#2 0.88 2.48 3.285(16) 151.7  N11B_1^b-H11B_1^b...O10 0.88 2.51 3.26(4) 143.7  O18B_1^b-H18B_1^b...O17B_1^b 0.84 2.38 2.82(3) 113.1  N11C_1^c-H11C_1^c...O10 0.88 2.27 3.12(3) 164.1  N1_3-H1A_3...O1_1#4 0.88 2.11 2.969(4) 163.6  N1_3-H1B_3...O9_1 0.88 1.93 2.809(5) 174.8  N3_3-H3A_3...O16_1#5 0.88 2.19 2.970(5) 146.8  N3_3-H3A_3...O12B_1^b#2 0.88 2.61 3.07(5) 113.7  N3_3-H3B_3...O6_1#2 0.88 2.01 2.876(5) 166.4  N2_3-H2A_3...O3_2 0.88 2.34 2.980(4) 129.8  N2_3-H2B_3...O10_1 0.88 2.14 3.015(5) 176.1  N1_4-H1A_4...O2_2 0.88 1.94 2.799(5) 165.7  N1_4-H1B_4...O16_1#4 0.88 2.02 2.874(5) 163.7  N3_4-H3A_4...O16_1#4 0.88 2.63 3.332(6) 136.9  N3_4-H3B_4...O2_2#2 0.88 2.05 2.860(4) 153.6  N2_4-H2A_4...O3_2 0.88 2.05 2.877(5) 155.8  N2_4-H2B_4...O2_1#2 0.88 2.05 2.911(4) 165.5 |
| Symmetry transformations used to generate equivalent atoms:  #1: -y+4/3,x-y+2/3,z-1/3; #2: -x+y+2/3,-x+4/3,z+1/3; #3: -x+y+2/3,-x+4/3,z-2/3; #4: x,y,z+1; #5: -x+y+2/3,-x+4/3,z+4/3 |

# **4. Solution structure of the Y(III)-complexes with PCTA, *ent*-L_1_ and *ent*-L_2_ ligands**

**Scheme S6.** Structure of [Y(PCTA)], [Y(*ent-*L_1_)]^3-^ and [Y(*ent-*L_2_)]. * and ° represent the stereocenters.

**273 K**

**278 K**

**283 K**

**288 K**

**293 K**

**298 K**

**303 K**

**313 K**

**323 K**

**333 K**

**343 K**

**10**

**9,9’**

**3,3’,4,4’**

**2,6,6’,7,7’**

**Figure S25.** ^1^H NMR spectra of [Y(PCTA)] in the temperature range of 273 – 343 K ([YL]=0.1 M, pH=7.2, 9.4 T, 0.15 M NaCl, H_2_O)


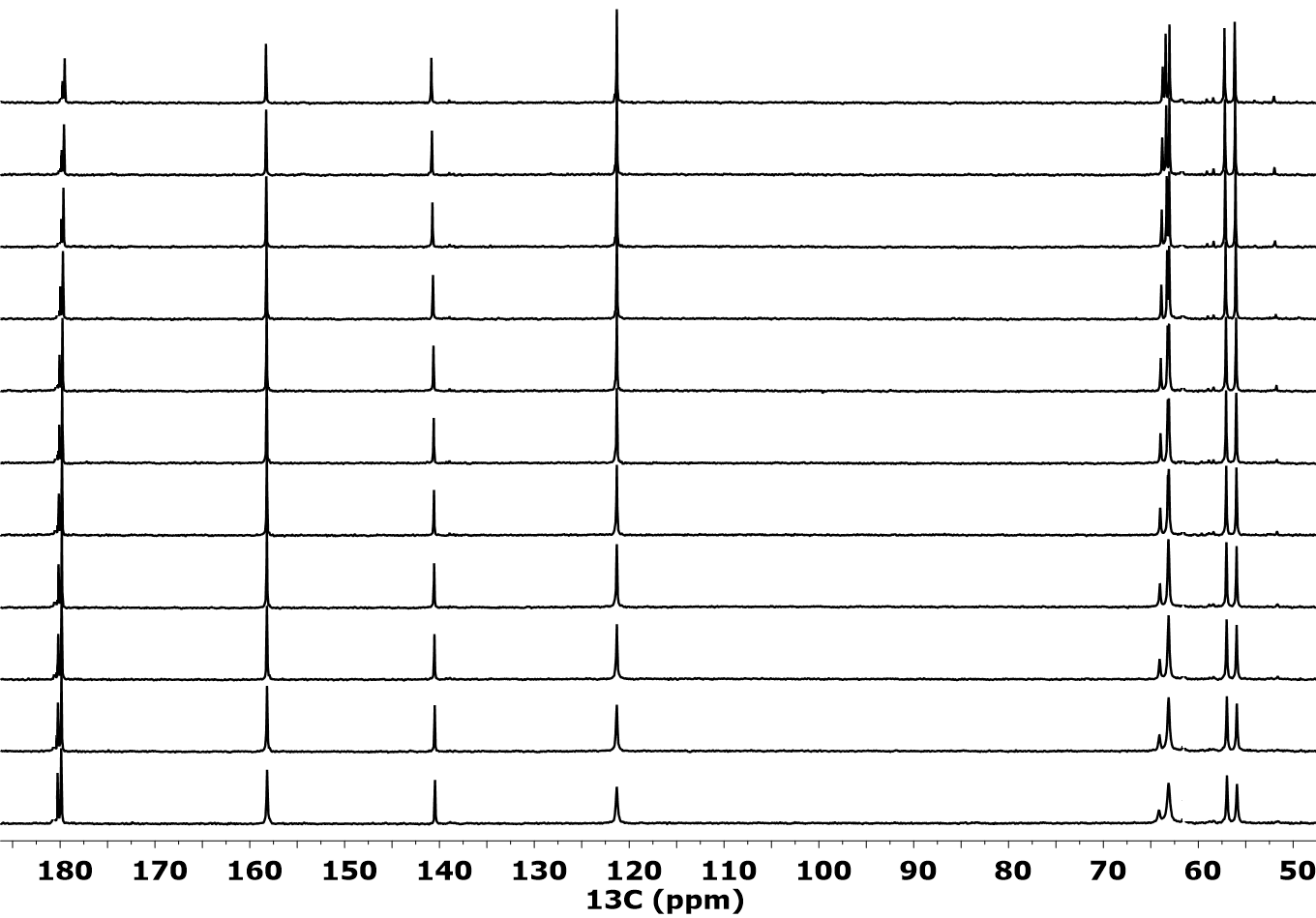


**273 K**

**278 K**

**283 K**

**288 K**

**293 K**

**298 K**

**303 K**

**313 K**

**323 K**

**333 K**

**343 K**

**10**

**9,9’**

**3,3’,4,4’**

**2**

**1**

**5,5’**

**8,8’**

**6,6’,7,7’**

**Figure S26.** ^13^C NMR spectra of [Y(PCTA)] in the temperature range of 273 – 343 K ([YL]=0.1 M, pH=7.2, 9.4 T, 0.15 M NaCl, H_2_O)

**Figure S27.** ^1^H – ^1^H COSY spectra of [Y(PCTA)] at 273 K ([YL]=0.1 M, pH=7.2, 9.4 T, 0.15 M NaCl, H_2_O)

**Figure S28.** ^1^H – ^1^H EXSY spectra of [Y(PCTA)] at 273 K ([YL]=0.1 M, pH=7.2, 9.4 T, D8=0.1 s, 0.15 M NaCl, H_2_O)


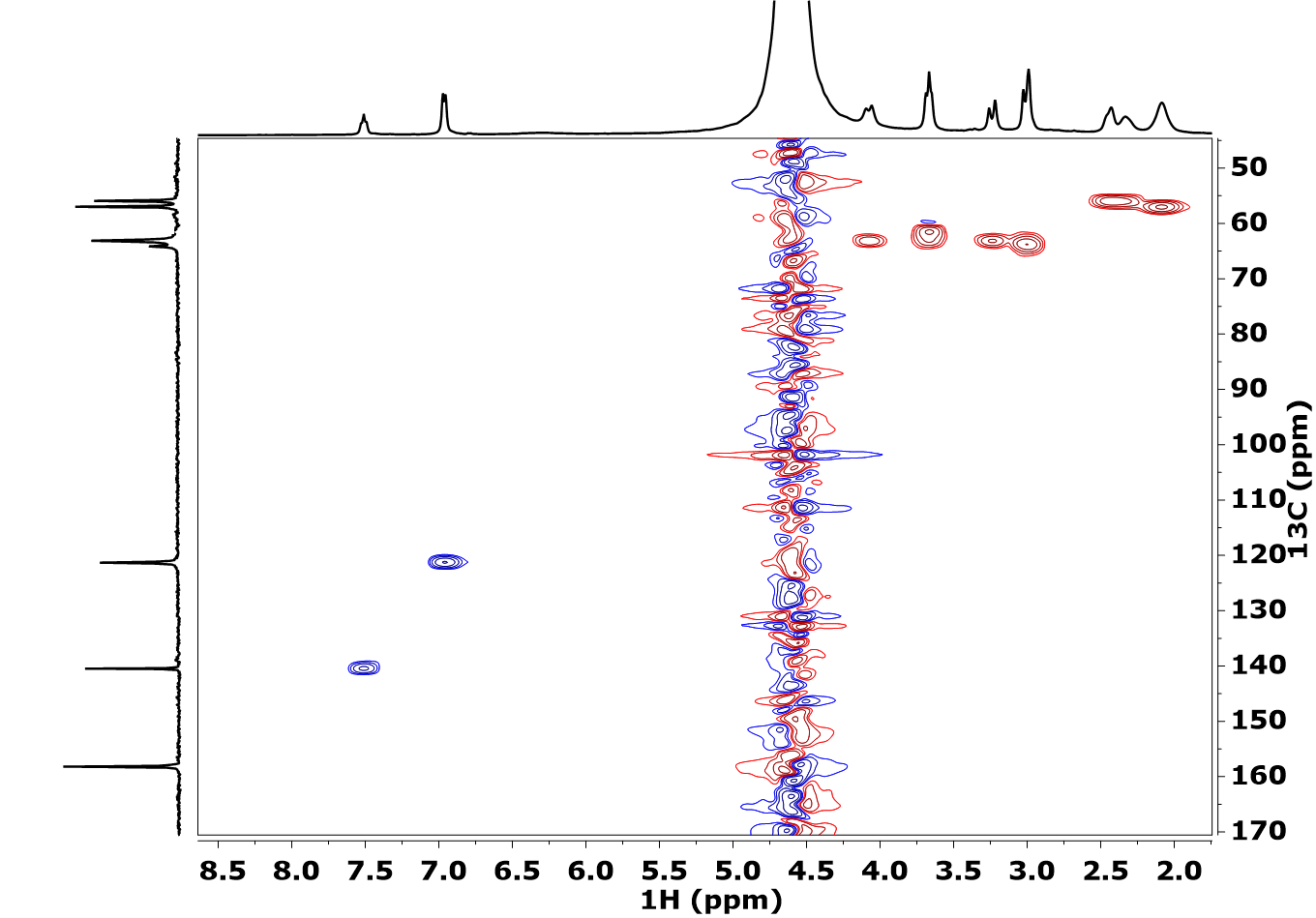


**Figure S29.** ^1^H – ^13^C HSQC spectra of [Y(PCTA)] at 273 K ([YL]=0.1 M, pH=7.2, 9.4 T, 0.15 M NaCl, H_2_O)

**273 K**

**278 K**

**293 K**

**303 K**

**298 K**

**283 K**

**308 K**

**313 K**

**318 K**

**323 K**

**333 K**

**15,15’**

**2,8,8’**

**16**

**4,11,11’, 13,13’**

**6, 6’, 7,7’**

**3,10,10’**

**Figure S30.** ^1^H NMR spectra of [Y(*ent*-L_1_)]^3-^ in the temperature range of 273 – 333 K ([YL]=0.1 M, pH=7.1, 9.4 T, 0.15 M NaCl, H_2_O)

**343 K**

**333 K**

**323 K**

**313 K**

**303 K**

**298 K**

**293 K**

**288 K**

**283 K**

**278 K**

**3, 6,6’,7,7’,11,11’, 13,13’**

**1,5,9,9’,12,12’**

**16**

**15,15’**

**14,14’**

**8,8’**

**4,10,10’**

**2**

**Figure S31.** ^13^C NMR spectra of [Y(*ent*-L_1_)]^3-^ in the temperature range of 278 – 343 K ([YL]=0.1 M, pH=7.1, 9.4 T, 0.15 M NaCl, H_2_O)

**Figure S32.** ^1^H – ^1^H COSY spectra of [Y(*ent*-L_1_)]^3-^ at 298 K ([YL]=0.1 M, pH=7.1, 9.4 T, 0.15 M NaCl, H_2_O)

**Figure S33.** ^1^H – ^13^C HSQC spectra of [Y(*ent*-L_1_)]^3-^ at 298 K ([YL]=0.1 M, pH=7.1, 9.4 T, 0.15 M NaCl, H_2_O)


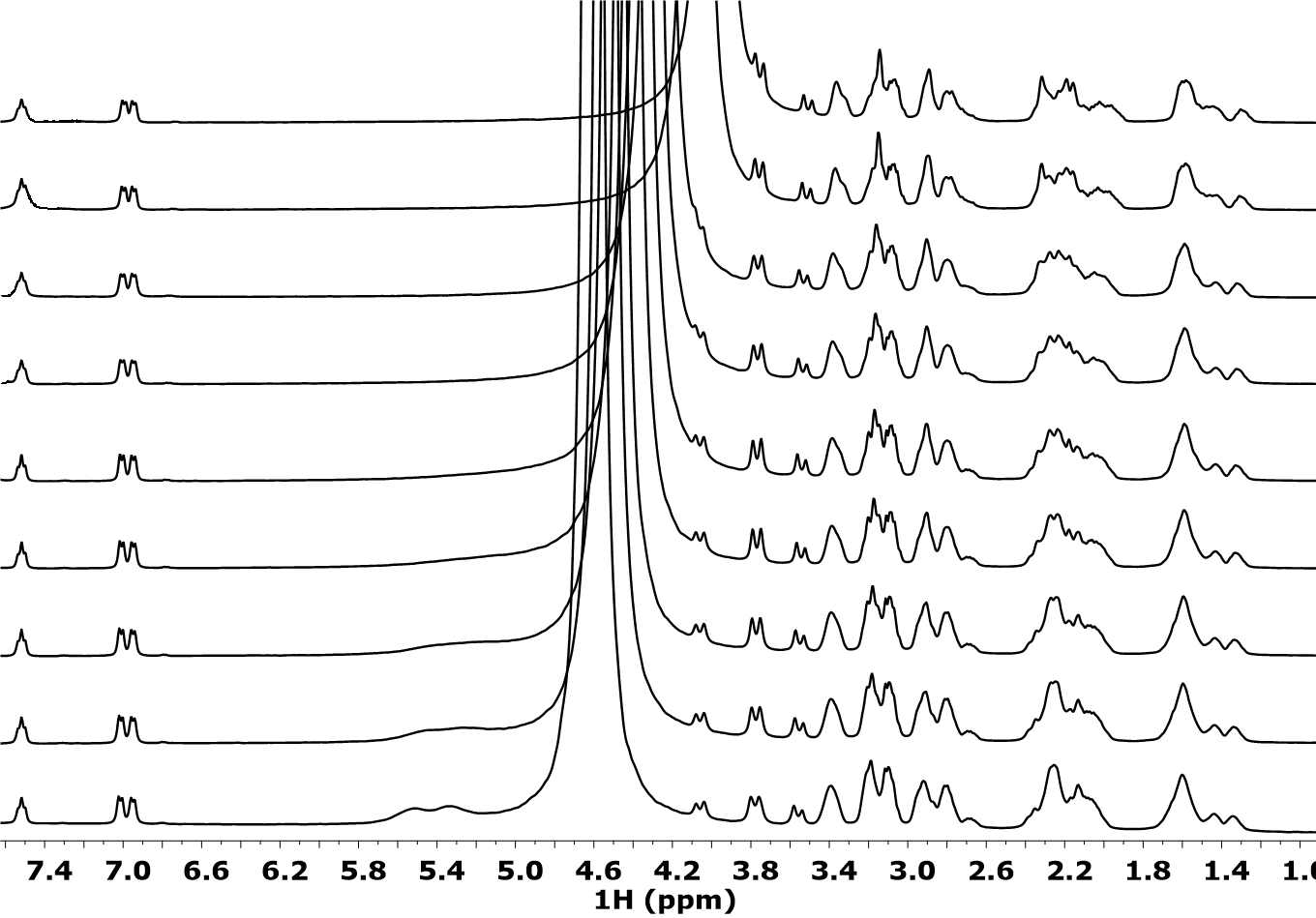


**313 K**

**323 K**

**333 K**

**273 K**

**288 K**

**293 K**

**298 K**

**283 K**

**278 K**

**22**

**3,13,13’**

**9,9’,10,10’**

**2,7,8,11,11’,17,17’, 18,18’**

**21,21’**

**4,6,14,14’,16,16’, 19, 19’**

**Figure S34.** ^1^H NMR spectra of [Y(*ent*-L_2_)] in the temperature range of 273 – 333 K ([YL]=0.1 M, pH=7.2, 9.4 T, 0.15 M NaCl, H_2_O)

**278 K**

**283 K**

**288 K**

**293 K**

**298 K**

**303 K**

**313 K**

**323 K**

**333 K**

**343 K**

**1,5,12,12’,15,15’**

**22**

**21,21’**

**20,20’**

**3,13,13’**

**2,7,11,11’, 17,17’**

**3,4,6,8,9,9’,10,10’, 13,13’,14,14’,16,16’,18,18’**

**Figure S35.** ^13^C NMR spectra of [Y(*ent*-L_2_)] in the temperature range of 278 – 343 K ([YL]=0.1 M, pH=7.2, 9.4 T, 0.15 M NaCl, H_2_O)

**Figure S36.** ^1^H – ^1^H COSY spectra of [Y(*ent*-L_2_)] at 273 K ([YL]=0.1 M, pH=7.2, 9.4 T, 0.15 M NaCl, H_2_O)

**Figure S37.** ^1^H – ^13^C HSQC spectra of [Y(*ent*-L_2_)] at 273 K ([YL]=0.1 M, pH=7.2, 9.4 T, 0.15 M NaCl, H_2_O)

# **5. Thermodynamic properties of the Ca(II)-, Zn(II)-, Cu(II)- and Gd(III)-complexes**

## **5.1 Acid-base properties of *rac*-L_1_, *ent*-L_1_, *rac*-L_2_ and *ent*-L_2_ ligands**

The protonation constants of ligands, defined by **Equation (S1)**, were determined by pH-potentiometry.

$K_{i}^{H}=\frac{[H_{i}L]}{\left[ H_{i-1}L \right][H^{+}]}$ *(S1)*

where i=1, 2…7. The log*K*_i_^H^ values obtained by pH-potentiometry are listed in Table S16. Standard deviations (3σ) are shown in parentheses. In **Table S16**, the log*K*_i_^H^ values of *rac*-L_1_ and *rac*-L_2_ can be regarded as the weighted average of the given protonation constant characterizing the basicity of the related protonation site in the different stereoisomers.

**Table S16.** Portonation constants of the *rac*-L_1_, *ent*-L_1_, *rac*-L_2_, *ent*-L_2_, PCTA, DOTA, HP-DO3A and BT-DO3A ligands (25 °C)

|  | ***ent*-L_2_** | ***rac*-L_2_** | ***ent*-L_1_** | ***rac*-L_1_** | **PCTA** | **DOTA** | **HP-DO3A^f)^** | **BT-DO3A^g)^** |
| --- | --- | --- | --- | --- | --- | --- | --- | --- |
| **I** | 0.15 M NaCl | | | | 1.0 M KCl ^b)^ | 0.15 M NaCl^d)^ | 0.1 M Me_4_NCl | 0.1 M NaCl |
| **log*K*_1_^H^** | 10.43(1) | 9.58 (1) | 10.62 (1) | 10.58 (1)/10.85 ^a)^ | 11.36/9.97^c)^ | 9.14/11.14^e)^ | 11.96 | 9.46 |
| **log*K*_2_^H^** | 5.95 (1) | 5.96 (2) | 6.82 (1) | 6.99 (1)/6.86 ^a)^ | 7.35/6.73^c)^ | 9.21/9.69^e)^ | 9.43 | 9.36 |
| **log*K*_3_^H^** | 4.08 (1) | 4.30 (3) | 5.50 (2) | 5.50 (1)/5.37^a)^ | 3.83/3.22^c)^ | 4.48/4.85^e)^ | 4.30 | 4.17 |
| **log*K*_4_^H^** | 2.05 (2) | 2.86 (3) | 4.89 (2) | 4.82 (1)/4.77 ^a)^ | 2.12/1.40^c)^ | 4.03/3.95^e)^ | 3.26 | 3.02 |
| **log*K*_5_^H^** | − | − | 4.19 (2) | 4.23 (1)/4.24 ^a)^ | 1.29^c)^ | 1.99^d)^ | − | − |
| **log*K*_6_^H^** | − | − | 3.88 (2) | 3.65 (1)/3.4 ^a)^ | − | − | − | − |
| **log*K*_7_^H^** | − | − | 2.30 (2) | 2.34 (2)/1.92 ^a)^ | − | − | − | − |
| **Σlog*K*_i_^H^** | **22.51** | **22.70** | **38.20** | **38.11/37.50** ^a)^ | **25.95/21.32** | **28.85/29.63** | **28.95** | **26.01** |

^a)^ Ref. ^[28]^ (0.1 M Me_4_NCl, 25 °C); ^b)^ Ref. ^[21]^; ^c)^ Ref. ^[67]^ (0.15 M NaCl, 25 °C); ^d)^ Ref. ^[68]^; ^e)^ Ref. ^[29]^ (0.1 M KCl, 25 °C); ^f)^ Ref. ^[32]^; ^g)^ Ref. ^[33]^, DOTA: log*K*_1_^H^=11.45, log*K*_2_^H^=9.64, log*K*_3_^H^=4.60, log*K*_4_^H^=4.11, log*K*_5_^H^=2.29, Σlog*K*_i_^H^=32.09 (0.1 M Me_4_NCl, 25 °C)^[31]^

The protonation sequence of the PCTA ligand has been fully characterized with both spectroscopic and potentiometric methods.^[21,27]^ These studies reveal that the first protonation occurs at the central nitrogen opposing the pyridine ring. The attachment of the second proton takes place at one of the terminal nitrogen atoms nearby the pyridine ring. However, the second protonation process induces the rearrangement of the ring protons by the shift of the first proton from the central to the other terminal nitrogen atom in the neighboring of pyridine ring due to the better charge separation and lower electrostatic repulsion between the protonated donor atoms. Further protonation steps take place at the carboxylate groups attached to the central and the terminal nitrogen atoms, respectively. By taking into account the similarity of *rac*-L_1_, *ent*-L_1_, *rac*-L_2_, *ent*-L_2_ and PCTA, we may safely assume that the first and second protonation of these ligands takes place in analogous manner. However, the third, fourth and fifth protonation of *rac*-L_1_ and *ent*-L_1_ occur on the distant carboxylate groups of the pendant arms. Further protonations of *rac*-L_1_, *ent*-L_1_, *rac*-L_2_ and *ent*-L_2_ takes place at the non-protonated carboxylate moieties attached to the central and terminal macrocyclic nitrogen atoms, respectively.

Comparison of the protonation constants of *rac*-L_1_, *ent*-L_1_, *rac*-L_2_, *ent*-L_2_ with those of PCTA obtained in 0.15 M NaCl solution (Table S16) indicates that the log*K*_1_^H^ value of *rac*-L_1_, *ent*-L_1_, and *ent*-L_2_ is somewhat higher, whereas the first protonation constant of *rac*-L_2_ is comparable with the log*K*_1_^H^ value of the PCTA. On the other hand, the log*K*_2_^H^ values in Table S16 reveal that second protonation constant of *rac*-L_1_ and *ent*-L_1_ is comparable, whereas log*K*_2_^H^ values of *rac*-L_2_ and *ent*-L_2_ are lower than that of PCTA. The higher log*K*_1_^H^ value of *rac*-L_1_ and *ent*-L_1_ can be explained by the higher overall negative charge of these ligand due to the presence of the six carboxylate groups in the pendant arms. Moreover, the higher negative charge can be also responsible for the comparable log*K*_2_^H^ values of the *rac*-L_1_ and *ent*-L_1_ ligands with that of PCTA. However, the higher log*K*_1_^H^ value of *ent*-L_2_ cannot be interpreted by the charge of the ligand due to the presence of the amide function in the distant pendant arms. Higher first protonation constant of *ent*-L_2_ might be explained by the formation of a stronger H-bond between the protonated central nitrogen and the carboxylate groups of α-glutarate pendant arms with the identical chirality (*RRR* or *SSS*). This hypothesis is supported by the 0.8 log*K* unit lower log*K*_1_^H^ value of *rac*-L_2_, which can be interpreted by the weaker H-bond of the protonated central nitrogen with the α-glutarate pendant arms in *RRS* or *SSR*, *RSR* or *SRS* and *SRR* or *RSS* configurations.

In general, the protonation constants of amino-polycarboxylate ligands particularly the log*K*_i_^H^ values obtained in 0.15 M NaCl solution are lower than those determined in solutions, where the constant ionic strength was adjusted by 0.1 M KCl or 0.1 M Me_4_NCl. The lower log*K*_i_^H^ values determined in NaCl solutions are explained by the stronger interaction between the smaller Na^+^ ion and the fully deprotonated ligands respect to the larger K^+^ or Me_4_N^+^ ions. The difference is particularly high for the log*K*_1_^H^ value of macrocyclic ligands like DOTA, HP-DO3A, BT-DO3A and PCTA which form relatively stable complexes with Na^+^ ion (log*K*_Na(DOTA)_=4.38; log*K*_Na(BT-DO3A)_=2.32).^[33,69]^ Surprisingly, the log*K*_1_^H^ value *rac*-L_1_ and *ent*-L_2_ obtained in 0.15 M NaCl and 0.1 M Me_4_NCl are practically identical, which indicates the negligible interaction between the fully deprotonated ligands and Na^+^ ion.

The Σlog*K*_i_^H^ values, presented in Table S16, indicate that the total basicity of the PCTA, DOTA, HP-DO3A and BT-DO3A ligands are significantly lower than those of *rac*-L_1_ and *ent*-L_1_ due to the presence of the three distant basic carboxylate groups in the pendant arms. However, Σlog*K*_i_^H^ values of *rac*-L_2_ and *ent*-L_2_ is comparable with that of the PCTA and significantly lower than the total basicity of DOTA, HP-DO3A and BT-DO3A ligands. By taking into account the Σlog*K*_i_^H^ values of *rac*-L_1_, *ent*-L_1_, *rac*-L_2_, *ent*-L_2_ and PCTA, the stability constants of the Gd(III) complexes with *rac*-L_1_, *ent*-L_1_ are expected to be higher, whereas with *rac*-L_2_ and *ent*-L_2_ ligands are comparable with that of [Gd(PCTA)]. However, in determining the stability constants of the Gd(III) complexes with the chiral pendant arms containing ligand, the geometry of the coordination cage formed by the four nitrogen and the three carboxylate oxygen donor atoms for the Gd(III) ion may also play an important role.

## **5.2 Complexation features of *rac*-L_1_, *ent*-L_1_, *rac*-L_2_ and *ent*-L_2_ ligands**

The stability and protonation constants of Ca(II)-, Zn(II)-, Cu(II)-and Gd(III)-complexes of *rac*-L_1_, *ent*-L_1_, *rac*-L_2_ and *ent*-L_2_, defined by **Equations (S2) and (S3)**, were investigated by pH-potentiometry, spectrophotometry and ^1^H NMR relaxometry at 25 ºC in 0.15 M NaCl solution.

$K_{\mathrm{ML}}=\frac{[ML]}{\left[ M \right][L]}$ *(S2)*

$K_{MH_{i}L}=\frac{[MH_{i}L]}{\left[ MH_{i-1}L \right][H^{+}]}$ *(S3)*

where i=0, 1, 2,…5. The *K*_ML_ and *K*_MHiL_ values characterizing the formation of Ca(II)-, Zn(II)-, Cu(II)- and Gd(III)-complexes with *rac*-L_1_, *ent*-L_1_, *rac*-L_2_ and *ent*-L_2_ ligands have been calculated from the pH-potentiometric titration data obtained at 1:1 metal to ligand concentration ratios. In calculating the equilibrium constants, the best fitting of the mL NaOH – pH data has been obtained by assuming the formation of ML, MHL, MH_2_L, MH_3_L, MH_4_L, MH_5_L and MLH_-1_ species. The formation of the deprotonated [GdLH_-1_]^-^ complexes with *rac*-L_2_ and *ent*-L_2_ ligands takes place at pH>9.0, as indicated by the base consumption in the titration curves. This deprotonation of the Gd(III)-complexes occurs on the inner-sphere water molecule.

The stability constant of Gd(III)-complexes formed with *rac*-L_1_ and *ent*-L_2_ ligand has been determined by the “out-of-cell” technique due to the slow complex formation. It is known that the formation of Ln(III) complexes with DOTA, PCTA and its derivative complexes in the pH range 2 – 6 takes place via the formation of a diprotonated “out-of-cage” complex (e.g. [*Ln(H_2_DOTA)]^+^ [*Ln(H_2_PCTA)]^2+^). In the “out-of-cage” complex only the carboxylate groups are coordinated to the Ln^3+^ ion and two opposite ring nitrogen atoms are protonated.^[21,23,31,70,71]^ The formation of [Ln(DOTA)]^-^, [Ln(PCTA)] and their derivative complexes occur by the slow deprotonation of the “out-of-cage” complex (the rate determining step is the dissociation of the last proton) which is followed by the rearrangement to the final complex. In the equilibrium systems, the presence of the intermediate, the free Ln^3+^ ion and the final Ln(III)-complex should also be taken into account.^[35,72]^ The formation and stability constant of the deprotonated [*Ln(H_2_L)] intermediate can be expressed by **Equation (S4)**.

Ln^3+^ + H_2_L *Ln(H_2_L) *(S4)*

$${{}^{*}K}_{Ln(H_{2}L)}=\frac{[Ln(H_{2}L)]}{\left[ Ln^{2+} \right][H_{2}L]}$$

The “out-of-cage” complexes are formed rapidly, so their stability constants have been determined by direct pH-potentiometric titration of Gd^3+^ – *rac*-L_1_ and Gd^3+^ – *ent*-L_1_ systems at 1:1 metal to ligand concentration ratios in the pH range 1.7 – 7.0. In the “out-of-cell” samples, the measured equilibrium pH values were between 1.7 and 3.0. With the use of the added mL NaOH – pH data, the stability constants of [Gd(*rac*-L_1_)]^3-^ and [Gd(*ent*-L_1_)]^3-^ complex could be calculated. For the complete characterization of the complexation in Gd^3+^ – *rac*-L_1_ and Gd^3+^ –*ent*-L_1_ systems, the Gd(III)-complexes (which are formed completely at about pH=4.0), have been titrated in the pH range 4.0 – 11.0. By taking into account the protonation constant of the rac-L_1_ and ent-L_2_ ligands, the stability constant of the *[Gd(H_2_L)] intermediate and the stability and protonation constant of the [Gd(*rac*-L_1_)]^3-^ and [Gd(*ent*-L_1_)]^3-^ complexes, the species distribution of the Gd^3+^ – *rac*-L_1_ and Gd^3+^ – *ent*-L_2_ systems have been calculated (**Figures S38** and **S39**). Surprisingly, the formation of the diprotonated *[Gd(H_2_L)] intermediate in Gd^3+^ – *rac*-L_1_ and Gd^3+^ – *ent*-L_1_ equilibrium systems takes place in a very low quantity probably due to the relatively high basicity of the distant carboxylate groups (Table S16) and the low stability constants of the diprotonated *[Gd(H_2_*rac*-L_1_)] and *[Gd(H_2_*ent*-L_1_)] intermediates (**Table S18**). The stability constant of the [Gd(*rac*-L_1_)]^3-^ and [Gd(*ent*-L_1_)]^3-^ complexes have also been determined by measuring the relaxivity values of equilibrium solutions obtained for Gd^3+^ – *rac*-L_1_ and Gd^3+^ – *ent*-L_1_ systems in the pH range 1.7 – 3.0 (out-of-cell method). The relaxivity values of Gd^3+^ – *rac*-L_1_ and Gd^3+^ – *ent*-L_1_ systems are presented in Figures S38 and S39. The relaxivity values (*r*_1_) of Gd^3+^ – *rac*-L_1_ and Gd^3+^ – *ent*-L_1_ systems in the pH range 1.7 – 3.0 can be expressed by **Equation (S5)**.

$r_{1}=r_{1}^{w}+r_{1}^{\mathrm{Gd}}[Gd^{3+}]+r_{1}^{\mathrm{Gd}H_{2}L}[Gd{(H}_{2}L)]+r_{1}^{\mathrm{Gd}H_{3}L}[Gd{(H}_{3}L)]+r_{1}^{\mathrm{Gd}H_{4}L}[Gd{(H}_{4}L)]$ *(S5)*

where *r*_1_^Gd^, *r*_1_^GdH2L^, *r*_1_^GdH3L^, *r*_1_^GdH4L^ and *r*_1_^w^ are the relaxivity values of the free Gd^3+^, [Gd(H_2_L)], [Gd(H_3_L)] and [Gd(H_4_L)] species and the aqueous solution in the absence of paramagnetic species. Relaxivity values of the [Gd(H_2_L)], [Gd(H_3_L)] and [Gd(H_4_L)] species formed in Gd^3+^ – *rac*-L_1_ and Gd^3+^ – *ent*-L_1_ systems have been determined by the direct titration of Gd(III)-complexes in the pH range 2.5 – 9.0 (Figures S38 and S39, **Table S17**).

By expressing the concentration of the [Gd(H_2_L)], [Gd(H_3_L)] and [Gd(H_4_L)] species by the stability (log*β*_Gd(H2L)_, log*β*_Gd(H3L)_, log*β*_Gd(H4L)_) and protonation constants (log*K*_GdH2L_, log*K*_GdH3L_, log*K*_GdH4L_), the stability constants (log*K*_GdL_) of [Gd(*rac*-L_1_)]^3-^ and [Gd(*ent*-L_1_)]^3-^ complexes could be calculated.

**Figure S38.** Species distribution and relaxivity values (⯁) of the Gd(III) – *rac*-L_1_ system. The line and the symbols represent the calculated and the experimental relaxivity values, respectively. ([Gd^3+^]=[L]=1.0 mM, 21 MHz, 25 °C, 0.15 M NaCl)

**Figure S39.** Species distribution and relaxivity values (⯁) of the Gd(III) – *ent*-L_1_ system. The line and the symbols represent the calculated and the experimental relaxivity values, respectively. ([Gd^3+^]=[L]=1.0 mM, 21 MHz, 25 °C, 0.15 M NaCl)

The stability constant of Gd(III)-complexes with *rac*-L_2_ and *ent*-L_2_ ligands were determined by the measurement of the proton relaxation rates studying the competition reaction of *rac*-L_2_ and *ent*-L_2_ ligands with AAZTA for Gd^3+^ ion (**Equation (S6)**) at pH=4.0 in 0.15 M NaCl solution. Based on the model calculations, the AAZTA ligand can compete with the *rac*-L_2_ and *ent*-L_2_ ligands for Gd^3+^ (Equation (S6), H_x_L=*rac*-L_2_ and *ent*-L_2_, x=1, 2 and y=2, 3) at pH=4.0 in the presence of 1 – 6 mM AAZTA ([Gd^3+^]=[*rac*-L_2_]=[*ent*-L_2_]=2.0 mM, [AAZTA]=1 - 6 mM, 0.15 M Na, 25 °C). The relaxivity values of the Gd(III) – *rac*-L_2_ – AAZTA and Gd(III) – *ent*-L_2_ – AAZTA systems are shown in **Figures S40** and **S41**, respectively.

[Gd(AAZTA)]^-^ + H_x_L [GdL] + H_y_AAZTA *(S6)*

**Figure S40.** Species distribution and relaxivity values (⯁) of the Gd(III) – *rac*-L_2_ – AAZTA system. The line and the symbols represent the calculated and the experimental relaxivity values, respectively. ([Gd^3+^]=[L]=1.0 mM, pH=4.02, **400 MHz**, 25 °C, 0.15 M NaCl)

**Figure S41.** Species distribution and relaxivity values (⯁) of the Gd(III) – *ent*-L_2_ – AAZTA system. The line and the symbols represent the calculated and the experimental relaxivity values, respectively. ([Gd^3+^]=[L]=1.0 mM, pH=4.09, **400 MHz**, 25 °C, 0.15 M NaCl)

The relaxivity values (*r*_1_) of Gd^3+^ – *rac*-L_2_ – AAZTA and Gd^3+^ – *ent*-L_2_ – AAZTA systems in the at pH=4.0 and 400 MHz can be expressed by **Equation. (S7)**.

$r_{1}=r_{1}^{w}+r_{1}^{\mathrm{GdL}}[GdL]+r_{1}^{\mathrm{Gd}(AAZTA)}[Gd(\mathrm{AAZTA})]$ *(S7)*

where *r*_1_^Gd(AAZTA)^, *r*_1_^GdL^ and *r*_1_^w^ are the relaxivity values of the [Gd(AAZTA)]^-^, [Gd(*rac*-L_2_)] and [Gd(*ent*-L_2_)] complexes and the aqueous solution in the absence of paramagnetic species (Table S17). Considering the protonation constant of AAZTA (AAZTA: log*K*_1_^H^=10.06, log*K*_2_^H^= 6.50, log*K*_3_^H^= 3.77, log*K*_4_^H^=2.33, log*K*_5_^H^=1.51, 0.15 M NaCl, 25 °C),^[47]^ *rac*-L_2_ and *ent*-L_2_ ligands (Table S16), the relaxivity values of [Gd(AAZTA)]^-^, [Gd(*rac*-L_2_)] and [Gd(*ent*-L_2_)] complexes (Table S17) and the stability constant of the [Gd(AAZTA)]^-^ ([Gd(AAZTA)]^-^: log*K*_GdL_=18.93, 0.15 M NaCl, 25 °C),^[47]^ the stability constants (log*K*_GdL_) of [Gd(*rac*-L_2_)] and [Gd(*ent*-L_2_)] complexes could be calculated by fitting the relaxivity values in Figures S40 and S41 to Equation (S7). To complete the characterization of the complexation in Gd^3+^– *rac*-L_2_ and Gd^3+^ – *ent*-L_2_ systems, the relaxivity values of the Gd(III) complexes have been measured at 21 MHz in the pH range 2.5 – 9.0 (0.15 M NaCl, 25 °C). By taking into account the protonation constant of the *rac*-L_2_ and *ent*-L_2_ ligands (Table S16), the stability constant of the [Gd(*rac*-L_2_)] and [Gd(*ent*-L_2_)] complexes (Table S18), the species distribution and the relaxivity values of the Gd^3+^ – *rac*-L_2_ and Gd^3+^ – *ent*-L_2_ systems have been calculated (**Figures S42** and **S43**).

**Figure S42.** Species distribution and relaxivity values (⯁) of the Gd(III) – *rac*-L_2_ system. ([Gd^3+^]=[L]=1.0 mM, 21 MHz, 25 °C, 0.15 M NaCl)

**Figure S43.** Species distribution and relaxivity values (⯁) of the Gd(III) – *ent*-L_2_ system. ([Gd^3+^]=[L]=1.0 mM, 21 MHz, 25 °C, 0.15 M NaCl)

**Table S17.** Relaxivity values of Gd(III) complexes at **21 / 400** MHz and 25 °C in 0.15 M NaCl solution.

| **Species** | ***ent*-L_2_** | ***rac*-L_2_** | ***ent*-L_1_** | ***rac*-L_1_** |
| --- | --- | --- | --- | --- |
| **GdL** | 16.2 ± 0.1 / 11.8 ± 0.1 | 14.8 ± 0.1 / 10.8 ± 0.1 | 12.9 ± 0.1 | 9.61 ± 0.06 |
| **GdHL** | − | − | 12.1 ± 0.1 |
| **GdH_2_L** | − | − | 12.2 ± 0.2 |
| **GdH_3_L** | − | − | 11.8 ± 0.1 | 10.1 ± 0.1 |
| **GdH_4_L** | − | − | − | 11.7 ± 0.1 |

[Gd(AAZTA)]^-^: *r*_1p_=6.56 ± 0.08 mM^-1^s^-1^, *r*_1_^H2O^=0.38 s^-1^, 400 MHz, 25 °C; Gd^3+^: *r*_1p_=12.84 ± 0.04 mM^-1^s^-1^, *r*_1_^H2O^=0.38 s^-1^, 21 MHz, 25 °C.

The stability and protonation constants of Cu(II) complexes with *rac*-L_1_, *ent*-L_1_, *rac*-L_2_ and *ent*-L_2_ have been determined by spectrophotometry. The equilibrium reaction (**Equation (S8)**) has been studied in the [H^+^] range of 0.01 – 1.0 M (the ionic strength was constant I=[Na^+^]+[H^+^]=0.15 in the samples [H^+^]≤0.15 M), where the formation of Cu^2+^, CuH_x-y_L and H_x_L species was assumed ([Cu(r*ac*-L_1_)]^4-^ and [Cu(*ent*-L_1_)]^4-^: x=6 and 7, y=4 and 5; [Cu(*rac*-L_2_)]^4-^ and [Cu(*ent*-L_2_)]^4-^: x=3 and 4, y=1 and 2). Some characteristic absorption spectra are shown in **Figure S44 - S47**.

Cu^2+^ + H_x_L [Cu(H_x–y_L)] + yH^+^ *(S8)*

**Cu^2+^**

**Figure S44.** The absorption spectra of the Cu^2+^ – *rac*-L_1_ system as a function of [H^+^]. The lines and the open symbols represent the experimental and the calculated absorbance values, respectively. The curves and the open symbols represent the experimental and the calculated absorbance values, respectively. ([H^+^] = **1.5 M**, **1.0 M**, **0.70 M**, **0.50 M**, **0.25 M**, **0.13 M**, **0.03 M** and **0.012 M**; [Cu^2+^] = [L] = 0.002 M, [H^+^]≤0.15 M→[Na^+^]+[H^+^]=0.15 M, 25 °C).

**Cu^2+^**

**Figure S45.** The absorption spectra of the Cu^2+^ – *ent*-L_1_ system as a function of [H^+^]. The lines and the open symbols represent the experimental and the calculated absorbance values, respectively. The curves and the open symbols represent the experimental and the calculated absorbance values, respectively. ([H^+^] = **1.5 M**, **1.0 M**, **0.70 M**, **0.50 M**, **0.25 M**, **0.13 M**, **0.03 M** and **0.012 M**; [Cu^2+^] = [L] = 0.002 M, [H^+^]≤0.15 M→[Na^+^]+[H^+^]=0.15 M, 25 °C).

**Cu^2+^**

**Figure S46.** The absorption spectra of the Cu^2+^ – *rac*-L_2_ system as a function of [H^+^]. The lines and the open symbols represent the experimental and the calculated absorbance values, respectively. The curves and the open symbols represent the experimental and the calculated absorbance values, respectively. ([H^+^] = **1.0 M**, **0.70 M**, **0.50 M**, **0.25 M**, **0.13 M**, **0.03 M** and **0.01 M**; [Cu^2+^] = [L] = 0.002 M, [H^+^]≤0.15 M→[Na^+^]+[H^+^]=0.15 M, 25 °C).

**Cu^2+^**

**Figure S47.** The absorption spectra of the Cu^2+^ – *ent*-L_2_ system as a function of [H^+^]. The lines and the open symbols represent the experimental and the calculated absorbance values, respectively. The curves and the open symbols represent the experimental and the calculated absorbance values, respectively. ([H^+^] = **1.0 M**, **0.70 M**, **0.50 M**, **0.25 M**, **0.13 M**, **0.03 M** and **0.01 M**; [Cu^2+^] = [L] = 0.002 M, [H^+^]≤0.15 M→[Na^+^]+[H^+^]=0.15 M, 25 °C).

The protonation constants of Cu(II)-complexes with *rac*-L_1_, *ent*-L_1_, *rac*-L_2_ and *ent*-L_2_ have been determined by pH-potentiometric and spectrophotometric titrations of the complexes in the pH range 1.7 – 7.0. These studies reveal that [Cu(*rac*-L_1_)]^4-^ and [Cu(*ent*-L_1_)]^4-^ complexes can form mono-, di-, tri, tetra- and penta-protonated species via the protonation of all distant and two closer carboxylate groups of the pendant arms. Moreover, the pH potentiometric studies also indicate that the [Cu(*rac*-L_2_)]^-^ and [Cu(*ent*-L_2_)]^-^ complexes can form mono- and di-protonated species via the protonation of two carboxylate groups. Surprisingly the maximum of the absorption band of [Cu(H_5_L)]^+^ species formed in Cu^2+^- *rac*-L_1_ system is very similar to that of the [Cu(H_2_L)]^+^ in Cu^2+^– *rac*-L_2_ system (λ_max_= 730 nm, Figures S44 and S46). Moreover, the maximum of the absorption band for the [Cu(H_5_L)]^+^ species in Cu^2+^ – *ent*-L_1_ system is also similar to that of [Cu(H_2_L)]^+^ in Cu^2+^ – *ent*-L_2_ system (λ_max_= 710 nm, Figures S45 and S47). The maximum of the absorption band is shifted from 730 to 750 nm by the deprotonation of [Cu(H_5_L)]^+^ and [Cu(H_2_L)]^+^ via the formation of [Cu(H_4_L)] and [Cu(HL)] species in Cu^2+^– *rac*-L_1_ and Cu^2+^– *rac*-L_2_ systems (Figures S44 and S46). However, the deprotonation of [Cu(H_5_L)]^+^ and [Cu(H_2_L)]^+^ by the formation of [Cu(H_4_L)] and [Cu(HL)] species practically do not affect the absorption maxima Cu^2+^− *ent*-L_1_ and Cu^2+^− *ent*-L_2_ systems (Figures S45 and S47).

The stability and protonation constants and conditional stability constants of the Ca(II)-, Zn(II)-, Cu(II)- and Gd(III)-complexes with *rac*-L_1_, *ent*-L_1_, *rac*-L_2_ and *ent*-L_2_ ligands obtained by pH-potentiometry, spectrophotometry and ^1^H NMR relaxometry are shown and compared with those of the related PCTA, DOTA, HP-DO3A and BT-DO3A complexes in Table S18. The log*K*_ML_ and log*K*^c^_GdL_, pGd values of Ca(II)-, Zn(II)-, Cu(II)- and Gd(III)-complexes with *rac*-L_1_ and *rac*-L_2_ can be regarded as the weighted average of the stability constant of the metal complexes formed by the different stereoisomers of *rac*-L_1_ and *rac*-L_2_ ligands. Moreover, the log*K*_MHiL_ values of Ca(II)-, Zn(II)-, Cu(II)- and Gd(III)-complexes with *rac*-L_1_ and *rac*-L_2_ are related to the weighted average of the protonation constants characterizing the basicity of the related protonation site in the metal complexes formed with different stereoisomers of *rac*-L_1_ and *rac*-L_2_ ligands.

**Table S18.**  Stability and porotnation constants and conditional stability constants of the Ca(II)-, Zn(II)-, Cu(II)- and Gd(III)-complexes with *rac*-L_1_, *ent*-L_1_, *rac*-L_2_, *ent*-L_2_, PCTA, DOTA, HP-DO3A and BT-DO3A ligands (25 °C)

|  | ***ent*-L_2_** | ***rac*-L_2_** | ***ent*-L_1_** | ***rac*-L_1_** | **PCTA^b)^** | **DOTA^c)^** | **HP-DO3A^g)^** | **BT-DO3A^h)^** |
| --- | --- | --- | --- | --- | --- | --- | --- | --- |
| **I** | 0.15 M NaCl | | | | 1.0 M KCl | 0.1 M KCl | 0.1 M Me_4_NCl | 0.1 M NaCl |
| **log*K*_CaL_** | **10.26 (1)** | **7.84 (2)** | **11.03 (1)** | **10.10 (1)** | **12.72** | **16.37** | **14.83** | **12.1** |
| **log*K*_CaHL_** | 4.16 (2) | 4.86 (9) | 5.59 (2) | 5.72 (1) | 3.79 | 3.60 | − | − |
| **log*K*_CaH2L_** | − | − | 5.01 (2) | 5.34 (2) | − | − | − | − |
| **log*K*_CaH3L_** | − | − | 4.74 (2) | 4.83 (4) | − | − | − | − |
| **log*K*_CaH4L_** | − | − | 4.24 (3) | 4.45 (2) | − | − | − | − |
| **log*K*_ZnL_** | **20.13 (3)** | **18.66 (3)** | **20.77 (2)** | **19.54(2)** | **20.48** | **18.7** | **19.37** | **17.0** |
| **log*K*_ZnHL_** | 3.27 (3) | 3.77 (3) | 5.26 (2) | 5.24 (4) | 3.10 | 5.33 | 3.7 | 4.3 |
| **log*K*_ZnH2L_** | − | − | 4.76 (2) | 5.01 (2) | − | 3.96 | − | − |
| **log*K*_ZnH3L_** | − | − | 4.18 (2) | 3.91 (3) | − | − | − | − |
| **log*K*_ZnH4L_** | − | − | 3.29 (2) | 3.88 (3) | − | − | − | − |
| **log*K*_CuL_** | **21.52 (4)** | **21.04 (5)** | **23.22 (6)** | **23.26(4)** | **18.79** | **22.72** | **22.84** | **19.1** |
| **log*K*_CuHL_** | 3.27 (1) | 4.42 (4) | 5.25 (2) | 5.38 (3) | 3.58 | 4.45 | 3.72 | 3.8 |
| **log*K*_CuH2L_** | 1.44 (1) | 2.58 (5) | 4.72 (2) | 4.80 (2) | − | 3.92 | 2.3 | 2.4 |
| **log*K*_CuH3L_** | − | − | 4.11 (2) | 4.28 (2) | − | − | − | − |
| **log*K*_CuH4L_** | − | − | 3.36 (2) | 3.69 (2) | − | − | − | − |
| **log*K*_CuH5L_** | − | − | 1.31 (2) | 2.33 (2) | − | − | − | − |
| **log*K*_GdL_** | **20.36 (3)** | **18.96 (7)** | **20.17 (9)** | **18.65 (5) / 18.66 (Eu^3+^)^a)^** | **20.39** | **24.7^d)^ / 25.6^e)^** | **23.8** | **18.7** |
| **log*K*_GdHL_** | − | − | 5.28 (1) | 5.32 (2) / 5.34 (Eu^3+^)^a)^ | − | 1.15 | 1.10 | 1.12 |
| **log*K*_GdH2L_** | − | − | 4.52 (1) | 4.43 (3) / 4.54 (Eu^3+^)^a)^ | − | − | − | − |
| **log*K*_GdH3L_** | − | − | 4.15 (2) | 4.35 (3) / 4.12 (Eu^3+^)^a)^ | − | − | − | − |
| **log*K*_GdH4L_** | − | − | − | 2.25 (3) | − | − | − | − |
| **log*K*_GdLH-1_** | 11.66 (1) | 11.15 (4) | − | − | 11.10 | − | 11.36^f)^ | 9.48 |
| ***log*K*_Gd(H2L)_** | − | − | 4.00 (3) | 3.58 (2) | 3.12 | 6.07^f)^ | 5.10^f)^ | 4.53^f)^ |
| **pGd ^i)^** | **18.27** | **17.70** | **17.80** | **16.33 / 16.06 (Eu^3+^)^a)^** | **17.11** | **22.09^d)^ / 20.24 ^e)^** | **18.16** | **15.63** |
| **log*K*^c^_GdL_ ^j)^** | **17.31** | **16.76** | **16.85** | **15.34 / 15.10 (Eu^3+^)^a)^** | **16.15** | **21.14^d)^ / 19.29^e)^** | **17.21** | **14.67** |

^a)^ Ref. ^[28]^ (0.1 M Me_4_NCl, 25 °C); ^b)^ Ref. ^[21]^; ^c)^ Ref. ^[29]^; ^d)^ Ref. ^[30]^ (0.1 M NaCl, 25 °C); ^e)^ Ref. ^[31]^ (0.1 M Me_4_NCl, 25 °C); ^f)^  Ref. ^[35,72]^ (0.15 M NaCl, 25 °C); ^g)^ Ref. ^[32]^; ^h)^ Ref. ^[33]^; ^i)^ pGd=-log[Gd^3+^]_free_, [Gd^3+^]_tot_= 1 μM, [L]_tot_= 10 μM, pH=7.4; ^j)^ *K*_GdL_^c^=*K*_GdL_/1+α_H_, α_H_=*K*_1_^H^[H^+^]+ *K*_1_^H^*K*_2_^H^[H^+^]^2^+… *K*_1_^H^*K*_2_^H^…*K*_n_^H^[H^+^]^n^ and pH=7.4.

The comparison of the log*K*_ML_ values in Table S18 reveals that the Ca(II)-, Zn(II)-, Cu(II)- and Gd(III)-complexes of *ent*-L_1_ and *ent*-L_2_ are generally about 0.5 – 2.0 log*K* unit higher than those of the corresponding *rac*-L_1_ and *rac*-L_2_, whereas log*K*_ML_ values of *rac*-L_1_ and *ent*-L_1_ complexes with Cu^2+^ ion are very similar. Moreover, stability constants of the Ca(II)-, Zn(II)- and Gd(III)-complexes with *ent*-L_1_ and *ent*-L_2_ are very similar. The higher stability of the Ca(II)-, Zn(II)- and Gd(III)-complexes with *ent-*L_1_ and *ent-*L_2_ ligands can be explained by the stronger interactions between the metal ions and the coordinating α-glutarate groups with the identical (*RRR* or *SSS*) chirality.

On the other hand, the stability constants of the Ca(II)-, Zn(II)- and Gd(III)-complexes formed with *rac*-L_1_, *ent*-L_1_, *rac*-L_2_ and *ent*-L_2_ (Table S18) are generally about 0.1 – 6 orders of magnitude lower than those of the corresponding DOTA and HP-DO3A complexes, whereas the log*K*_ML_ values of *ent*-L_1_and *ent*-L_2_ and PCTA complexes formed with Ca^2+^, Zn^2+^ and Gd^3+^ ions are very similar. Surprisingly, the stability constants of Ca(II)-, Zn(II)- and Gd(III)-complexes with the heptadentate *rac*-L_1_, *ent*-L_1_, *rac-*L_2_ and *ent*-L_2_ are comparable or even higher than those of the corresponding complexes formed with the octadentate BT-DO3A ligand. For the calculation of the stability constants (log*K*_ML_) of metal complexes, the protonation constants (log*K*_i_^H^, Table S16) of the ligands should be determined at identical condition. The log*K*_i_^H^ and log*K*_ML_ values were most frequently determined in 0.1 M KCl or 0.1 M Me_4_NCl.^[73]^ The protonation constants of ligands particularly the log*K*_1_^H^ values determined in 0.1 or 0.15 M NaCl solution are generally lower than those obtained in 0.1 M KCl or 0.1 M Me_4_NCl solutions due to the interaction between the smaller Na^+^ ion and the fully deprotonated ligands. The decrease of the log*K*_1_^H^ is exceptionally high for macrocyclic ligands which form relatively stable complexes with Na^+^ (log*K*_Na(DOTA)_=4.38, log*K*_Na(BT-DO3A)_=2.32).^[33,69]^ The metal ion affinity of ligands can be directly compared by the calculations of the conditional stability constant (log*K*^c^_ML_) and pM value at given condition. In order to compare the stability of the Gd(III)-complexes, the conditional stability constants and the pGd values have been calculated for *rac*-L_1_, *ent*-L_1_, *rac*-L_2_, *ent*-L_2_, PCTA, DOTA, HP-DO3A and BT-DO3A complexes (Table S18). The comparison of the log*K*^c^_GdL_ and pGd values reveals that the conditional stability of the Gd(III)-complexes with *ent*-L_1_, *rac*-L_2_ and *ent*-L_2_ are significantly higher than that of [Gd(BT-DO3A)] and comparable with that of [Gd(HP-DO3A)]. Moreover, the log*K*^c^_GdL_ and pGd values of the Gd(III)-complexes with *ent*-L_1_, *rac*-L_2_ and *ent*-L_2_ are 0.6 – 1.2 log*K* unit higher than that of the parent [Gd(PCTA)]. Interestingly, the conditional stability of the [Gd(*ent*-L_1_)] and [Gd(*ent*-L_2_)] are 0.6 - 1.5 log*K* unit higher than those of the related [Gd(*rac*-L_1_)] and [Gd(*rac*-L_2_)]. Based on these evidences, it can be assumed that the Gd^3+^ affinity of *ent*-L_1_ and *ent*-L_2_ are higher than that of *rac*-L_1_ and *rac*-L_2_ due to the stronger interactions between the Gd^3+^ ion and three coordinating α-glutarate groups with the identical chirality (*RRR* or *SSS*) in *ent*-L_1_ and *ent*-L_2_ ligands.

The Ca(II)-, Zn(II)-, Cu(II)- and Gd(III)-complexes of *rac*-L_1_, *ent*-L_1_, *rac*-L_2_ and *ent*-L_2_ similarly to the analogous PCTA complexes can be protonated at low pH values. The log*K*_MHL_ - log*K*_MH2L_ values of Ca(II)-, Zn(II)-, Cu(II)- and Gd(III)-complexes of *rac*-L_1_ and *ent*-L_1_, (Table S18) are very similar to the log*K*_3_^H^ – log*K*_5_^H^ values of the free ligands (Table S16). The experimental evidences clearly indicate that the distant carboxylate groups of the side chains in *rac*-L_1_ and *ent*-L_1_ ligands do not take place in the coordination of metal ions and these functionalities can protonate/deprotonate independently. At lower pH values, the one and two lower protonation constants could be determined for the Ca(II)-, Zn(II)-, Cu(II)-complexes, which might be explained by the presence of one or two weakly coordinated donor atom (a carboxylate oxygen) that can be protonated in the pH range of 2 to 4. The log*K*_GdLH-1_ values of [Gd(*ent*-L_2_)] complexes characterizing the deprotonation of the inner sphere water molecule is significantly higher than that of [Gd(*rac*-L_2_)] and of the parent [Gd(PCTA)], which might be explained by the more compact structure of [Gd(*ent*-L_2_)] due to the stronger interactions between the Gd^3+^ ion and three coordinating α-glutarate groups with the identical chirality (*RRR* or *SSS*) in *ent*-L_2_ ligands.

# **6. Kinetic inertness of the Gd(III)-complexes**

The dissociation reactions of Gd(III)-complexes with the different isomers formed by *rac*-L_1_ and *rac*-L_2_ have been studied by HPLC method (Tables S1 and S2) at 25 °C in 0.01 – 1.0 M HCl solution to guarantee the pseudo-first-order kinetic condition. Area values as a function of time for the dissociation reactions of Gd(III)-complexes formed with the different stereoisomers of *rac*-L_1_ obtained at 25 °C in 1.0 M HCl solution are shown in **Figure S48**.

**Figure S48.** Area values as a function of time for the dissociation reactions of Gd(III)-complexes formed with the different stereoisomers of *rac*-L_1_ at 25 °C in 1.0 M HCl ([GdL]=0.2 mM, A=*SRS-RSR* (⯁), B=*SRR-RSS* (◼), C=*SSS-RRR* (▲) and D=*RRS-SSR* (⚫))

In the presence of HCl excess, the decomplexation of Gd(III)-complexes can be treated as a pseudo-first-order process and the reaction rate can be expressed by **Equation (S9)**, where *k*_d_ is a pseudo-first-order rate constant, [GdL]_t_ and [GdL]_tot_ are the concentrations of the GdL species at time *t* and the total concentration of the complex, respectively.

$-\frac{d[GdL]_{t}}{\mathrm{dt}}=k_{d}[GdL]_{\mathrm{tot}}$ *(S9)*

The rates of the dissociation reactions have been studied at different concentrations of HCl ([HCl]=0.01 – 1.0 M, 25 °C). The *k*_d_ values obtained for the Gd(III)-complexes with the different isomers of *rac*-L_1_ and *rac*-L_2_ ligands as a function of [H^+^] are shown in **Figures S49** and **S50**.

**Figure S49.** Pseudo-first-order rate constant (*k*_d_) characterizes the dissociation of Gd(III)-complexes formed with **A**, **B**, **C** and **D** isomers of *rac*-L_1_ as a function of [H^+^]. ([GdL]=0.2 mM, [H^+^]≤0.15 M→[Na^+^]+[H^+^]=0.15 M, 25 °C).

**Figure S50.** Pseudo-first-order rate constant (*k*_d_) characterizes the dissociation of Gd(III)-complexes formed with **A’**, **B’**, **C’** and **D’** isomers of *rac*-L_2_ as a function of [H^+^]. ([GdL]=0.2 mM, [H^+^]≤0.15 M→[Na^+^]+[H^+^]=0.15 M, 25 °C).

The obtained *k*_d_ pseudo-first order rate constants are directly proportional to the concentration of H^+^. The increase in the *k*_d_ values with increasing concentration of H^+^ can be interpreted in terms of the proton assisted dissociation of Gd(III)-complexes. The dependence of *k*_d_ on [H^+^] can be expressed as a first-order function of [H^+^] which indicates that the exchange can take place by proton-independent (**Equation (S10)**) and proton assisted (**Equation (S12)**) pathways. The proton assisted dissociation of Gd(III)-complexes might be explained by the equilibrium formation of a protonated *Gd(HL) intermediate (**Equation (S11)**), which dissociates spontaneously (Equation (12)). In the case of the Gd(III)-complexes with B isomer of *rac*-L_1_ and B’ isomer of *rac*-L_2_, the *k*_d_ values shows a saturation profile as a function of [H^+^], which can be interpreted by the equilibrium formation and the accumulation of a protonated *Gd(HL) intermediate (Equation (S11)).

GdL Gd^3+^ + H_x_L *(S10)*

*k*_0_

$K_{Gd(HL)}^{H}$

GdL + H^+^ *Gd(HL) *(S11)*

$$K_{Gd(HL)}^{H}=\frac{[Gd\left( \mathrm{HL} \right)]}{\left[ \mathrm{GdL} \right][H^{+}]}$$

Gd(HL) Gd^3+^ + H_x_L *(S12)*

*k*_GdHL_

where *k*_0_ and *k*_GdHL_ are the rate constants characterizing the dissociation of Gd(III)-complexes via spontaneous and proton-assisted reaction pathways, respectively. The *K*^H^_GdHL_ is related to the formation of the *Gd(HL) intermediate via the protonation of Gd(III)-complexes. By considering all the possible pathways and the rate of dissociation of Gd(III)-complexes (Equation (S9)), the pseudo-first-order rate constant (*k*_d_) can be expressed by **Equation (S13)**.

$-\frac{d[GdL]_{t}}{\mathrm{dt}}=k_{0}\left[ \mathrm{GdL} \right]+k_{\mathrm{GdHL}}[GdHL]$ *(S13)*

By taking into account the total concentration of the complex ([GdL]_tot_=[GdL]+[*Gd(HL)]), the protonation constants of the Gd(III)-complexes (*K*^H^_GdHL_, Equation (S11)) and Equation (S13), the pseudo-first-order rate constant (*k*_d_) can be expressed by **Equation (S14)**:

$k_{d}=\frac{k_{0}+k_{1}[H^{+}]}{1+K_{Gd(HL)}^{H}[H^{+}]}$ *(S14)*

where *k*_0_, and *k*_1_ = *k*_GdHL_×*K*^H^_GdHL_ are the rate constants characterizing the spontaneous and proton-assisted dissociation of Gd(III)-complexes. The protonation constant (*K*^H^_GdHL_) of Gd(DOTA)-like complexes are very small (Gd(DOTA): *K*^H^_GdHL_ =14).^[22]^ By considering very low protonation constant of Gd(III)-complexes with A, C and D isomers of *rac*-L_1_ and A’, C’ and D’ isomers of *rac*-L_2_ ligands (*K*^H^_GdHL_<<10), the denominator of Equation (S14) (1>> *K*^H^_GdHL_ [H^+^]) can be neglected, so Equation (S14) can be simplified in the form of **Equation (S15)**. The *k*_0_ and *k*_1_ values of the Gd(III) complexes with A, C and D isomers of *rac*-L_1_, and A’, C’ and D’ isomers of *rac*-L_2_ ligands have been calculated by fitting of the kinetic data (Figures S49 and S50) to Equation (S15).

*k*_d_ = *k*_0_ + *k*_1_[H^+^] *(S15)*

In the fitting procedure the *k*_0_ values obtained are very low and the error in them is very high, indicating that the spontaneous dissociation has no relevant contribution to the overall dissociation rate of the Gd(III)-complexes in our experimental condition ([H^+^]=0.01 – 1.0 M). The *k*_1_ rate and log*K*^H^_GdHL_ protonation constants characterizing the acid-catalyzed decomplexation of Gd(III)-complexes formed with the different stereoisomers of *rac*-L_1_ and *rac*-L_2_ are presented and compared with those of [Gd(PCTA)], [Gd(DOTA)]^-^, [Gd(HP-DO3A)] and [Gd(BT-DO3A)] in **Table S19**.

**Table S19.** Rate (*k*_i_) and equilibrium constants (*K*^H^_Gd(HL)_) and half-lives (*t*_1/2_=ln2/*k*_d_) characterizing the dissociation reactions of Gd(III)-complexes with the different stereoisomers of *rac*-L_1_, *rac*-L_2_, PCTA, DOTA, HP-DO3A and BT-DO3A ligands at 25 °C. Isomers related to the configuration of (*) stereocenters in Scheme S1.

|  | Isomers | *k*_0_ [s^-1^] | *k*_1_ [M^-1^s^-1^] | *K*^H^_Gd(HL)_ [M^-1^] | *k*_d_ [s^-1^] at pH=7.4 | *t*_1/2_ [hour] at pH=7.4 |
| --- | --- | --- | --- | --- | --- | --- |
| **[Gd(*rac*-L_1_)]** | **A (*RSR/SRS*)** | − | (4.6±0.2) ×10^-5^ | − | 1.84×10^-12^ | 1.08×10^8^ |
|  | **B (*RSS/SRR*)** | − | (3.2±0.4) ×10^-4^ | 2.0 ± 0.4 | 1.28×10^-11^ | 1.50×10^7^ |
|  | **C (*RRR/SSS*)** | **−** | **(1.4±0.1) ×10^-6^** | **−** | **5.61×10^-14^** | **3.44×10^9^** |
|  | **D (*RRS/SSR*)** | − | (1.2±0.1) ×10^-5^ | − | 4.95×10^-13^ | 3.89×10^8^ |
| **[Gd(*rac*-L_2_)]** | **A’ (*RSR/SRS*)** | − | (4.7±0.1) ×10^-5^ | − | 1.85×10^-12^ | 1.04×10^8^ |
|  | **B’ (*RSS/SRR*)** | − | (2.2±0.2) ×10^-4^ | 1.3 ± 0.3 | 8.55×10^-12^ | 2.25×10^7^ |
|  | **C’ (*RRS/SSR*)** | − | (6.1±0.1) ×10^-6^ | − | 2.42×10^-13^ | 7.95×10^8^ |
|  | **D’ (*RRR/SSS*)** | **−** | **(4.4±0.2) ×10^-6^** | **−** | **1.74×10^-13^** | **1.11×10^9^** |
| **[Eu(PCTA)] ^a)^** | | − | 5.1🞨10^-4^ | 1.72 | 2.0×10^-11^ | 9.52×10^6^ |
| **[Gd(DOTA)]^-^** | | 6.7×10^-11 b)^ | 1.8 ×10^-6 b)^ /8.4×10^-6 c)^ | 14^d)^ | 7.3×10^-14 b)^ /3.3×10^-14 c)^ | **2.64×10^9 b)^ /5.80×10^8 c)^** |
| **[Gd(HP-DO3A)]** | | − | 2.9×10^-4 b)^ /2.6×10^-4 e)^ | − | 1.2×10^-11 b)^ /1.0×10^-11 e)^ | 1.67×10^7 b)^ /1.86×10^7 e)^ |
| **[Gd(BT-DO3A)] ^e)^** | | − | 3.4×10^-5^ | − | 1.35×10^-12^ | 1.42×10^8^ |

^a)^ Ref. ^[21]^ (1.0 M KCl, 25 °C);  ^b)^ Ref. ^[5]^ (0.15 M NaCl, 25 °C); ^c)^ Ref. ^[34]^ (25 °C); ^d)^ Ref. ^[23]^ (3.0 M NaClO_4_, 25 °C); ^e)^ Ref. ^[33]^ (25 °C)

# **7. Relaxation properties of the Gd(III)-complexes**

**Figure S51.** ^1^H NMRD profiles of [Gd(*ent*-L_1_)]^3-^ at 283, 298 and 310 K in water ([Gd^3+^] = 5.77 mM, pH=6.9).

**Figure S52.** ^1^H NMRD profiles of [Gd(*rac*-L_1_)]^3-^ at 283, 298 and 310 K in water ([Gd^3+^] = 5.74 mM, pH=7.2).

**Figure S53.** ^1^H NMRD profiles of [Gd(*ent*-L_2_)] at 283, 298 and 310 K in water ([Gd^3+^] = 4.08 mM, pH=6.5).

**Figure S54**. Plot of the observed relaxation rate of [Gd(*ent*-L_2_)] as a function of [phosphate], [lactate], [carbonate] and [citrate] in water ([Gd^3+^] = 1.0 mM, pH = 7.0, 60 MHz, 298 K).

**Table S20**. Relaxivities (1.5 T) and parameters from the analysis of ^1^H NMRD and ^17^O NMR data.^a)^

| **Parameters** | **[Gd(*rac*-L_1_)]^3-^** | **[Gd(*ent*-L_1_)]^3-^** | **[Gd(*ent*-L_2_)]** |
| --- | --- | --- | --- |
| ***r*_1_^310K^ [mM^-1^s^-1^]** | 6.8 | 8.6 | 12.6 |
| ***Δ*^2^ / 10^19^ [s^-2^]** | 2.1±0.1 | 1.9±0.1 | 2.4±0.1 |
| ***τ*_v_ [ps]** | 34±2 | 37±1 | 38±1 |
| ***E*_v_ [*k*J mol^-1^]** | 1.0^b)^ | 1.0^b)^ | 1.0^b)^ |
| ***τ*_M1_ [ns]** | 103±5 | 144±4 | 155±3 |
| **Δ*H*_M1_ [*k*J mol^-1^]** | 27±1 | 31±2 | 31±1 |
| ***τ*_M2_ [ns]** | 14±2 | 58±5 | 77±5 |
| **Δ*H*_M2_ [*k*J mol^-1^]** | 14±3 | 19±2 | 23±1 |
| ***τ*_R_ [ps]** | 98±2 | 96±3 | 127±4 |
| **^SS^*τ*_R_ [ps]** | / | 68±2 | 82±1 |
| ***E*_R_  [*k*J mol^-1^]** | 22^b)^ | 22^b)^ | 22^b)^ |
| **^SS^*E*_R_  [*k*J mol^-1^]** | / | 18^b)^ | 18^b)^ |
| ***A*_O1_/h / 10^6^ [rad s^-1^]** | -3.5±0.1 | -3.8±0.1 | -3.8±0.1 |
| ***A*_O2_/h / 10^6^ [rad s^-1^]** | -2.5±0.1 | -2.7±0.1 | -2.5±0.1 |
| ***q*** | 2^b)^ | 2^b)^ | 2^b^ |
| ***r* [Å]** | 3.0^b)^ | 3.0^b)^ | 3.0^b)^ |
| **^SS^*q*** | / | 2.5±0.1 | 4.5±0.2 |
| **^SS^*r*  [Å]** | / | 3.6^b)^ | 3.6^b)^ |
| ***a* [Å]** | 4.0^b)^ | 4.0^b)^ | 4.0^b)^ |
| ***D* / 10^-5^ [cm^2^ s^-1^]** | 2.24^b)^ | 2.24^b)^ | 2.24^b)^ |

^a)^ 298 K, ^b)^ fixed in the fitting procedure.

**Table S21.** Relaxivity values for [Gd(*ent*-L_2_)] in saline solution and human plasma at 310 K

|  | ***r*_1_ [mM^-1^s^-1^]** | | | | | |
| --- | --- | --- | --- | --- | --- | --- |
|  | **0.47 T** | | **1.41 T** | | **3 T** | |
|  | **Saline solution** | **Human plasma** | **Saline solution** | **Human plasma** | **Saline solution** | **Human plasma** |
| **[Gd(*ent*-L_2_)]** | 12.6 ± 0.1 | 13.6 ± 0.1 | 12.6 ± 0.1 | 13.4 ± 0.2 | 12.4 ± 0.1 | 13.1 ± 0.2 |


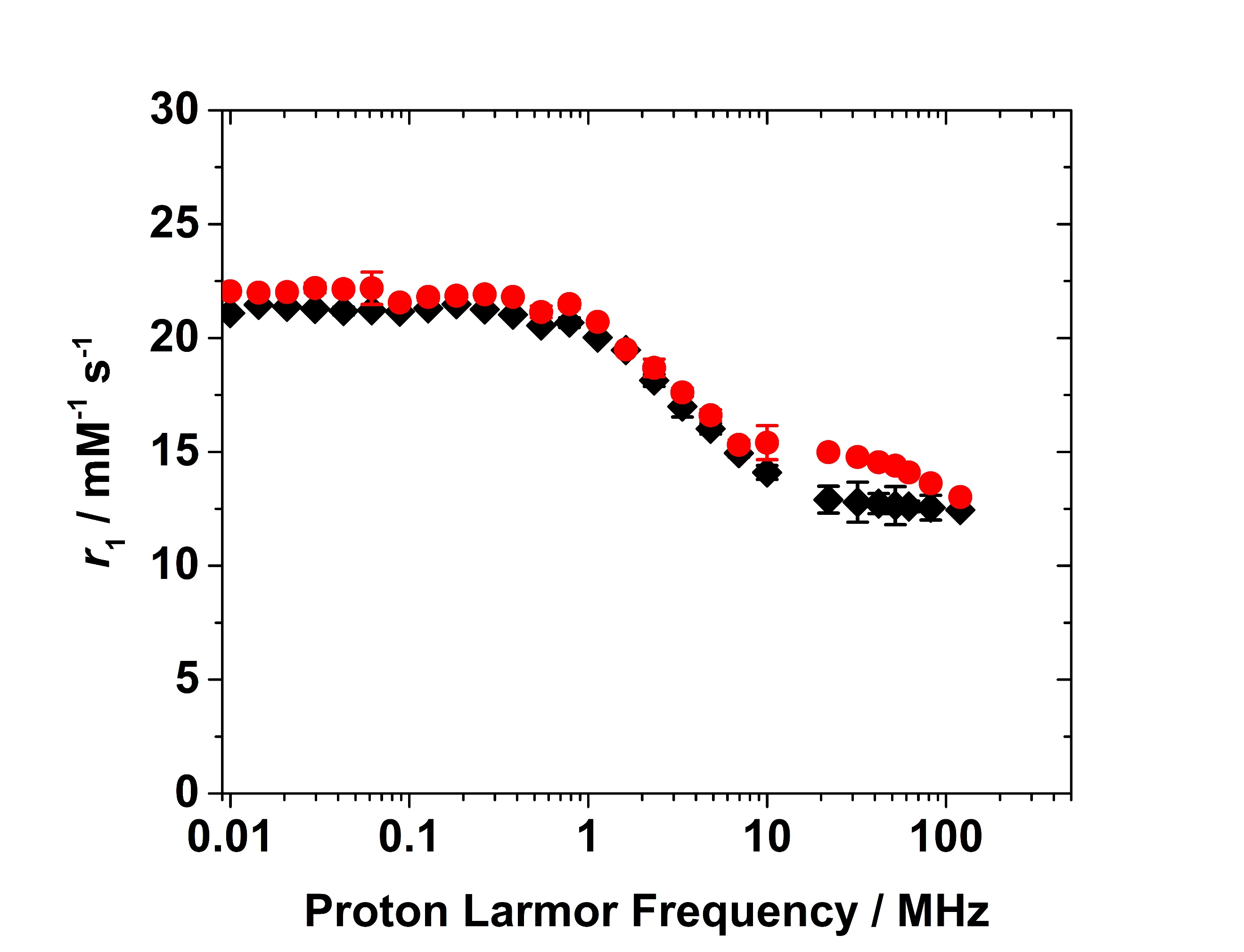


**Figure S55.** ^1^H NMRD profiles of [Gd(*ent*-L_2_)] at 310 K in water (black) and Seronorm (red)


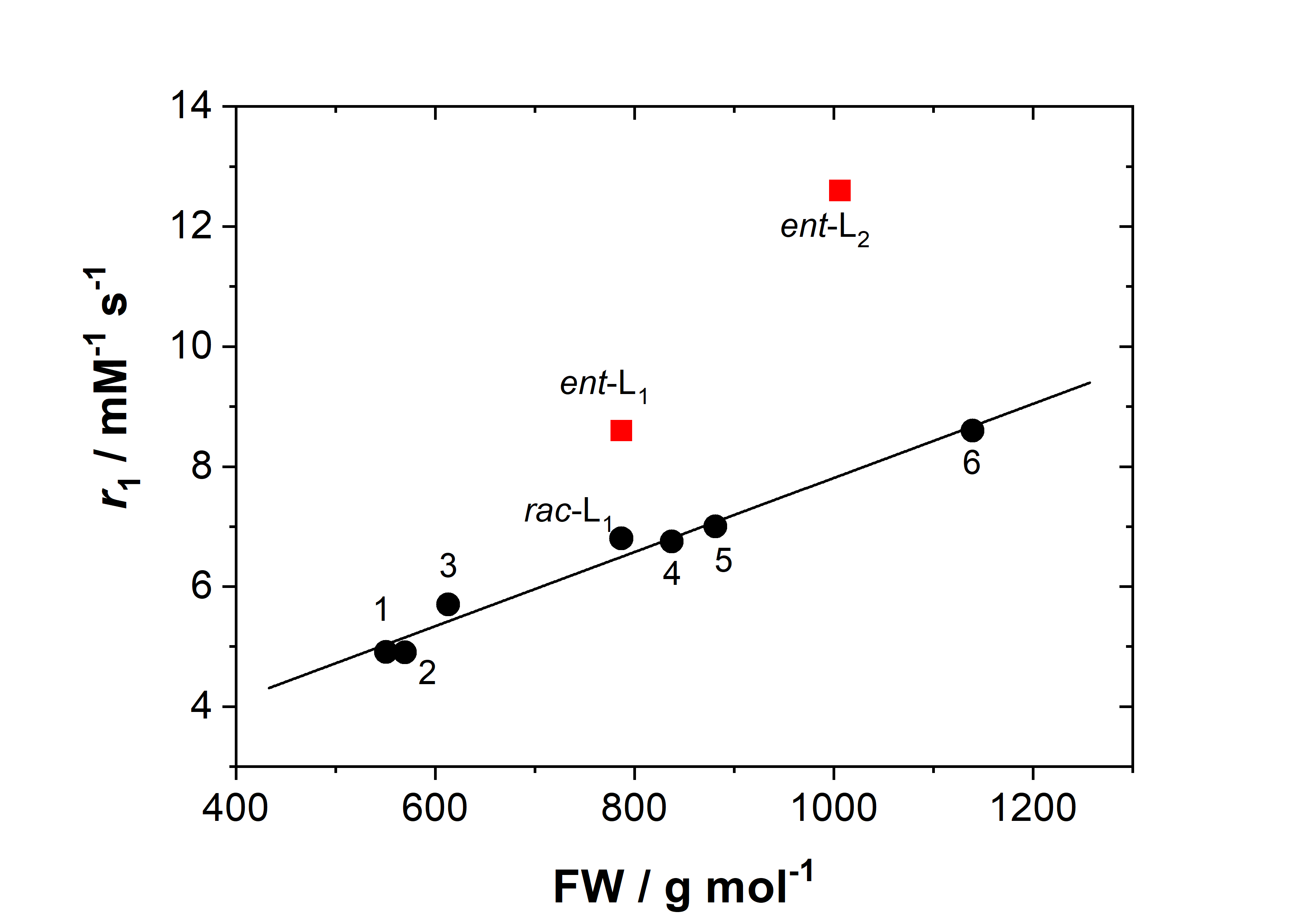


**Figure S56.** ^1^H relaxivity versus molecular mass for *q* = 2 Gd(III)-complexes with ligands AAZTA (1, Ref. ^[74]^), PCTA (2, Ref. ^[19]^), CyPic3A (3, Ref. ^[75]^), trenHOPY (4, Ref. ^[76]^), tris(2-hydroxymethyl)-TRENMe-3,2-HOPO (5, Ref. ^[77]^) and PCTA_2_ (6, Ref. ^[78]^) at 1.5 T and 310 K

# **8. References**

[52] R. Napolitano, L. Lattuada, Z. Baranyai, N. Guidolin, G. Marazzi (Bracco Imaging SpA), *Gadolinium bearing PCTA-based contrast agents, Int. Pat. Appl. WO2020/030618 A1.*, **2020**.

[53] M. Port (Guerbet), *Compounds comprising short aminoalcohol chains and metal complexes for medical imaging, Int. Pat. Appl. WO2007/042506 A1.*, **2007**.

[54] J. Serrano-Plana, A. Aguinaco, R. Belda, E. García-España, M. G. Basallote, A. Company, M. Costas, *Angew. Chem. Int. Ed.* **2016**, *55*, 6310.

[55] F. Travagin, M. L. Macchia, T. Grell, J. Bodnár, Z. Baranyai, F. Artizzu, M. Botta, G. B. Giovenzana, *Dalton Trans.* **2024**, *53*, 1779.

[56] R. Negri, Z. Baranyai, L. Tei, G. B. Giovenzana, C. Platas-Iglesias, A. C. Benyei, J. Bodnar, A. Vagner, M. Botta, *Inorg. Chem.* **2014**, *53*, 12499.

[57] G. M. Sheldrick, *Acta Crystallogr. A: Found. Adv.* **2015**, *71*, 3.

[58] A. Altomare, G. Cascarano, C. Giacovazzo, A. Guagliardi, M. C. Burla, G. Polidori, M. Camalli, *J. Appl. Cryst.* **1994**, *27*, 435.

[59] G. M. Sheldrick, *Acta Crystallogr. C: Struct. Chem.* **2015**, *71*, 3.

[60] P. Emsley, B. Lohkamp, W. G. Scott, K. Cowtan, *Acta Cryst. D* **2010**, *66*, 486.

[61] A. L. Spek, *Acta Cryst. C* **2015**, *71*, 9.

[62] S. Parsons, H. D. Flack, T. Wagner, *Acta Crystallogr. B: Struct. Sci. Cryst. Eng. Mater.* **2013**, *69*, 249.

[63] L. J. Farrugia, *J. Appl. Cryst.* **2012**, *45*, 849.

[64] C. F. Macrae, I. Sovago, S. J. Cottrell, P. T. A. Galek, P. McCabe, E. Pidcock, M. Platings, G. P. Shields, J. S. Stevens, M. Towler, P. A. Wood, *J. Appl. Cryst.* **2020**, *53*, 226.

[65] L. Schrodinger, *The PyMOL Molecular Graphics System*, Schrödinger, LLC, New York **2015**.

[66] A. Riesen, M. Zehnder, T. A. Kaden, *Helv. Chim. Acta* **1986**, *69*, 2067.

[67] Z. Garda, E. Molnár, F. K. Kálmán, R. Botár, V. Nagy, Z. Baranyai, E. Brücher, Z. Kovács, I. Tóth, G. Tircsó, *Front. Chem.* **2018**, *6*.

[68] Z. Baranyai, Z. Palinkas, F. Uggeri, E. Brucher, *Eur. J. Inorg. Chem.* **2010**, 1948.

[69] R. Delgado, J. J. da Silva, *Talanta* **1982**, *29*, 815.

[70] S. L. Wu, W. D. Horrocks, *Inorg. Chem.* **1995**, *34*, 3724.

[71] L. Burai, I. Fábián, R. Király, E. Szilágyi, E. Brücher, *J. Chem. Soc., Dalton Trans.* **1998**, 243.

[72] M. Perez-Malo, G. Szabo, E. Eppard, A. Vagner, E. Brucher, I. Toth, A. Maiocchi, E. H. Suh, Z. Kovacs, Z. Baranyai, F. Rosch, *Inorg. Chem.* **2018**, *57*, 6107.

[73] A. E. Martell, R. M. Smith, *Critical Stability Constants*, Vol. 1–5, Plenum Press, New York **1974**.

[74] D. Lalli, F. Carniato, L. Tei, C. Platas-Iglesias, M. Botta, *Inorg. Chem.* **2022**, *61*, 496.

[75] E. M. Gale, N. Kenton, P. Caravan, *Chem. Commun.* **2013**, *49*, 8060.

[76] C. J. Sunderland, M. Botta, S. Aime, K. N. Raymond, *Inorg. Chem.* **2001**, *40*, 6746.

[77] S. Hajela, M. Botta, S. Giraudo, J. Xu, K. N. Raymond, S. Aime, *J. Am. Chem. Soc.* **2000**, *122*, 11228.

[78] L. Leone, L. Guarnieri, J. Martinelli, M. Sisti, A. Penoni, M. Botta, L. Tei, *Chem.-Eur. J.* **2021**, *27*, 11811.
